# Development of a Dihydroquinoline–Pyrazoline GluN2C/2D-Selective Negative Allosteric Modulator of the *N*-Methyl-d-aspartate Receptor

**DOI:** 10.1021/acschemneuro.3c00181

**Published:** 2023-08-11

**Authors:** Michael P. D’Erasmo, Nicholas S. Akins, Peipei Ma, Yao Jing, Sharon A. Swanger, Savita K. Sharma, Perry W. Bartsch, David S. Menaldino, Paul J. Arcoria, Thi-Thien Bui, Alexandre Pons-Bennaceur, Phuong Le, James P. Allen, Elijah Z. Ullman, Kelsey A. Nocilla, Jing Zhang, Riley E. Perszyk, Sukhan Kim, Timothy M. Acker, Azmain Taz, Samantha L. Burton, Kevin Coe, Russell G. Fritzemeier, Nail Burnashev, Hongjie Yuan, Dennis C. Liotta, Stephen F. Traynelis

**Affiliations:** † Department of Chemistry, Emory University, Atlanta, Georgia 30322, United States; ‡ Department of Pharmacology and Chemical Biology, Emory University, Atlanta, Georgia 30322, United States; § INMED, INSERM, Aix Marseille University, 13284 Marseille, France; ∥ Janssen Research & Development, LLC, San Diego, California 92121, United States

**Keywords:** NR2C, NR2D, blood–brain barrier, tuberous sclerosis complex, seizure, epilepsy

## Abstract

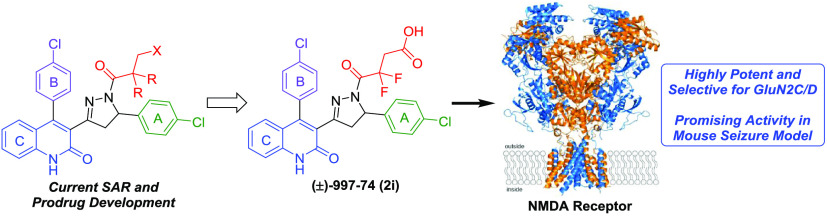

Subunit-selective inhibition of *N*-methyl-d-aspartate receptors (NMDARs) is a promising therapeutic strategy for several neurological disorders, including epilepsy, Alzheimer’s and Parkinson’s disease, depression, and acute brain injury. We previously described the dihydroquinoline–pyrazoline (DQP) analogue **2a** (**DQP-26**) as a potent NMDAR negative allosteric modulator with selectivity for GluN2C/D over GluN2A/B. However, moderate (<100-fold) subunit selectivity, inadequate cell-membrane permeability, and poor brain penetration complicated the use of **2a** as an *in vivo* probe. In an effort to improve selectivity and the pharmacokinetic profile of the series, we performed additional structure–activity relationship studies of the succinate side chain and investigated the use of prodrugs to mask the pendant carboxylic acid. These efforts led to discovery of the analogue (*S*)-(−)-**2i**, also referred to as (*S*)-(−)-**DQP-997**-**74**, which exhibits >100- and >300-fold selectivity for GluN2C- and GluN2D-containing NMDARs (IC_50_ 0.069 and 0.035 μM, respectively) compared to GluN2A- and GluN2B-containing receptors (IC_50_ 5.2 and 16 μM, respectively) and has no effects on AMPA, kainate, or GluN1/GluN3 receptors. Compound (*S*)-(−)-**2i** is 5-fold more potent than (*S*)-**2a**. In addition, compound **2i** shows a time-dependent enhancement of inhibitory actions at GluN2C- and GluN2D-containing NMDARs in the presence of the agonist glutamate, which could attenuate hypersynchronous activity driven by high-frequency excitatory synaptic transmission. Consistent with this finding, compound **2i** significantly reduced the number of epileptic events in a murine model of tuberous sclerosis complex (TSC)-induced epilepsy that is associated with upregulation of the GluN2C subunit. Thus, **2i** represents a robust tool for the GluN2C/D target validation. Esterification of the succinate carboxylate improved brain penetration, suggesting a strategy for therapeutic development of this series for NMDAR-associated neurological conditions.

## Introduction

*N*-Methyl-d-aspartate receptors (NMDARs) belong to the family of ionotropic glutamate receptors that mediate excitatory neurotransmission throughout the mammalian central nervous system (CNS).^1^ These receptors play a role in a variety of neurological processes, including synaptic plasticity, memory formation, neuronal development, and axonal guidance.^2−4^ Physiological dysfunction or overactivation of these receptors have been implicated in a diverse range of neurological and psychiatric disorders, such as Alzheimer’s and Parkinson’s disease, epilepsy, depression, schizophrenia, and acute brain injury.^4−5,7^ Hence, multiple structure–activity relationship (SAR) studies and drug development efforts have been dedicated toward understanding the functional and pharmacological requirements for NMDAR modulation.^4,8−13^ However, with the exception of the anesthetic ketamine and low affinity channel blockers memantine and dextromethorphan, inhibitors of NMDARs that do not distinguish between different subunits have been unsuccessful when tested in the clinic due to adverse reactions from strong NMDAR inhibition and off-target effects (e.g., refs (14,15)). Nevertheless, enormous therapeutic potential exists for the development of safe and effective small-molecule drugs that modulate NMDAR function.

**Figure 1:**
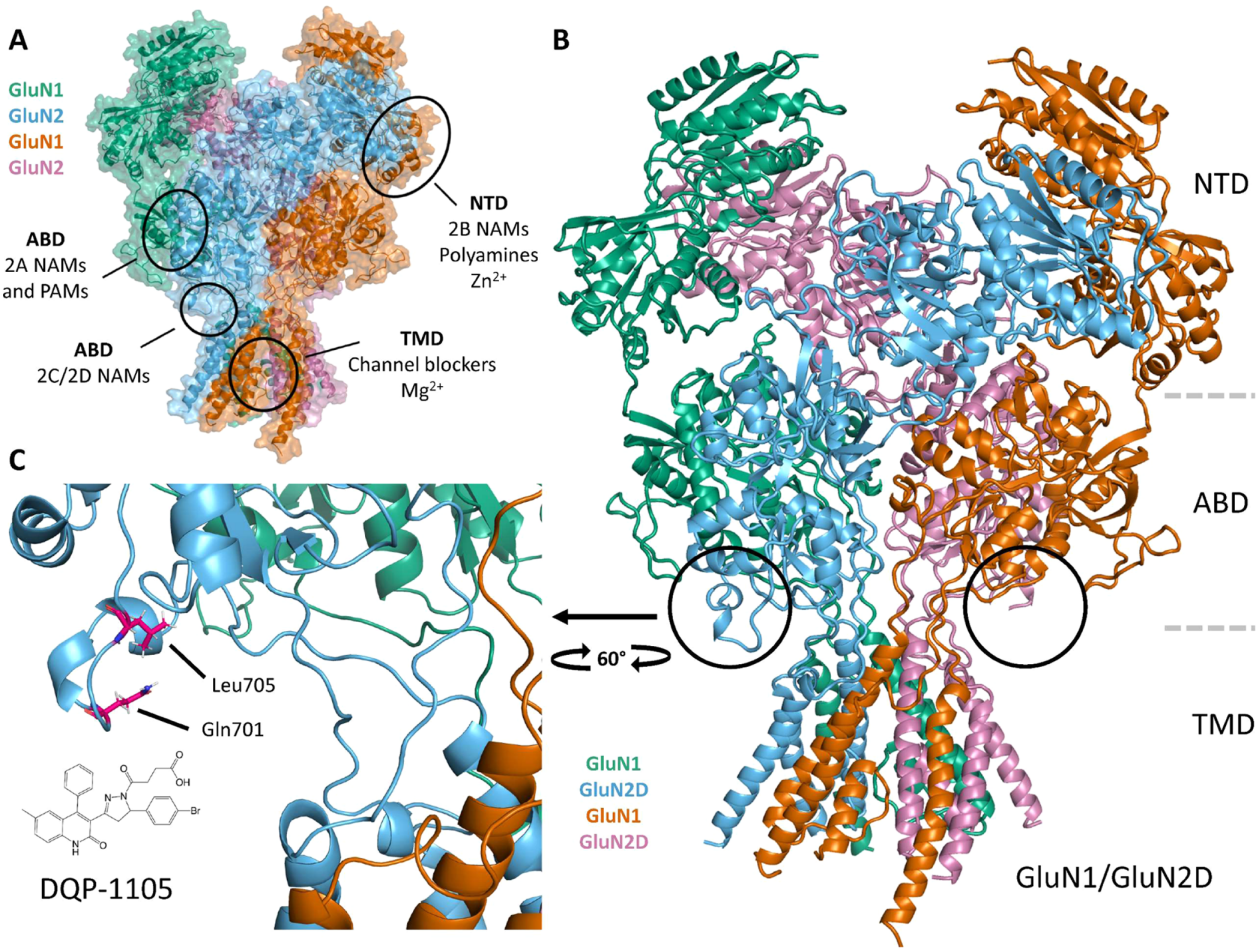
Structure of the GluN1/GluN2D. (A) Model of the GluN1/GluN2D NMDAR (Strong et al.^49^) with the intracellular *C*-terminal domain omitted. Known NMDAR modulator sites are shown. Green and orange chains are GluN1, while blue and magenta chains are GluN2 (see the Methods section). (B) Expanded ribbon structure shown for the homology model of the GluN1/GluN2D heteromeric tetrameric complex in panel (A). (C) Key residues (Gln701, Leu705) that are critical for **DQP-1105** activity (Acker et al.^32^) and **QNZ-46** activity (Hansen and Traynelis^42^) are shown on GluN1/GluN2D NMDA receptors.

Structurally, NMDARs are heterotetrameric complexes of two GluN1 and two GluN2 subunits arranged to create a central ion channel pore permeable to Na^+^, K^+^, and Ca^2+^. Activation of the receptor requires the binding of both glycine to the GluN1 subunit and l-glutamate to the GluN2 subunit, and relief of a voltage-dependent Mg^2+^ block allows cation entry into the cell.^4^ Each subunit comprises four semiautonomous domains: the extracellular amino terminal domain (ATD, also known as NTD), the agonist binding domain (ABD), the pore-forming transmembrane domain (TMD), and the intracellular carboxyl terminal domain (CTD) (Figure 1).^4,12^ The GluN2 subunit is encoded by four genes, giving rise to GluN2A, GluN2B, GluN2C, and GluN2D isoforms. These GluN2 subtypes show different spatiotemporal expression patterns in the brain and facilitate the functional diversity associated with NMDARs.^16−21^ Therefore, numerous research efforts have focused on developing small molecules capable of controlling and distinguishing individual GluN2 subunits.

**Figure 2:**
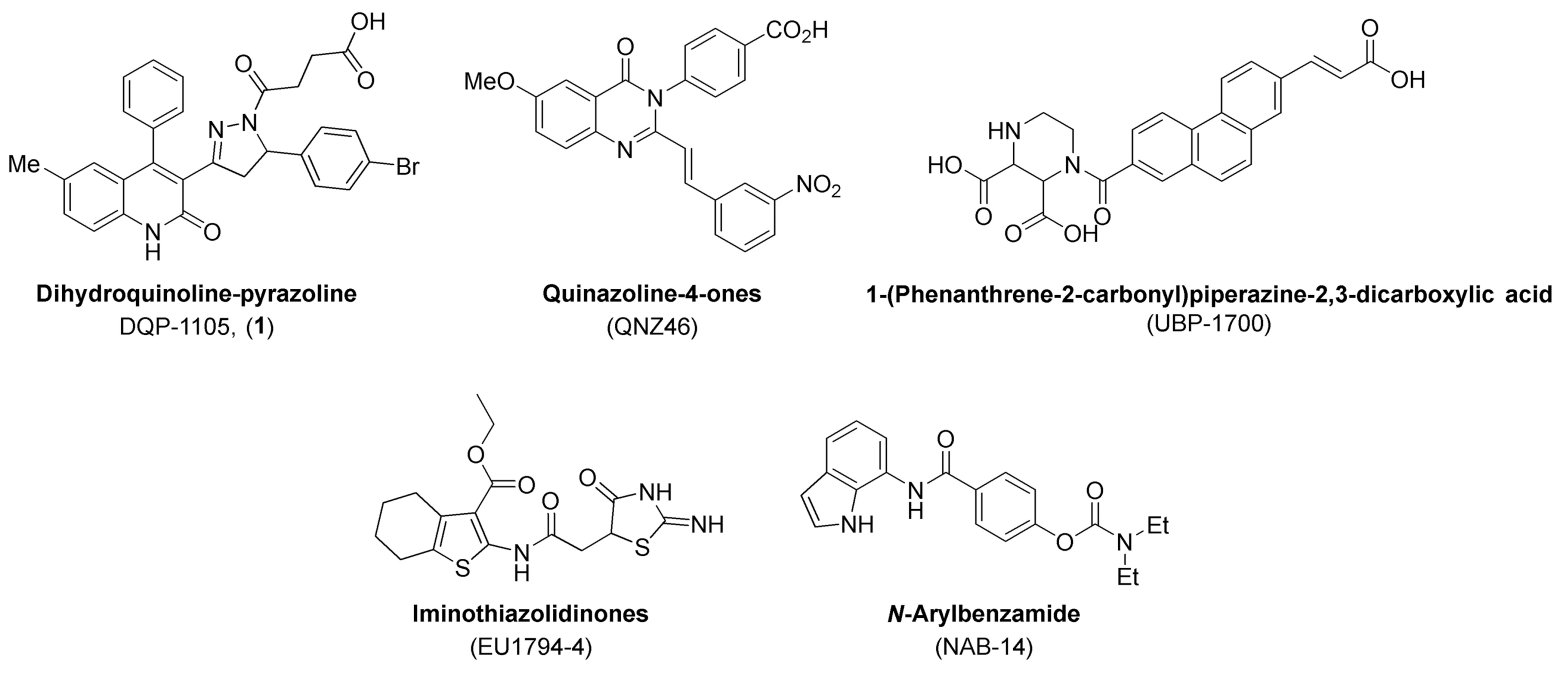
Structures for GluN2C/D-selective NAMs: **DQP-1105** (**1**) (Acker et al.^32^), **QNZ-46** (Mosley et al.;^34^ Hansen and Traynelis^42^), **UBP-1700** (Wang et al.^38^), **EU1794**-**4** (Katzman et al.;^35^ Perszyk et al.,^36^^37^), and **NAB-14** (Swanger et al.^48^).

**Figure 3:**
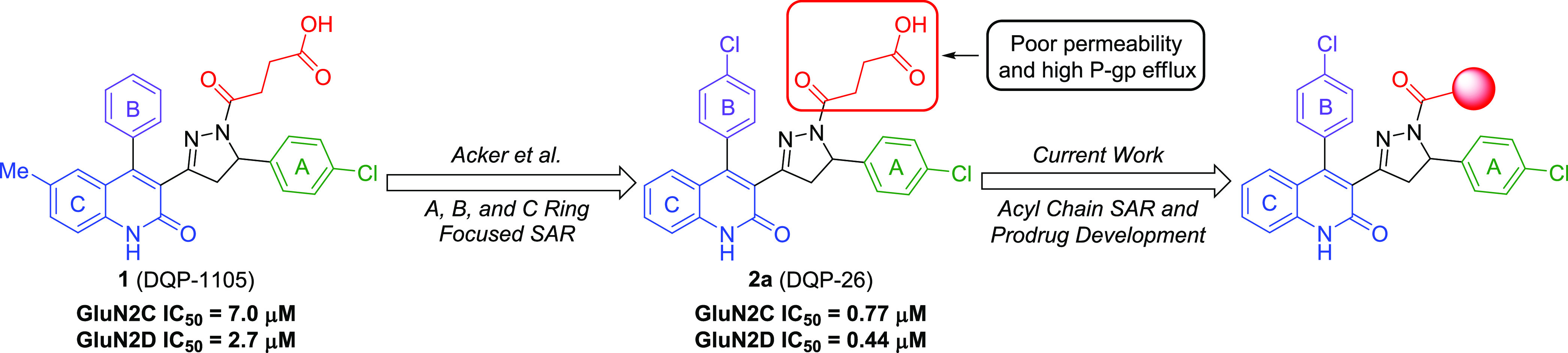
SAR studies conducted on DQP-based GluN2C/D NAMs.

The most promising discoveries have emerged from NMDAR allosteric modulators,^4,8,10,11,13^ which can provide unique advantages over other drug classes (e.g., channel blockers, agonists, and competitive antagonists). These advantages include enhanced subtype selectivity, binding modes that do not interfere with highly conserved ligand-binding domains or the primary channel pore, and the ability to preserve certain levels of receptor function for modulator-bound receptors, thereby avoiding complete receptor blockade. These molecules can either amplify (positive allosteric modulators, PAMs) or inhibit (negative allosteric modulators, NAMs) NMDAR activity through a variety of mechanisms.^4,10−13^ In terms of subunit-selective NAMs, compounds that inhibit GluN2A- (prototype TCN-201)^22−29^ and GluN2B-containing NMDARs (prototype ifenprodil)^8,30,31^ have been well-described. However, while the number of reported scaffolds with GluN2C/D-selective NAM activity has steadily increased over the years^32−38^ (Figure 2), drug development and pharmacological characterization efforts on such compounds are lacking. Furthermore, the mechanism of action of existing GluN2C/D-selective NAMs is not well understood due to lack of structural data for their binding sites, although recent description of GluN1/GluN2C and GluN1/GluN2D receptor structures may provide a means to explore NAM binding.^39^ Nevertheless, the dihydroquinoline–pyrazoline (DQP) derivative **DQP-1105** (**1**) was identified as an NMDAR NAM with selectivity for GluN2C/D- over GluN2A/B-containing NMDA receptors (Figures 1–3,3) during a high-throughput screen.^40^**DQP-1105** demonstrated noncompetitive and voltage-independent antagonism of NMDARs and inhibited GluN2C- and GluN2D-containing receptors with IC_50_ values of 7.0 and 2.7 μM, respectively.^32^ Additionally, in a murine model of TSC-induced epilepsy (i.e., heterozygote *Tsc1*^*±*^ mice), intraperitoneal (IP)-injected **DQP-1105** (28 mg/kg) diminished seizure burden.^41^ Since GluN2C-containing NMDARs are upregulated in this model of epilepsy, these data provided evidence for the potential utility of GluN2C/D-selective NAMs.

We previously reported the results of an SAR study of **DQP-1105**, whereby compound **2a** (compound **26** in Acker et al.^33^ or **DQP-26**) emerged as the most potent analogue with an IC_50_ of 0.77 μM for GluN2C and 0.44 μM for GluN2D, and 28- and 48-fold subunit selectivity for GluN2D over GluN2A and GluN2B, respectively.^33^ In a screen against relevant ion channels, **2a** exhibited minimal off-target effects. Moreover, **2a** displayed high aqueous solubility and stability in human, rat, and mouse plasma, as well as human liver microsomes (HLMs). However, **2a** suffers from poor cell-membrane permeability (reported *P*_app_ = 0.47 × 10^–6^ cm/s) and high P-glycoprotein (P-gp)-mediated efflux ratio (ER = 55) as determined in a permeability assay.^33^ While initial SAR studies evaluated the A, B, and C aryl rings and identified the succinate arm of **2a** as a key pharmacophore for NMDAR NAM activity, further investigation is necessary to enhance potency, increase subunit selectivity, and improve the pharmacokinetic profile. Herein, we describe efforts to improve the potency and selectivity of **2a** through further optimization of the succinate side chain. We also evaluate prodrug strategies that mask the terminal carboxylic acid moiety as a means to improve the pharmacokinetic properties (Figure 3).

## Results and Discussion

### DQP Inhibitor and Prodrug Design

During the development of **2a**, we observed that replacement of the carboxylic acid of the succinate side chain with alcohol or fluoroalkyl derivatives attenuated permeability and P-gp efflux in the Madin–Darby canine kidney cell line harboring the human P-gp efflux protein (MDCK-MDR1), albeit with reduced potency.^32^ Initial analogue design focused on the acyl chain of **2a** (Figure 3). Toward this goal, the succinate moiety was replaced with functional groups or bioisosteres that would neutralize the carboxylate charge or increase scaffold lipophilicity.

### *In Vitro* Evaluation of DQP NMDAR Antagonism

All target compounds were evaluated for NMDAR antagonism using two-electrode voltage-clamp recordings from *Xenopus laevis* oocytes coexpressing recombinant GluN1 and GluN2A-D subunits (Table 1).^32,42,33^ All analogues were assessed as either a racemic (**2b**, **2e**–**2r**) or diastereomeric (**2d**) mixture to simplify purification and streamline *in vitro* analysis. While the glutarate derivative **2b** (GluN2*C*/2D IC_50_ 0.90 and IC_50_ 0.64 μM) had similar potency to **2a** (Table 1), the corresponding glutamate analogue demonstrated reduced GluN2C/D potency **2d** (IC_50_ 2.1 and 1.1 μM) compared to **2a**. Moreover, as illustrated by the significant loss of NMDAR activity observed with nitrile **2e**, the DQP allosteric site necessitated an H-bond donor/acceptor relationship at the carboxylate position. Further support for this functional group specificity was provided by tetrazole **2h** (GluN2C/D IC_50_ = 0.57 and 0.52 μM), which had comparable potency to **2a**. By performing a fluorine exchange on **2a**, we identified the difluorosuccinate **2i** as our most potent and selective GluN2C/GluN2D NAM (GluN2C/GluN2D IC_50_ 0.35 and 0.13 μM), which demonstrated a 43-fold increased potency for GluN2D over GluN2A and 123-fold increased potency for GluN2D over GluN2B, an improvement in selectivity compared to compound **2a**. The loss of NMDAR activity observed with the trifluoro analogue **2j** (Table S1) reinforced the necessity for an H-bond donor/acceptor relationship to maintain allosteric binding and established that fluorine substitution alone is ineffective. This was verified by the NMDAR profile of difluoro amide **2k**, which despite having reduced selectivity against GluN2B-containing receptors exhibited good GluN2C/D potency (GluN2C/D IC_50_ = 0.38 and 0.24 μM). A single methyl substitution of the amide in compound **2q** improved selectivity against GluN2A and had similar potency as **2a**, whereas dual methyl substitution reduced potency (**2r**). We expect that this is likely due to the ability of the ketone of the amide to accept hydrogen bonds while the amide can readily donate a proton. Due to its superior potency, **2i** was chosen as the lead compound for further mechanistic studies.

**Table 1:**
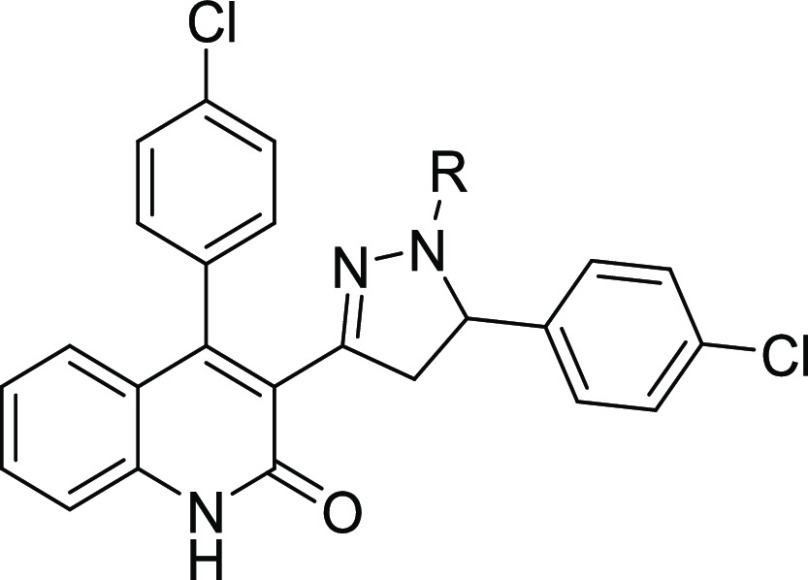

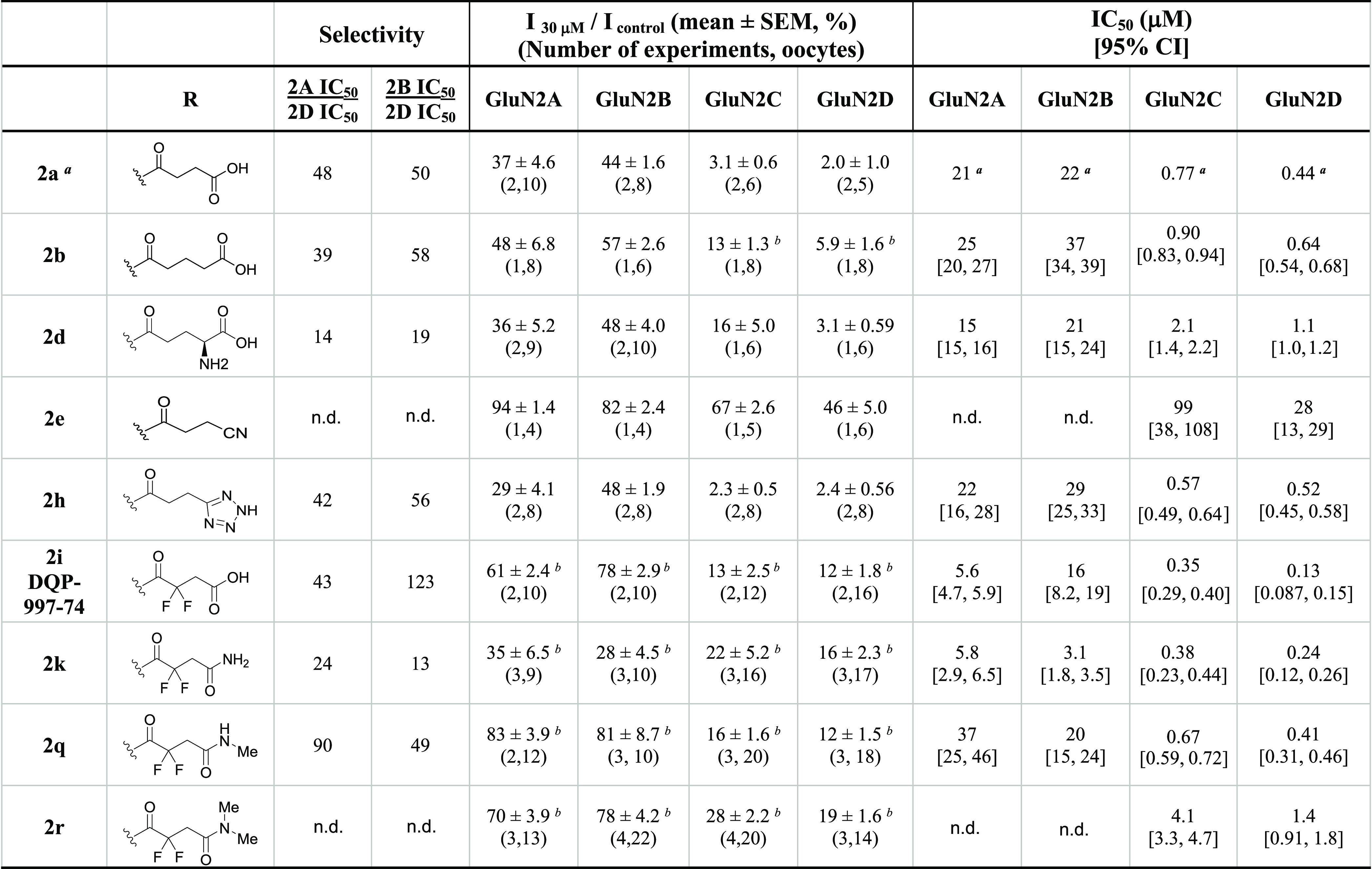
*In Vitro* NMDAR Activity and Selectivity Profiles for DQP Analogues^a^

a Data for compound **2a** were reproduced from Acker et al.^33^ and included for comparison.

b Compounds **2i** (**DQP-997**-**74**) and **2k** were tested at 3 μM due to solubility limits; compounds **2b**, **2q**, and **2r** were tested at 10 μM due either to high potency or solubility limits.

c The mean ratio (±SEM) of the current response to a maximally effective concentration for glutamate (100 μM) and glycine (30 μM) in the presence and absence of a 30 μM test compound is given (*n* = 4–22 oocytes from 1 to 3 independent experiments). Fitted IC_50_ values for inhibition of responses to glutamate (100 μM) and glycine (30 μM) are shown to two significant figures when inhibition at 30 μM resulted in a response less than 70% of control. For some fits, the minimum was fixed to 0% and response in the absence of the test compound was assumed to be 100%. For compounds showing minimal inhibition at the highest concentration (e.g., compound **2i** at GluN2A and GluN2B), the Hill slope was fixed to 1. Values in brackets are 95% confidence intervals determined from the log(IC_50_). n.d. indicates that the IC_50_ and subunit selectivity was not determined.

### Glutamate-Dependent Mechanism of Action

**Figure 4:**
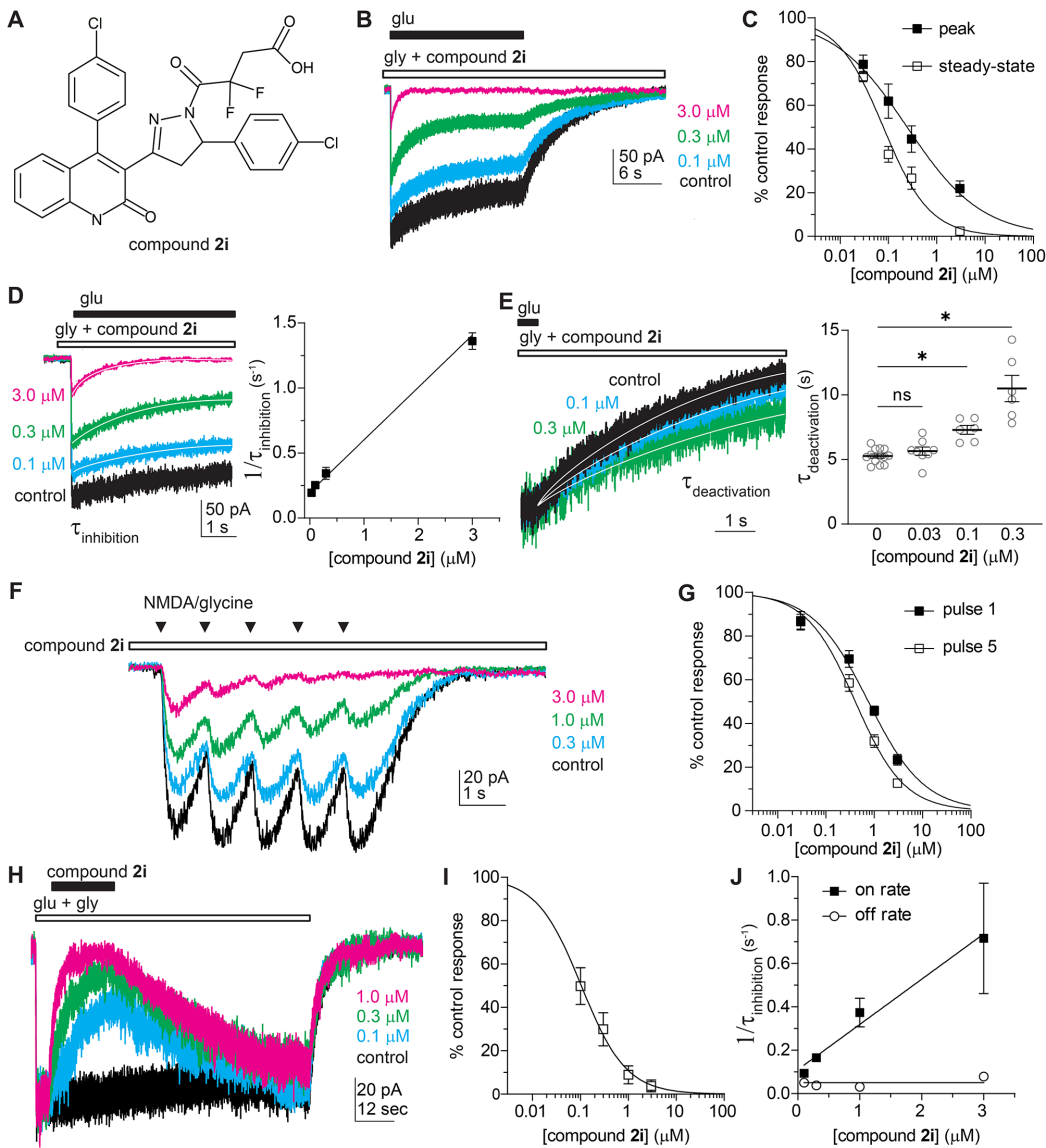
Agonist dependence of compound **2i** (**DQP-997**-**74**). (A) Structure of compound **2i**. (B) Whole-cell current recordings from HEK cells show GluN1/GluN2D responses to glutamate with preapplied compound **2i** or control (vehicle, 0.1% DMSO). (C) Concentration–response curves show peak and steady-state responses normalized to control. (D) The current responses from panel (B) were expanded to show the concentration-dependent time course of relaxation following receptor activation during compound **2i** preapplication. The plot shows a linear relationship between 1/τ_inhibition_ and compound **2i** concentration. (E) The deactivation period following glutamate removal was expanded to show the slowing of receptor deactivation (τ_deactivation_) by compound **2i**. τ_deactivation_ for individual cells and the mean ± SEM are plotted, and data were compared by the mixed effect model, *F*(1.386, 13.40) = 27.45, *p* < 0.001. Asterisks indicate statistically significant differences, see the Results and Discussion section for *p*-values. (F) Whole-cell current recording of responses to 5 brief pressure-applied NMDA (500 μM) and glycine (250 μM) pulses at 1 s intervals. The responses in the absence or increase of concentrations of compound **2i** are superimposed. (G) Concentration–response curves show current responses for the first and last pulse normalized to control. (H) Whole-cell current recordings from HEK cells show GluN1/GluN2D responses to glutamate with coapplication of compound **2i** or control with glutamate. The mean fitted dissociation rate of compound **2i** for all concentrations was 26.5 s (95% confidence interval; 17.4, 35.6; *N* = 15). (I) Plots of the concentration–response curves show the steady-state inhibition response compared to control. (J) The plot shows a linear relationship between a 1/τ_inhibition_ and compound **2i** concentration and a concentration-independent dissociation rate for **2i**.

We previously described the actions of the GluN2C/GluN2D-preferring negative allosteric modulator **DQP-1105** as dependent on glutamate (but not glycine) binding, in that **DQP-1105** affinity increases after GluN1/GluN2D NMDARs bind glutamate.^32^ This phenomenon was revealed by comparing the NMDAR response time courses in cells incubated with **DQP-1105** prior to agonist application to cells that had **DQP-1105** coapplied with the agonist. In both cases, agonist-evoked GluN1/GluN2D receptor current responses exhibited an initial peak response, followed by a concentration-dependent relaxation to a steady-state level of inhibition, indicating that **DQP-1105** affinity increased after glutamate application (Figure S1^32^). Preincubation of the inhibitor reduced the peak response because **DQP-1105** can bind with lower affinity in the absence of an agonist, and this leads to some reduction in the peak. The concentration-dependent relaxation is indicative of an agonist-induced increase in potency that leads to re-equilibration of the inhibitor after agonist binding induces an increase in inhibitor affinity. We investigated the glutamate dependence of compound **2i** (GluN1/GluN2D, Figure 4; GluN1/GluN2C, Figure S2), **DQP-1105** (Figure S1), and compound (*S*)-**2a** (**DQP-69**;^33^Figure S3) using the same protocols.

We preincubated cells with glycine and the inhibitor **2i** (**DQP-997**-**74**) and compared inhibitor potency during the peak and steady-state current responses to glutamate application. Preincubation of the inhibitor produced a reduction in the peak current, suggesting that **2i** could bind to receptors in the absence of glutamate to inhibit the peak current the moment glutamate binds and the channel is activated. The IC_50_ for compound **2i** was 3-fold higher during the peak current responses (IC_50_ 0.23 μM) compared to steady-state responses (IC_50_ 0.08; Figure 4B,C; Table 2), suggesting increased affinity for **2i** following glutamate binding, which allowed **2i** to re-equilibrate with the NMDAR producing a time-dependent inhibition that reflected the time course of inhibitor binding. To investigate the time course of **2i** inhibition, we fitted the relaxation of the current response with a single exponential function. If the current response time course reflects **2i** binding, then the reciprocal of τ_inhibition_ should be linearly related to its concentration, with the slope equal to *k*_ON_ and intercept equal to *k*_OFF_. The exponential time course of the current response was correlated with compound **2i** concentration (*R*^2^ = 0.82), and linear regression analysis yielded values of 4.0 × 10^5^ M^–1^ s^–1^ for *k*_ON_ and 0.17 s^–1^ for *k*_OFF_ (Figure 4D), suggesting a *K*_D_ value of 0.43 μM. The deactivation of GluN1/GluN2D receptors (5.3 ± 0.2 s) was slowed by the presence of compound **2i** when applied at concentrations of 0.1 μM (7.3 ± 0.3 s; *p* = 0.017) and 0.3 μM (10.5 ± 1.0 s; *p* = 0.002) but not at 0.03 μM (5.6 ± 0.3 s; *p* = 0.518; Figure 4E). These data suggest that glutamate unbinding was slowed in the presence of **2i**. This phenomenon was previously discovered for QNZ-46, a glutamate-dependent GluN2*C*/2D-selective inhibitor with a different structural core.^42^ As proposed in that study, the prolonged deactivation may indicate that **2i** slows a conformational change necessary for glutamate unbinding or **2i** may need to unbind before glutamate can unbind.

**Table 2:** Agonist Dependence of DQP Analogues on GluN1/GluN2D NMDA Receptors^a^

	IC_50_, μM (95% CI)		
	**1** (DQP-1105)	(***S***)**-2a**	**2i** (DQP**-997-74**)
peak	1.4 (1.1, 1.7)	2.1 (1.6, 3.2)	0.23 (0.16, 0.32)
steady-state	0.72 (0.61, 0.84)	0.51 (0.39, 0.63)	0.08 (0.07, 0.09)
*N*	9	5	8
peak vs steady-state	*F*(1, 70) = 27, *p* < 0.001	*F*(1, 49) = 73, *p* < 0.001	*F*(1, 71) = 43, *p* < 0.001
pulse 1	4.9 (3.7, 6.5)	1.8 (1.3, 2.6)	0.75 (0.60, 0.94)
pulse 5	1.6 (1.3, 2.1)	0.75 (0.60, 0.94)	0.40 (0.32, 0.51)
*N*	7	8	8
pulse 1 vs pulse 5	*F*(1, 54) = 36, *p* < 0.001	*F*(1, 58) = 34, *p* < 0.001	*F*(1, 60) = 16, *p* = 0.002

a *N* represents the number of cells. IC_50_ value comparisons were made by extra sum-of-squares *F* tests.

We also evaluated the mechanism of inhibition of GluN1/GluN2C expressed in HEK cells preincubated with glycine for inhibitor **2i** (**DQP-997**-**74**) by comparing inhibitor potency during the peak and steady-state current responses to glutamate application. The IC_50_ for compound **2i** on GluN1/GluN2C receptors was higher during the peak current responses (IC_50_ 5.0 μM, 95% confidence interval 1.4–8.5 μM) compared to steady-state responses (IC_50_ 0.25 μM, 95% confidence interval 0.15–0.35 μM; *F*(1, 50) = 81.34, *p* < 0.0001; see Figure S2), suggesting increased affinity for **2i** at glutamate-bound GluN1/GluN2C receptors compared to NMDARs without glutamate bound. The exponential time course of the current response was correlated with compound **2i** concentration (*R*^2^ = 0.79), and linear regression analysis yielded values of 8.0 × 10^5^ M^–1^ s^–1^ for *k*_ON_ and 0.27 s^–1^ for *k*_OFF_ (Figure 4D), suggesting a *K*_D_ value of 0.33 μM. The deactivation of GluN1/GluN2C receptors (0.53 ± 0.07 s) was slowed by the presence of compound **2i** when applied at concentrations of 1 μM (1.3 ± 0.14 s; *p* = 0.001) and 3 μM (2.3 ± 0.40 s; *p* < 0.0001) but not at 0.1 μM (0.61 ± 0.07 s; *p* = 0.965) and 0.3 μM (0.88 ± 0.13 s; *p* = 0.115; Figure S2).

In addition, we evaluated how glutamate dependence affects inhibition of responses to brief agonist applications similar to phasic glutamate release that occurs at synapses. To do so, we preincubated cells expressing GluN1/GluN2D receptors with the inhibitor **2i** and then applied trains of five brief pulses of NMDA (which dissociates more rapidly than glutamate) and glycine from a pressurized micropipette to mimic repetitive synaptic transmission (Figure 4F). We measured the peak amplitude and compared the concentration–response curves for the inhibition of the first and last response (Figure 4G). The initial train of pulses in the absence of drug controls for systematic errors such as internalization of receptors. During trains of brief agonist applications, the IC_50_ of compound **2i** was 1.9-fold higher at the first pulse (IC_50_ = 0.75 μM) compared to the fifth pulse (IC_50_ = 0.40 μM; Figure 4F,G), which suggests that inhibition may be greater during prolonged or repetitive receptor activation.

To further evaluate whether **2i** followed the law of mass action, we coapplied different concentrations of **2i** with glutamate during the steady-state glutamate and glycine responses of GluN1/GluN2D receptors expressed in HEK cells (Figure 4H). We measured the steady-state inhibition produced by each concentration of **2i** tested and determined that the IC_50_ for compound **2i** under these conditions was 0.10 μM (Figure 4I, Hill slope 0.93), similar to that found at the steady state during preapplication (Figure 4B). We also fitted the onset and offset time course of the inhibition produced by compound **2i** with a single exponential function (Figure 4J). The exponential time course of the current response was correlated with compound **2i** concentration (*R*^2^ = 0.51), and linear regression analysis yielded values of 2.1 × 10^5^ M^–1^ s^–1^ for *k*_ON_ and 0.05 s^–1^ for *k*_OFF_, suggesting a *K*_D_ value of 0.24 μM. The time course of recovery from **2i** inhibition was independent of concentration as expected for drug binding to a single site and suggests that inhibition by **2i** does not involve enhanced desensitization.

These data together with similar analysis of **DQP-1105** (Figure S1) and compound **(*****S*****)-2a** (**DQP-69**;^33^Figure S3) suggest that DQP compounds have varying levels of glutamate dependence. A comparison of the fold-change produced by the inhibitor in IC_50_ for the peak and steady-state GluN1/GluN2D current responses to glutamate showed a significant difference between **DQP-1105** (2.1 ± 0.2) and (*S*)-**2a** (3.9 ± 0.3; *p =* 0.005), whereas no statistical differences were found for **DQP-1105** and compound **2i** (3.0 ± 0.3, *p* = 0.078) or between (*S*)-**2a** and compound **2i** (*p =* 0.174; ANOVA, *F*(2, 14) = 14.29, *p* = 0.002). Compound **2i** appeared to show a greater difference at GluN1/GluN2C (Figure S2). These findings demonstrate that both potency and glutamate dependence were modified by structural modifications of the DQP scaffold and are dependent on the GluN2 subunit. Moreover, the data support the idea that DQP analogues can inhibit at a lower affinity in the absence of glutamate compared to when glutamate is present. The possibility that DQP analogues promote a time-dependent desensitization of normally non-desensitizing GluN2D-containing NMDARs is not supported by the concentration dependence of the relaxation time course and concentration independence of the time course for recovery from inhibition.

### *In Vivo* Pharmacokinetic Evaluation of **2i** and **2k**

**Figure 5:**
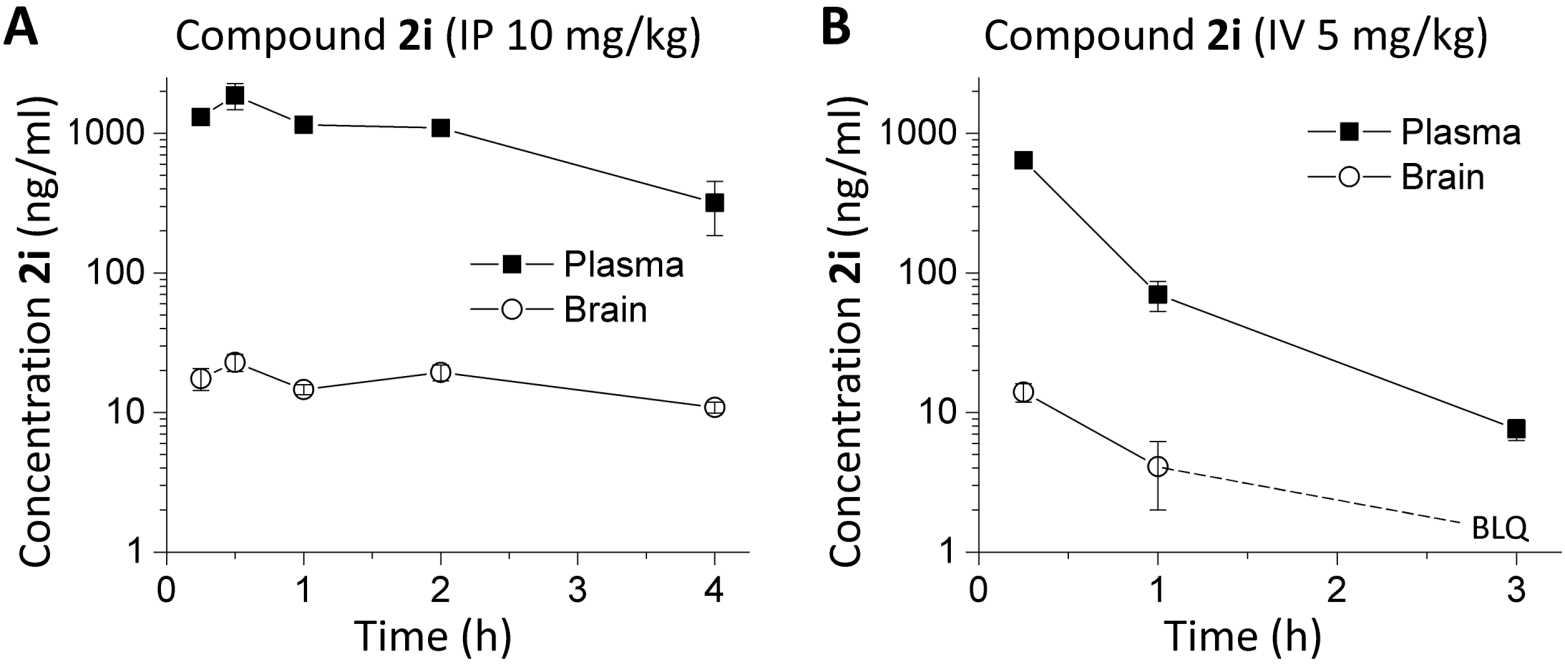
Mouse plasma and brain pharmacokinetic profiles of **2i** (**DQP-997**-**74**). C57Bl/6 mice were administered (A) 10 mg/kg of **2i** IP using 50:50 PEG400/H_2_O as a vehicle or (B) 5 mg/kg **2i** IV in 5% *N*-methyl-2-pyrrolidine, 5% Solutol HS-15, and 90% saline. The concentration of **2i** was followed in the plasma and brain. Data are the mean concentration ± SEM at each time point; SEM shown when larger than symbol. BLQ indicated below the level of quantification.

In order to determine whether **2i** (**DQP-997**-**74**) and the uncharged amide analogue **2k** could be used to probe GluN2C and GluN2D actions *in vivo*, we performed a mouse pharmacokinetic study and measured the exposure of **2i** in plasma and brain tissue (Figure 5). The concentration of **2i** observed in the brain remained low with a Cmax of only 23 ng/g after a 10 mg/kg intraperitoneal (IP) injection. When injected intravenously (IV) **2i** exhibited even lower Cmax within the brain (14 ng/g at 15 min) and plasma (642 ng/mL at 15 min) that rapidly diminished over time. However, **2i** plasma and brain concentrations of **2i** remained relatively stable for at least 2 h following IP administration. Compound **2k** replaces the carboxylate with an amide, which will be uncharged at physiological pH. Compound **2k** showed a similar profile, with modestly lower plasma and brain levels (Table 3), which indicated that removal of the charged carboxylate did not improve the brain permeability. Although mouse brain Cmax levels for **2i** were low following IP administration, they were maintained over the course of 4 h, and the compound could reach brain concentrations (40 nM in brain compartment, upper limit of 200 nM if **2i** is restricted to extracellular space) that might provide some NMDAR antagonism (GluN2D IC_50_ 130 nM), although the free fraction of **2i** in brain (<0.1%) was not improved compared to **2a** (Table S2).

**Table 3:** Pharmacokinetic Analysis of GluN2C and GluN2D Negative Allosteric Modulators Following IP Administration^a^

	compound **2i**		compound **2k**	
	plasma	brain	plasma	brain
*C*_max_ (ng/mL)	1873	23	815	13
*T*_max_ (h)	0.5	0.5	0.5	4
*t*_1/2_ (h)	1.5	>4	4.9	n.d.
AUC_0.25–4 h_ (h·ng/mL)	3854	64	2270	41

a Plasma and brain levels of **2i** (**DQP-997**-**74**) and **2k** were quantified using LC-MS/MS following 10 mg/kg IP administration. n.d., not determined.

### Assessment of DQP **2i** against Seizures in *Tsc1*^*±*^ Mice

**Figure 6:**
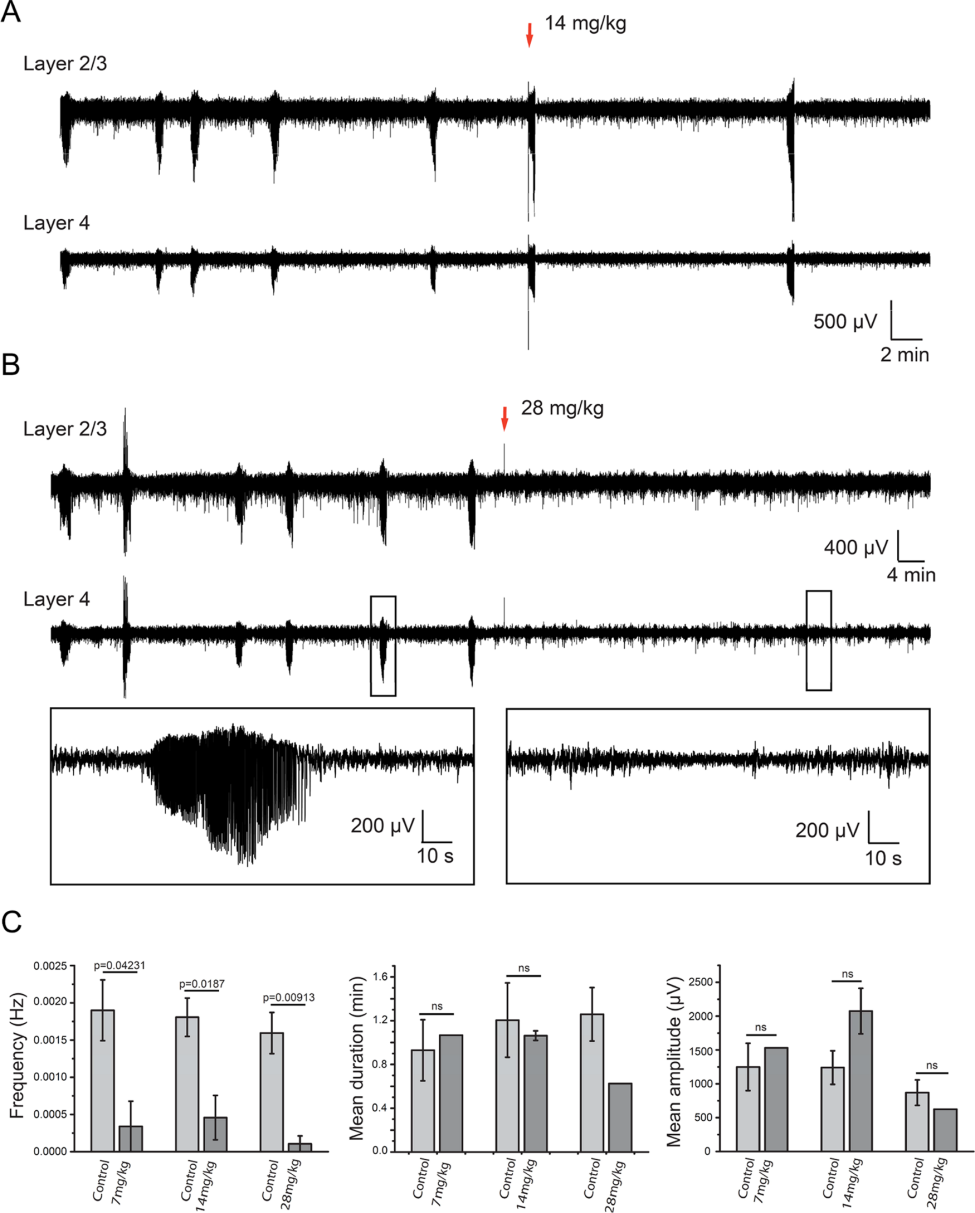
Antiepileptic effect of IP administration of **2i** (**DQP-997**-**74**) *in vivo*. (A) Representative intracortical EEG recordings of spontaneous electrographic seizures in layer 2/3 (top) and layer 4 (bottom) in a head-restrained P14 *Tsc1*^±^ mouse. Recordings were performed prior to the administration of 14 mg/kg of **2i** as indicated by the red arrow. (B) Representative intracortical EEG recordings of spontaneous seizures in layer 2/3 (top) and layer 4 in head-restrained P14 *Tsc1*^±^ mouse. The administration of 28 mg/kg of **2i** is indicated by the red arrow. Note the diminution of the seizure frequency after IP injection. Black boxes show extended traces of a seizure recorded before (left) and a seizure-free trace recorded after the injection of **2i** (right). (C) Mean and SEM of seizure frequency, duration, and amplitude over the 2 h EEG recording before IP administration of 7 mg/kg (*n* = 3), 14 mg/kg (*n* = 4), and 28 mg/kg (*n* = 5) of **2i** and the 2–2.5 h of EEG recording postadministration.

The acute effects of **2i** on *in vivo* epileptic seizures in male *Tsc1*^*±*^ mice were investigated after IP administration at three different doses (7, 14, and 28 mg/kg) during electroencephalogram (EEG) recordings. *Tsc1*^*±*^ mice appear to have upregulated expression of GluN2C.^41^ Administration of **2i** at 14 mg/kg significantly reduced spontaneous electrographic seizures in neocortical layers 2/3 and 4 (Figure 6A). In a group of four mice tested at this **2i** dose, the seizures were completely stopped in two mice after injection, while the remaining mice were seizure-free after ∼70 min. Furthermore, upon increasing the dose to 28 mg/kg, epileptic events stopped immediately with considerable declines in seizure frequency, duration, and amplitude compared to those of control subjects (Figure 6B,C). In a group of five *Tsc1*^*±*^ mice, the 28 mg/kg dose of **2i** completely stopped seizure events in four subjects, while the last mouse demonstrated observable seizure reductions after ∼50 min. This *in vivo* antiseizure activity illustrates the therapeutic potential of compounds within this class for GluN2C/D-associated epilepsy.

### *In Vivo* Pharmacokinetic Profile of DQP Inhibitors and Prodrugs

#### *In Vitro* DMPK Profiles for DQP Prodrugs

Anticipating that the carboxylate side chain may hinder BBB penetrance, we synthesized and biologically screened a series of **2i** prodrugs with ester and amide masking groups. With the classical physiochemical parameters required for brain exposure (i.e., molecular weight, MW, lipophilicity, and topological polar surface area, TPSA) and drug release mechanisms beyond the BBB in mind, we focused on simple alkyl-based promoieties. Our selected lead compound (**2i**), a close analogue (**2a**), and their corresponding prodrugs (**2l**–**r**) were studied *in vitro* to ascertain drug solubility, metabolic stability in liver microsomes (LMs), plasma stability, mouse brain homogenate stability, and cytochrome P450 (CYP450) inhibition (Tables S3 and S4 for predicted absorption, distribution, metabolism, and excretion or ADME parameters). We first determined the solubility of compounds **2a**, **2i**, and **2k**–**2r** in phosphate-buffered saline (PBS, 1% DMSO) at room temperature (Table 4 and Figure S4). Our previous lead **2a** demonstrated a relatively high PBS solubility of ∼208 μM. However, we observed a reduction in the solubility of difluoro derivatives **2i** (∼38 μM) and **2k** (∼30 μM). An accurate solubility comparison via a nephelometer between the active parent species (**2i** and **2k**) and their analogous prodrugs (**2l**–**2r**) could not be obtained. However, since all compounds precipitated within the range 25–55 μM, they likely possess aqueous solubility values similar to those of the parent.

**Table 4:**
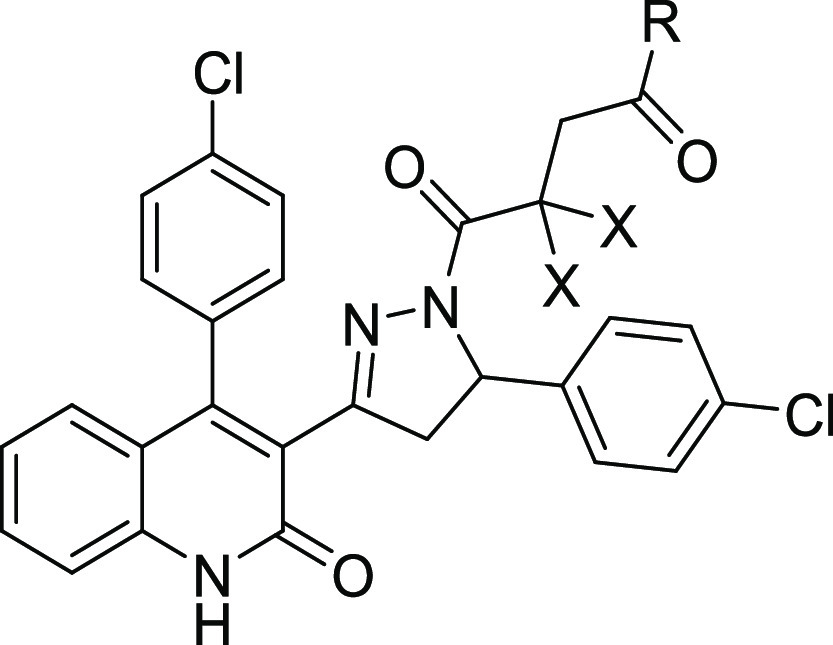
*In Vitro* Solubility and Stability in Plasma, Liver Microsomes, and Mouse Brain Homogenate^g^

							mouse brain homogenate (C57BL/6)^b^^,^^e^	
	X	R	kinetic solubility in PBS (μM)^a^	liver microsome stability h/m *T*_1/2_ (min)^c^	human/mouse Cl_int_, (μL/min/mg)	plasma stability h/m *T*_1/2_ (min)^d^	% remaining at 4 h^f^	**2i** formation (μM at 4 h)^f^
**2a**	H	OH	208 ± 5.4	>30/>30	<23/<23	>120/>120	94	n.d.
**2i**, DQP-997-74	F	OH	38 ± 7.9	>30/>30	<23/<23	>120/>120	105	n.d.
**2k**	F	NH_2_	30 ± 5.6	>30/>30	<23/<23	>120/>120	44	n.d.
**2l**	F	OEt	43 ± 18	3.6/2.6	193/267	>120/4.2	43	2.7 ± 0.1
**2m**	F	O*i*-Pr	43 ± 10	4.0/2.9	173/239	>120/37	56	1.2 ± 0.2
**2n**	F	OCH_2_C_3_H_5_	27 ± 9.0	2.4/1.8	289/385	>120/1.3	25	2.8 ± 0.2
**2o**	F	ONp	52 ± 6.3	3.7/4.0	187/173	>120/4.0	53	1.8 ± 0.04
**2p**	F	OBn	40 ± 12	6.6/7.2	105/96	>120/1.4	31	3.0 ± 0.08
**2q**	F	NHMe	52 ± 13	6.7/3.9	104/178	>120/>120	33	<0.1
**2r**	F	NMe_2_	43 ± 5.1	4.2/3.0	165/231	>120/>120	93	<0.1

a *n* = 7

b *n* = 2.

c *T*_1/2_ determined from incubation up to 30 min; > 30% of compounds **2a**, **2i** (**DQP-997**-**74**), and **2k** remained at 30 min.

d *T*_1/2_ determined from incubation up to 120 min. h/m indicates values for human and mouse.

e Test compounds were incubated in mouse brain homogenate for 4 h with a starting concentration of 5 μM.

f Relative % remaining and metabolite concentration at 4 h. n.d. indicates not determined.

g Oi-Pr is O-isoproyl, OEt is O-ethyl, ONp is O-neopentyl, and OBn is O-benzyl. Intrinsic clearance (Cl_int_) refers to the enzyme-catalyzed metabolic clearance of a drug which is not influenced by other physiological parameters.

Compounds **2a** and **2i** demonstrated high stability (i.e., greater than 95% remaining over the assay time course) in human and mouse LMs and plasma (Table 4 and Figures S5 and S6). Additionally, **2a** and **2i** either weakly inhibited or displayed no activity against the major drug metabolizing enzymes of CNS clinical relevance, CYP2D6 and CYP3A4 (Table 5 and Figure S4). While amide **2k** was stable in both LMs and human plasma, the compound was metabolized in mouse plasma (42% of prodrug after 2 h) and brain homogenate (56% of prodrug after 4 h; Table 4) and moderately inhibited CYP3A4 (IC_50_ = 2 μM) at concentrations 10-fold higher than the IC_50_ at GluN1/GluN2D (Table 5). Further metabolite identification experiments are required to elucidate which mouse enzymes degraded **2k**. Despite **2k** being stable to human and mouse LMs (greater than 90% of parent remaining after 30 min), **2q** and **2r** decomposed quite rapidly (*t*_1/2_ ≤ 7 min; Table 4). Although there is evidence for **2q** instability in brain homogenate (33% prodrug remaining after 4 h), **2i**, the amidase-catalyzed hydrolysis product, was unobservable and below the limit of quantification (BLQ) by LC-MS/MS (Table 4). Interestingly, **2r** was stable under the same conditions (93% prodrug remaining). Since neither the amide moiety produced **2i** in brain homogenate nor was stable to hepatic metabolism (presumably from *N*-demethylation), we did not advance **2q** or **2r** to *in vivo* pharmacokinetic studies.

**Table 5:**
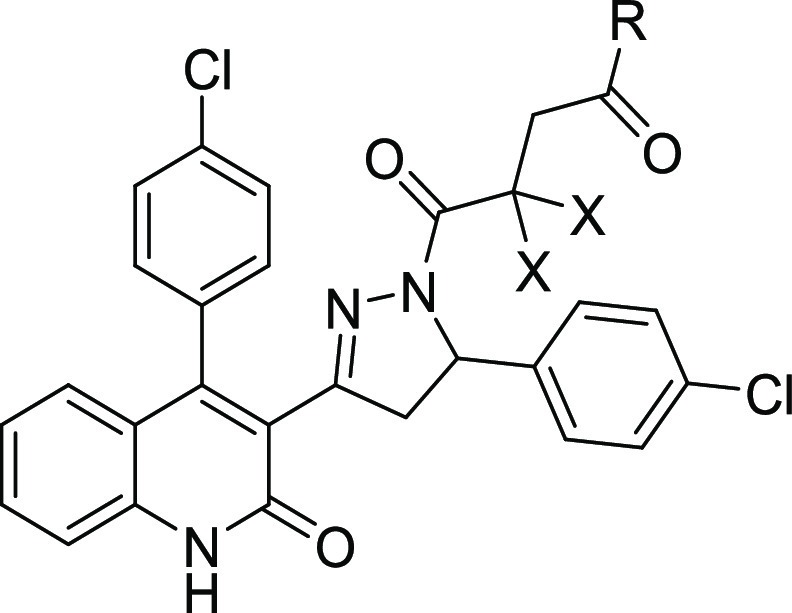
CYP450 Inhibition Profiles for Lead Compounds and Prodrugs

			CYP450 IC_50_ values (μM)^a^	
compound	X	R	CYP2D6	CYP3A4
**2a**	H	OH	18 ± 0.8	17 ± 3.4
**2i**, **DQP-997-74**	F	OH	>20	>20
**2k**	F	NH_2_	>20^b^	2.0 ± 0.4^b^
**2l**	F	OEt	>20	7.8 ± 0.1
**2m**	F	O*i*-Pr	>20	6.3 ± 1.1
**2n**	F	OCH_2_C_3_H_5_	>20	6.7 ± 1.2
**2o**	F	ONp	>20	12 ± 0.04
**2p**	F	OBn	>20	>20
**2q**	F	NHMe	>20	7.7 ± 1.7
**2r**	F	NMe_2_	>20	>20

a *n* = 2.

b *n* = 4/Compounds **2i** (**DQP-997**-**74**), **2l**, **2m**, **2n**, **2o**, and **2p** show some degree of autoactivation of CYP2D6.

All ester prodrugs hydrolyzed to **2i** in mouse brain homogenate, with the less sterically hindered promoieties **2l**, **2n**, and **2p** cleaving more efficiently than the bulkier substrates **2m** and **2o** (Tables 4 and S5). Although stable in human plasma, the ester series also exhibited high metabolic instability in LMs (<5 min) and mouse plasma (<40 min). Additionally, while the ester prodrugs did not inhibit CYP2D6, compounds **2l**–**o** displayed increased CYP3A4 inhibition (6–12 μM) relative to **2i**, which suggests the potential for drug–drug interactions (Table 5).

### *In Vivo* Evaluation of Prodrugs

**Figure 7:**
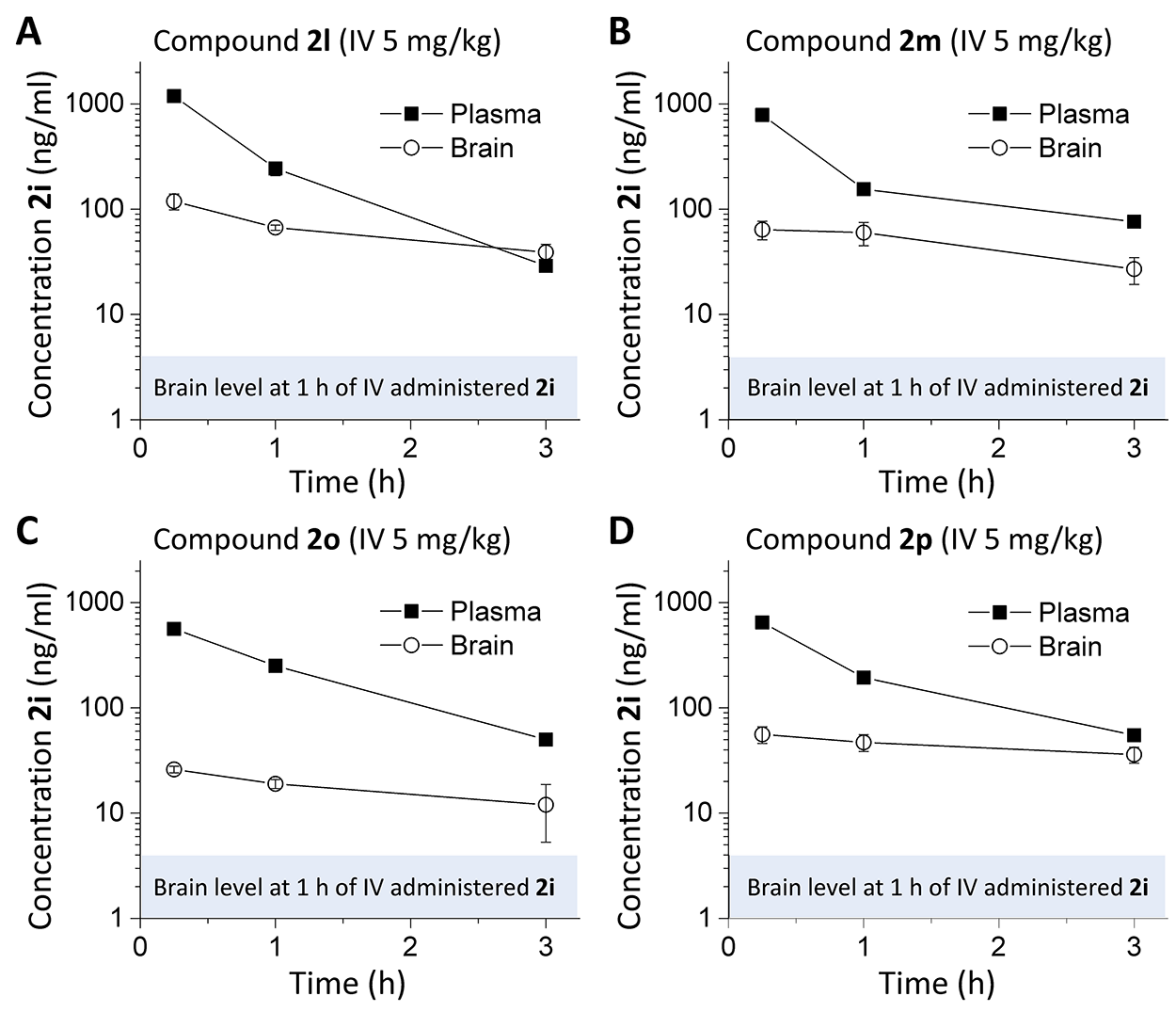
Mouse plasma and brain pharmacokinetic profiles of **2i** (**DQP-997**-**74**) produced by prodrugs. C57Bl/6 mice were administered (A) 5 mg/kg **2l** IV in 5% *N*-methyl-2-pyrrolidine, 5% Solutol HS-15, and 90% saline. (B–D) Concentration of **2i** in plasma and brain following IV administration of prodrugs **2m** (B), **2o** (C), and **2p** (D) (5 mg/kg; same vehicle as (A)). Shaded area is the brain **2i** level 1 h after IV administration, from Figure 5. Data represent the mean concentration ± SEM at each time point; SEM shown when larger than symbol.

We next considered whether a prodrug strategy could improve brain/plasma ratios of compound **2i** by comparing brain and plasma **2i** concentrations following the IV administration of prodrugs or parent compound **2i**. Although ester prodrugs had low stability in *in vitro* assays, we tested whether pharmacokinetic profiling of prodrugs **2l**, **2m**, **2o**, and **2p** could serve as a proof of concept for future prodrug development. Peak plasma and brain concentrations at 15 min for the parent compound **2i**-administered IV were 642 and 14 ng/mL, which were rapidly reduced. Plasma levels of **2i** dropped by ∼10-fold at 1 h, with brain levels BLQ at 3 h (Figure 5B). Several prodrugs increased the parent compound **2i** concentration and residence time within the brain (Figure 7). Prodrugs showed peak plasma and brain levels of 1190/119 ng/mL (compound **2l**), 786/64 ng/mL (compound **2m**), 563/26 ng/mL (compound **2o**), and 647/56 ng/mL (compound **2p**). All prodrugs showed higher **2i** brain levels following IV administration compared with brain levels following IV administration of parent **2i** alone. At 1 h postadministration, **2i** brain levels were 5–10-fold higher for prodrugs than parent **2i** alone. At 3 h, brain levels of **2i**-administered IV were not detectable, whereas three of the prodrugs had a reasonable brain/plasma ratio at 3 h, with *K*_p_ values of 1.36 (**2l**), 0.36 (**2m**), and 0.64 (**2p**), suggesting that prodrugs are capable of higher exposures than **2i** and for a longer duration. The increase in the **2i** brain/plasma ratio was due to the increased amount of **2i** in the brain and longer residence in the brain following IV administration compared to parent **2i** (Figure 7). Although the brain exposure still remains low following the addition of the two fluorines and an ester to **2a** and micromolar concentrations of these prodrugs produced some inhibition of CYP3A4 (Table 5) or underwent rapid metabolism, these data nevertheless demonstrate that a prodrug approach can enhance brain concentrations. If prodrug groups could be designed that were more stable in plasma and liver microsomes and had improved blood–brain barrier penetration compared to the simple esters explored here, the prodrug strategy might provide more robust tool compounds and a potential clinical lead series.

### Stereochemical Preference for Compound **2i**

Previous studies of this class showed stereoselectivity for the most potent DQP analogue **2a** described in Acker et al.,^33^ with (*S*)-**2a** being the more potent and selective enantiomer. We therefore separated the two enantiomers of **2i** (see the Methods section and Figures S7 and S8), determined their structure and optical rotation (Figures S9 and S10), and assessed the potency for recombinant NMDARs. We found that one of the enantiomers (*S*)-(−)-**2i** (i.e., (*S*)-(−)-**DQP-997**-**74**) was clearly more potent, with an IC_50_ at GluN2C/GluN2D of 0.035 μM and selectivity for GluN2D over GluN2A and GluN2B of >100-fold and >400-fold, respectively. By contrast, the other enantiomer (*R*)-(+)-**2i** had lower potency and produced shallow, incomplete inhibition (Figure S11). Table 6 summarizes the results of this analysis. Based upon these findings, future work should focus on the most active and selective isomer, (*S*)-(−)-**2i**. We evaluated potential off-target actions of (*S*)-(−)-**2i** at 1 μM (30 times EC_50_) and found no activity at AMPA, GABA, glycine, nicotinic, or purinergic P_2X_ receptors (Table S6), suggesting minimal off-target actions.

**Table 6:**
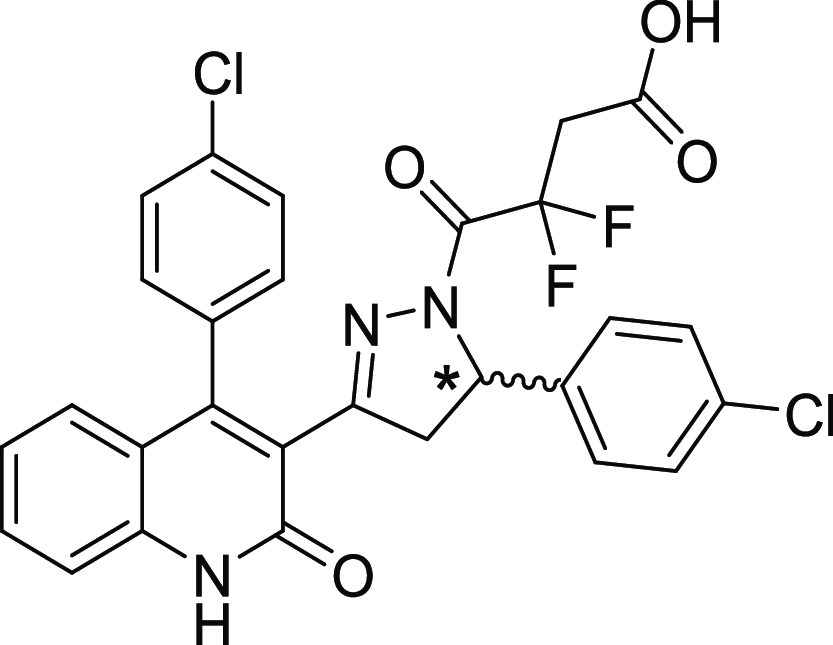
Stereoselectivity of **2i** (**DQP-997**-**74**)^a^

	selectivity		*I*_3 μM_/*I*_control_ (mean ± SEM, %) (number of experiments, oocytes)				IC_50_ (μM) [95% CI]			
	**2A** IC_50_, **2D** IC_50_	**2B** IC_50_, **2D** IC_50_	**GluN2A**	**GluN2B**	**GluN2C**	**GluN2D**	**GluN2A**	**GluN2B**	**GluN2C**	**GluN2D**
racemic **2i**	43	123	61 ± 2.4 (2, 10)	78 ± 2.9 (2, 10)	13 ± 2.5 (2, 12)	12 ± 1.8 (2, 16)	5.6 [4.7, 5.9]	16 [8.2, 19]	0.35 [0.29, 0.40]	0.13 [0.087, 0.15]
**(*****S*****)-(−)-2i**	134	457	67 ± 2.0 (3, 14)	89 ± 6.2 (3, 18)	13 ± 2.6 (3, 16)	3.8 ± 0.60 (4, 25)	4.7 [3.6, 5.6]	16 [8.6, 19]	0.061 [0.054, 0.067]	0.035 [0.027, 0.039]
**(*****R*****)-(+)-2i**	--	--	103 ± 2.5 (3, 16)	99 ± 2.4 (3, 12)	52 ± 2.1 (2, 12)	42 ± 3.5 (2, 13)	--	--	0.51 [0.37, 0.61]	0.31 [0.19, 0.35]

a The mean ratio (±SEM) of the current response to a maximally effective concentration of glutamate (100 μM) and glycine (30 μM) in the presence and absence of a 3 μM test compound is given; numbers of independent experiments and oocytes are given in parentheses. Fitted mean IC_50_ values for inhibition of responses to glutamate (100 μM) and glycine (30 μM) are shown to two significant figures (*n* = 4–15 oocytes from 2 to 3 independent experiments). Values in brackets are 95% confidence intervals determined from the log(IC_50_). Data for compound **2i** shown in Table 1 were included for comparison.

### DQP Analogue and Prodrug Synthesis

**Scheme 1:**
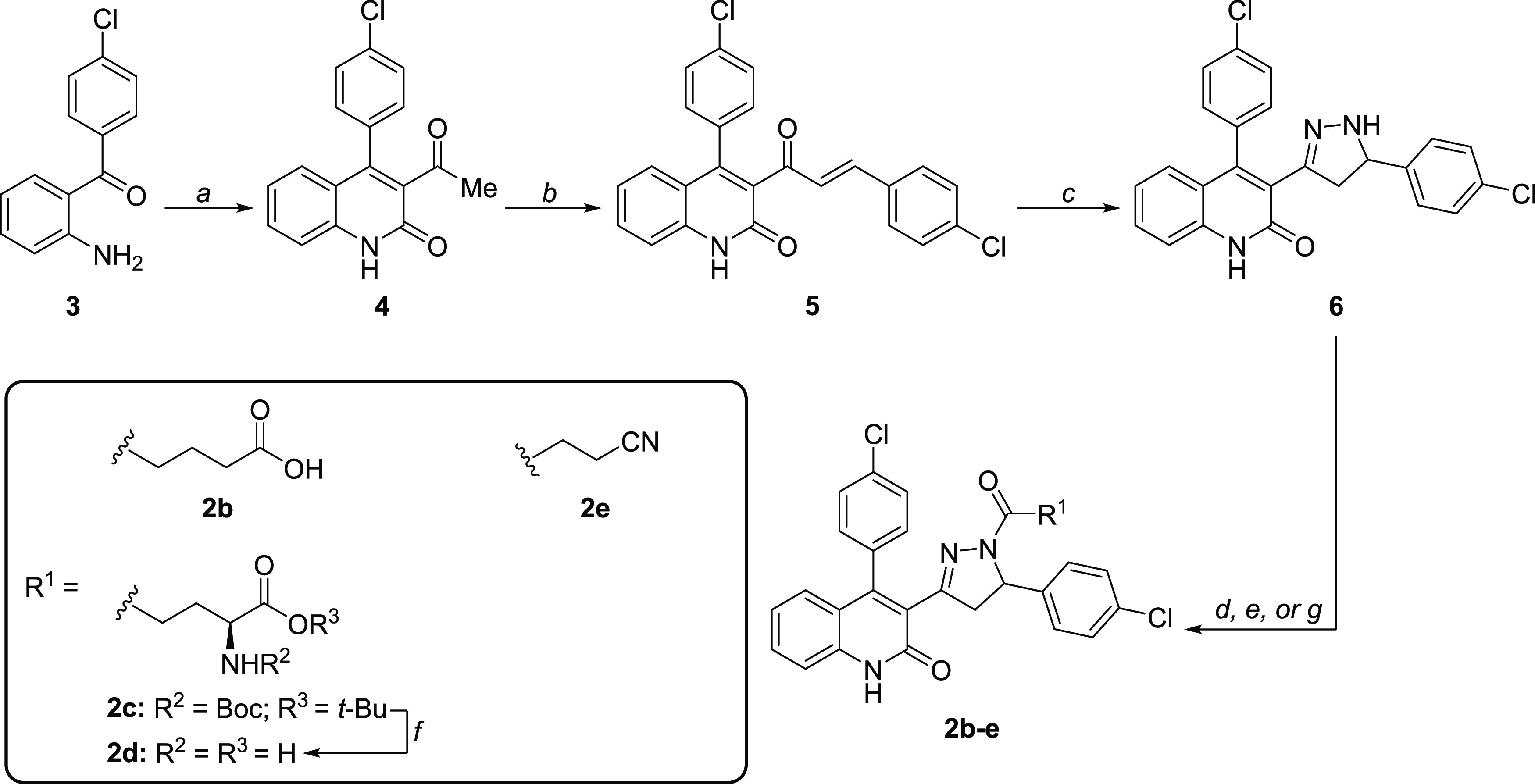
Synthesis of DQP Intermediates and Derivatives with Modified Acyl Side Chains Reagents and conditions: (a) ethyl acetoacetate, 4 Å mol sieves, THF, μwave 160 °C, 30 min, 78%; (b) 4-chlorobenzaldehyde, KOH, 4:3 EtOH/H_2_O, 0 °C to rt, overnight, 93%; (c) 65% H_4_N_2_·H_2_O, EtOH, 110 °C, 2 h, 90%; (d) glutaric anhydride, 4 Å MS, THF, reflux, 5 h, 63%; (e) Boc-Glu-OtBu, HBTU, TEA, DMF, rt, overnight; (f) 1:1 TFA: DCM, 70 °C, μwave, 2 min, 29% over two steps; (g) carboxylic acid, HBTU, TEA, DMF, 85 °C, μwave, 20 min, 45–59%;

**Scheme 2:**
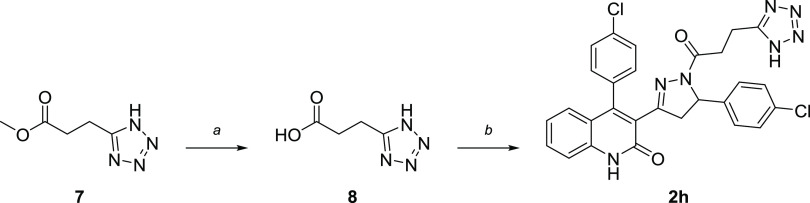
Synthesis of a DQP Analogue with Tetrazole Bioisostere Reagents and conditions: (a) 1 N NaOH, MeOH, rt, 16 h; (b) **6**, Et_3_N, T3P, DMF, 0 °C–rt, 16 h, 64%.

All novel analogues were synthesized from previously reported intermediates, specifically acryloylquinolinone **5** and DQP **6** (Scheme 1).^33,43^ In short, these compounds were generated along a three-step synthesis starting from commercially available 2-aminobenzophenone **3** (Scheme 1). Compound **3** first underwent a sequential Knoevenagel condensation/cyclization with ethyl acetoacetate at 160 °C, followed by aldol condensation with 4-chlorobenzaldehyde in the presence of KOH to form intermediate **5**. Lastly, DQP **6** was obtained after a cyclization reaction between compound **5** and hydrazine monohydrate in EtOH. Utilizing the nucleophilicity of the pyrazoline ring, DQP **6** was acylated with various anhydrides and activated carboxylic acids to generate the analogues used for this study (Scheme 1). For example, the final compound **2b** was synthesized by heating DQP **6** in the presence of glutaric anhydride. Moreover, amide coupling between **6** and protected glutamate or short-chain carboxylic acids was successfully performed with HBTU and TEA to construct **2c** and **2e** (Scheme 1). Glutamate derivative **2c** was further subjected to standard Boc-deprotection conditions in 1:1 TFA/DCM to supply DQP **2d**. Deviating slightly, tetrazole **2h** was obtained through hydrolysis of commercial reagent **7**, followed by an amide coupling procedure using T3P to provide compound **2h** (Scheme 2).

**Scheme 3:**
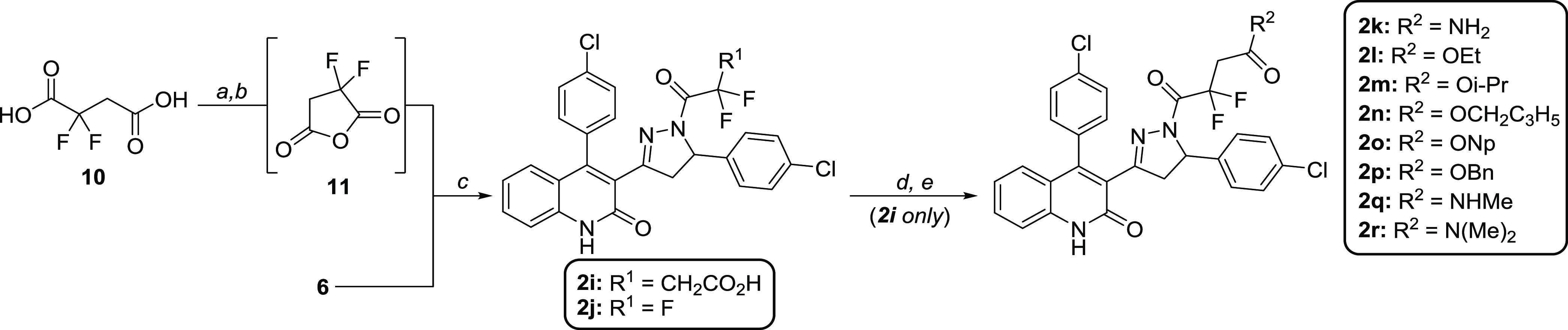
Synthesis of Fluorinated DQP Analogues and Prodrugs Reagents and conditions: (a) TFAA, *i*-PrOAc, rt to 50 °C, 1.5 h; (b) **6**, THF, 0 °C, 1 h, 89% over two steps; (c) TFAA, THF, 4 Å MS, 0 °C to rt, 1 h, 86%; (d) **2g**, oxalyl chloride, DCM, DMF, rt; (e) alcohol or amine, DCM, 0 °C, 41–73% over two steps.

Moving forward, fluorinated derivatives **2i** and **2j** were synthesized in a similar fashion (Scheme 3) to the analogues depicted in Scheme 1. While **2j** simply required acylation of DQP **6** with trifluoroacetic anhydride (TFAA), difluorosuccinate **2i** necessitated slight procedural modification. First, 2,2-difluorosuccinic acid **10** was cyclized with TFAA under mild heating conditions in isopropyl acetate (*i*-PrOAc) to generate the highly reactive cyclic anhydride **11**.^44^ Prior to the reaction with DQP **6**, compound **11** was carefully concentrated to eliminate the remaining TFAA and avoid significant product evaporation. DQP **6** was then slowly added to a cooled solution of **11** in THF to give the final product **2i** (**DQP-997**-**74**) in an 89% yield. When anhydride **11** was added to DQP **6** (i.e., the order of addition was switched), an impurity resulting from acyl chain HF-elimination emerged that was difficult to isolate and remove from the target analogue **2i**. Finally, to obtain amide **2k** and the corresponding prodrugs **2l**–**2r**, compound **2i** was converted into an acyl chloride utilizing *in situ* generation of Vilsmeier reagent, followed by an immediate reaction with the corresponding alcohols or amines (Scheme 3). All base-catalyzed esterifications and amidations performed on **2i** also resulted in the formation of the HF-elimination byproduct (result not shown), and hence, acidic conditions were required for prodrug synthesis.

### Conclusions

We have explored several strategies to improve the properties of the DQP class of GluN2C/D-selective NAMs based on the **2a** scaffold. We identified a difluorosuccinate analogue **2i** (**DQP-997**-**74**), which exhibited a high NMDAR potency (GluN2C IC_50_ 0.35 μM and GluN2D IC_50_ 0.13 μM) and strong subunit selectivity for GluN2D over GluN2A/B compared to the parent **2a**. Previous work with DQP-1105 shows intermediate potency at triheteromeric receptors containing two different GluN2 subunits,^45,46^ and thus we expect compound **2i** to retain effectiveness at triheteroemric receptors but at reduced potency. Due to compound **2i** being agonist-dependent, it is an effective inhibitor of high-frequency neurotransmission that can be prevalent in certain pathological conditions, such as epilepsy. We performed a preliminary ester and amide prodrug screen on **2i** to improve the BBB penetration. Although, the proof-of-concept prodrugs had poor aqueous solubility and metabolic stability profiles in LMs and plasma, they still increased **2i** levels in brain 5–10-fold over parent **2i**-administered IV, suggesting that this strategy could be a beneficial way to improve the utility of this compound class if more stable analogues could be synthesized. Separation of stereoisomers suggested that the *S* enantiomer, (*S*)-(−)-**2i**, which we also refer to as (*S*)-(−)-**DQP-997**-**74**, was the most potent and selective, having a GluN2D IC_50_ of 35 nM and selectivity of over 100-fold against GluN2A and over 400-fold against GluN2B and minimal off-target actions. Given the high potency of (*S*)-(−)-**2i**, exposure levels of **2i** in the brain may be adequate to engage the target. Consistent with this idea, racemic **2i** produced anticonvulsant actions in *Tsc1*^*±*^ mice, which overexpress the GluN2C subunit. These data demonstrate that the DQP series of NAMs has promising therapeutic potential. Future work will entail further prodrug development of (*S*)-(−)-**DQP-997**-**74** to improve brain penetrance.

## Methods

### Chemistry

All reactions were conducted in oven-dried glassware by using distilled and degassed solvents under a positive pressure of dry argon with standard Schlenk techniques unless stated otherwise. All of the commercially available chemicals were used without further purification. All microwave reactions were performed with a Biotage Initiator microwave synthesizer. Analytical thin-layer chromatography (TLC) was carried out on commercially available (MilliporeSigma) aluminum-supported (thickness: 200 μm) or glass (2.5 cm × 7.5 cm) silica gel plates with a fluorescent indicator (F_254_). Visualization of compounds on TLC plates was achieved using UV light (254 nm) and/or ethanolic phosphomolybdic acid (PMA) or aqueous potassium permanganate (KMnO_4_) stains. TLC retention factors (*R*_f_) were calculated as an average of three experimental runs. Automated flash column chromatography was performed using a Teledyne ISCO CombiFlash Companion system with RediSep Rf normal-phase silica-gel-packed or RediSep Rf Gold reversed-phase C18 columns (Teledyne Isco). NMR spectra (^1^H, ^13^C, and ^19^F) were acquired using a Bruker Ascend 600 MHz spectrometer, a Varian INOVA 600 MHz spectrometer, a Varian INOVA 500 MHz spectrometer, a Bruker NEO 400 MHz spectrometer, a Varian INOVA 400 MHz spectrometer, or a Varian VNMR 400 MHz spectrometer (Emory University NMR Center, directed by Dr. Shaoxiong Wu). NMR samples were prepared in deuterated chloroform (CDCl_3_), deuterated methanol (CD_3_OD), or deuterated dimethyl sulfoxide (DMSO-*d*_6_) using tetramethylsilane (TMS) or the corresponding residual solvent peaks (CDCl_3_: ^1^H = 7.26 ppm, ^13^C = 77.16 ppm; CD_3_OD: ^1^H = 3.31 ppm, ^13^C = 49.00 ppm; DMSO-d6: ^1^H = 2.50 ppm, ^13^C = 39.52 ppm; TMS: ^1^H = 0.00 ppm) as internal references. Alternatively, the residual chloroform, methanol, or dimethyl sulfoxide peak in ^1^H NMR was used as an absolute reference for ^31^P and ^19^F NMR, unless otherwise specified. MestreNova software was used to process all of the NMR spectra. NMR data include chemical shifts (δ) reported in ppm, multiplicities indicated as s (singlet), d (doublet), t (triplet), q (quartet), dd (doublet of doublets), dt (doublet of triplets), td (triplet of doublets), m (multiplet), and br (broad), and coupling constants (*J*) reported in Hz. High-resolution mass spectrometry (HRMS) was performed by the Emory University Mass Spectrometry Center using a Thermo Scientific Exactive Plus mass spectrometer, directed by Dr. Fred Strobel. Liquid chromatography–mass spectrometry (LC-MS) was performed on an Agilent 1200 Series high-performance liquid chromatography (HPLC) system equipped with Agilent InfinityLab Poroshell 120 EC-C18 (2.1 mm × 50 mm, 2.7 μm) and EC-C8 (2.1 mm × 50 mm, 2.7 μm) columns maintained at 40 °C, a diode array detector (210 and 254 nm), and an Agilent 6120 Single Quadrupole mass spectrometer (ESI). The LC-MS mobile phase consisted of either HPLC-grade H_2_O/MeOH (0.1% formic acid (FA)) or H_2_O/MeCN (0.1% FA) during reaction monitoring and final compound purity analysis. LC-MS samples were prepared with HPLC-grade MeOH at a concentration of 1 mg/mL and analyzed with a 2 μL injection. Final compound purity was assessed as % of AUC_total_ at 254 nm using LC-MS and determined to be ≥95% pure for all samples submitted *in vitro* and *in vivo* (e.g., see Figure S12).

Chiral purification of **(*****rac*****)-2i** was performed on a Teledyne Isco ACCQPrep HP150 equipped with a Daicel Chiralcel OD-RH (21 mm × 250 mm, 5 μm) and a 5 mL sample loop. **(*****rac*****)-2i** was first dissolved in methanol (10 mg/mL) and then diluted with an eluant mixture (60:40 MeCN:H_2_O, 0.1% FA) to a final concentration of 5 mg/mL. The resulting solution was filtered with a 0.45 μm nylon filter and subsequently injected at 3.25 mL per run (10 mL/min for 21.3 min). Enantiomeric excess (e.e.) was determined using an Agilent 1100 HPLC instrument equipped with a ChiralPAK OD-RH column (4.6 mm × 150 mm, 5 μm). An e.e. of 99% was determined both for (S)-2i and (R)-2i. Specific rotation for the two enantiomers was (peak 1) (*S*)-**2i**: c = 1 in CHCl_3_, ∝_589_^20^ = −6.200 and (peak 2) (*R*)-**2i**: c = 1 in CHCl3 ∝_589_^20^ = +6.200.

Specific methods of synthesis for all compounds are listed in Supporting Methods.

### Voltage-Clamp Analysis of NMDA Receptors

#### Two-Electrode Voltage-Clamp Recordings

Unfertilized *Xenopus laevis* oocytes were obtained from Ecocyte (Austin, TX), or ovaries were purchased from *Xenopus* 1 (Dexter, MI) and oocytes prepared as previously described.^42,47^ Two-electrode voltage-clamp recordings were performed on oocytes expressing recombinant rat GluN1/GluN2A, GluN1/GluN2B, GluN1/GluN2C, or GluN1/GluN2D. cDNAs for rat GluN1-1a (referred to as GluN1; NCBI Reference Sequence NM_017010.2), GluN2A (NM_012573.4), GluN2B (NM_012574.1), GluN2C (NM_012575.3), and GluN2D (NM_022797.2) were provided by Dr. S. Heinemann from the Salk Institute, Dr. S. Nakanishi from Kyoto University, and Dr. P. Seeburg from the University of Heidelberg. Oocyte isolation, cRNA synthesis, and cRNA injections were performed as previously described.^32,33,47^ Briefly, *Xenopus laevis* oocytes were injected with 5–10 ng of cRNA in RNase-free water with the GluN1:GluN2 ratio ranging from 1:1 to 1:5. Oocytes were incubated at 15–19 °C in Barth’s solution consisting of (in mM) 88 NaCl, 1 KCl, 2.4 NaHCO_3_, 10 HEPES, 0.82 MgSO_4_, 0.33 Ca(NO_3_)_2_, and 0.41 CaCl_2_ supplemented with 100 μg/mL gentamycin, 40 μg/mL streptomycin, and 50 μg/mL penicillin. Recordings were performed 2–4 days after injection with extracellular recording solution containing (in mM) 90 NaCl, 1 KCl, 10 HEPES, 0.5 BaCl_2_, and 0.01 EDTA at pH 7.4 adjusted with NaOH. To prevent a gradual increase in current response over the course of the experiment, some oocytes expressing GluN1/GluN2A were injected with 20–50 nL of 2–50 mM K-BAPTA. Concentration–response curves for test compounds were generated by applying a maximally effective concentration of glutamate (100 μM) and glycine (30 μM), followed by glutamate and glycine with variable concentrations of test compounds up to 30 μM. The test compounds were prepared as 20 mM stock solutions in DMSO and diluted to the final concentration in recording solution. The DMSO content was 0.05–0.5% (v/v). Some low potency compounds (**2l, 2o, 2p**, and **2r**) with μM IC_50_ values were tested in 2, 5, or 10 mM hydroxypropyl-betacyclodextrin. Methods for off-target analysis are given in the Supporting Information.

#### Whole-Cell Voltage-Clamp Recordings

HEK293 cells (HEK, ATCC CRL-1573) were plated on glass coverslips pretreated with 0.1 mg/mL poly-d-lysine and cultured in Dulbecco’s modified Eagle medium (DMEM) plus Glutamax (GIBCO 10569-10) supplemented with 10% fetal bovine serum, 10 U/mL of penicillin, and 10 μg/mL of streptomycin at 37 °C and 5% CO_2_. The cells were transiently transfected with cDNA encoding rat GluN1, GluN2D, and eGFP at a ratio of 1:1:5 by using the calcium phosphate precipitation method.^48^ After 24–48 h following the transfection, the cells were perfused with external recording solution that contained (in mM) 3 KCl, 150 NaCl, 0.01 EDTA, 1.0 CaCl_2_, 10 HEPES, and 22 d-mannitol (the pH was adjusted to 7.4 with NaOH). The patch electrodes (resistance 3–5 MΩ) were prepared from thin-walled glass micropipettes (TW150F-4, World Precision Instruments, Sarasota, FL) by a dual-stage glass micropipette puller (PC-10, Narishige, Tokyo, Japan) and filled with internal solution containing (in mM) 110 d-gluconate, 110 CsOH, 30 CsCl, 5 HEPES, 4 NaCl, 0.5 CaCl_2_, 2 MgCl_2_, 5 BAPTA, 2 NaATP, and 0.3 NaGTP. The pH was adjusted to 7.4 with CsOH; osmolality was adjusted to about 300–305 mOsmol/kg. The whole-cell current responses were evoked by application of maximally effectively concentrations of agonists (100 μM glutamate and 30 μM glycine) at a holding potential of −60 mV and recorded using an Axopatch 200B patch-clamp amplifier (Molecular Devices, Union City, CA). A two-barreled theta-glass micropipette was used for rapid solution exchange controlled by a piezoelectric translator (Burleigh Instruments, Newton, NJ). For some experiments, 500 μM NMDA and 250 μM glycine were briefly applied by pressure application from a patch pipette (tip 3–5 MΩ). The current responses were low pass filtered at 2 kHz 8-pole Bessel filter (−3 dB; Frequency Devices) and digitized at 20 kHz using Digidata 1440A acquisition system (Molecular Devices) controlled by Clampex 10.3 (Molecular Devices). All patch experiments were performed at room temperature (23 °C).

#### Data Analysis

Compound potency was determined by fitting the concentration–response curve with
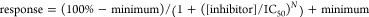
1where IC_50_ is the concentration of the inhibitor that produces a half-maximal effect, *N* is the Hill slope, and *minimum* is the degree of residual inhibition at a saturating concentration of the antagonist constrained to be greater than 0. Confidence intervals were determined for the log(IC_50_). The time course of relaxation during inhibitor application or deactivation following rapid removal of NMDA or glutamate was fitted by the equation
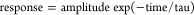
2

#### Homology Model

The homology model of GluN1/GluN2D shown in Figure 1 has been previously described.^49^

### Metabolic Stability Assays

#### Liver Microsome Stability

Human liver microsomes (HLMs, 20 mg/mL) and CD-1 mouse liver microsomes (MLMs, 20 mg/mL) were purchased from Xenotech. NADPH was purchased from Sigma-Aldrich and prepared in 10 mM stock solutions of distilled H_2_O (Invitrogen UltraPure). Verapamil and diphenhydramine were both purchased from Sigma-Aldrich and served as positive controls for HLM and MLM stability, respectively. Test compounds and positive controls were initially dissolved in DMSO to make 10 mM stock solutions. Sample solutions were then further diluted in 70% MeOH/H_2_O or 100% MeCN to 500 μM. Next, the reactions were prepared by mixing human or mouse liver microsomes (55 μL) with potassium phosphate buffer (100 mM, 928 μL) in 1.5 mL Eppendorf tubes. The test compounds (6.6 μL of 500 μM solution) were subsequently added to the suspensions, and the reaction mixtures were incubated at 37 °C for 5 min. Afterward, the liver microsome reactions were initiated with 110 μL of 10 mM NADPH and further incubated at 37 °C for the designated time course of the study. This procedure provided experiments with a final volume of 1100 μL (<0.6% organic solvent content), a concentration for HLMs and MLMs of 1 mg/mL, and a final test compound concentration of 3 μM. Aliquots (100 μL) were removed from each reaction mixture in duplicate at 0, 5, 10, 15, and 30 min time intervals and quenched with 100 μL of cold internal standard solution (ISTD, 2 μM 7-ethoxy-*d*_5_-coumarin in MeOH or MeCN). Quenched aliquots were then centrifuged at 12 500 g for 5–10 min, and the resulting supernatants were withdrawn and placed in LC-MS vials to be analyzed by LC-MS/MS (Agilent G6460C QQQ MS coupled with an Infinity II 1260 HPLC). Each test compound was run in tandem with positive and negative control experiments for quality assurance. Positive control reactions were conducted at a final volume of 550 μL for a single run at each time point. Lastly, the negative control experiment was conducted with test compounds and liver microsomes in the absence of NADPH (150 μL) and analyzed at the 30 min time point.

#### Plasma Stability

Human plasma (lithium heparin (LiHep) mixed, gender pooled, 0.2 μm filtered) and mouse plasma (BALB/C, LiHep mixed, male pooled, 0.2 μm filtered) were purchased from BioIVT. Procaine (Sigma-Aldrich) served as a positive control for both human and mouse plasma experiments. Test compounds and positive controls were initially dissolved in DMSO to make 10 mM stock solutions. The solutions of test and control compounds were then further diluted in 70% MeOH/H_2_O or 100% MeCN to 500 μM. Next, the human or mouse plasma (994 μL) was aliquoted into 1.5 mL Eppendorf tubes with duplicates (reactions A and B) being prepared for each compound. The plasma was then incubated at 37 °C for 10 min. Afterward, the reaction was initiated by the addition of the test compound (6 μL of 500 μM solution) and further incubated at 37 °C for the designated time course of the study. This procedure provided duplicate experiments with a final volume of 1000 μL (<0.6% organic solvent content) and a final test compound concentration of 3 μM. Aliquots (100 μL) were removed from each reaction mixture at 0, 15, 30, 60, and 120 min time intervals and quenched with 150 μL of cold ISTD solution (2 μM 7-ethoxy-*d*_5_-coumarin in MeOH or MeCN). Quenched aliquots were then centrifuged at 15,000 g for 30–45 min, and the resulting supernatants (∼70 μL) were withdrawn and placed in LC-MS vials to be analyzed by LC-MS/MS (Agilent G6460C QQQ MS coupled with an Infinity II 1260 HPLC). Each test compound was run in tandem with positive and negative control experiments for quality assurance. The positive control reaction was conducted at a final volume of 1000 μL for a single run at each time point. Finally, the negative control experiment was conducted with test compounds in DPBS (143 μL) and analyzed at the 120 min time point.

#### Data Analysis

For both LM and plasma assays, each data point was analyzed in triplicate using between blank washes to avoid carry over and to equilibrate the column for the subsequent runs. Averages of these triplicates for individual compounds at each time point were then normalized to the data at 0 min, representing 100% of the test compound remaining or 0% metabolism. Half-lives (*t*_*1/2*_) were calculated by plotting ln of % test compound remaining versus time and performing linear regression to determine slope. Slope = −*k* and *t*_1/2_ = 0.693/*k* for first-order kinetics (see Figures S5 and S6 for a representative example).

#### Brain Homogenate Tissue Binding and Stability

Mouse brain homogenate (C57BL/6) samples were prepared by diluting one volume of whole brain tissue with three volumes of PBS dialysis buffer (0.1 M sodium phosphate and 0.15 M sodium chloride, pH 7.4) to yield 4 times diluted homogenate. Carbamazepine (Apin Chemicals) served as a positive control for brain tissue binding. Test compounds and the positive control were initially dissolved in DMSO to make 1 mM stock solutions. The solutions of test and control compounds were then further diluted in a brain homogenate mixture to 5 μM (0.5% DMSO content). A rapid equilibrium dialysis (RED) device (Thermo Scientific) containing a dialysis membrane with a molecular weight cutoff of 8000 Da was used to assess tissue binding and metabolic stability. Each dialysis insert contained two chambers, including (1) a red chamber for brain homogenate and (2) a white chamber for buffer. The test compounds and positive control (200 μL of 5 μM solution) were added to the red chamber of the dialysis insert, while the white chamber was filled with dialysis buffer (350 μL). The RED device was then sealed with an adhesive film and incubated at 37 °C with shaking (300 rpm) for 4 h. For the initial time point (*t* = 0 min), 50 μL aliquots of test compounds and the positive control were added to 96-deep well plates (0.7 mL per well) and quenched with 400 μL of MeCN. Following dialysis, another 50 μL aliquot of the tested samples was removed from each well (brain homogenate and buffer) and diluted with an equal volume of the opposite matrix to nullify any matrix effects. All brain samples were then centrifuged (4000 rpm) at 4 °C for 10 min. The supernatant (100 μL) was subsequently transferred to 96-deep well plates for LC-MS/MS analysis. Prodrug metabolism and **2i** concentrations were determined by fit-for-purpose LC-MS/MS methods on a nine-point calibration curve ranging from 0.12 to 7.5 μM.

#### Fluorometric CYP450 Enzyme Inhibition Assays

Following a previously reported procedure,^50^ CYP450 inhibition assays utilized microsomes from insect cells that express recombinant human CYP2D6 and CYP3A4 isoforms (Corning) with substrates of CYP2D6 (3-[2-(*N*,*N*-diethyl-*N*-methylammonium)-ethyl]-7-methoxy-4-methylcoumarin iodide, AMMC) and CYP3A4 (7-benzyloxy-4-trifluoromethylcoumarin, BFC) capable of producing fluorescent metabolites.^51,52^ AMMC^58^ and BFC^59^ were synthesized in-house according to the literature procedures. All assay conditions including CYP enzyme concentration and incubation time were standardized, and IC_50_ values of test compounds were determined and validated on 2 separate days to confirm reproducibility. Test compounds were prepared in 100% DMSO, the final concentration of which did not exceed 0.2% in the enzymatic reaction mixture. A 100 mM sodium phosphate buffer was prepared and adjusted to a pH 7.4. In a separate falcon tube, a 2× enzyme–substrate (E–S) solution was prepared in phosphate buffer. The final concentrations of CYP2D6 and AMMC were 10 nM and 4 μM, respectively. The final concentrations of CYP3A4 and BFC were 20 nM and 40 μM, respectively. In a separate falcon tube, 2X solutions of the NADPH regenerating system (NRS) were prepared in phosphate buffer. The final concentrations for each NRS component in the corresponding assays were as follows: (1) CYP2D6 assay = 0.008 mM NADPH, 3.3 mM glucose-6-phosphate, and 0.4 U of glucose-6-phosphate dehydrogenase/mL and (2) CYP3A4 assay = 2.45 mM NADPH, 24.7 mM glucose-6-phosphate, and 1.25 U of glucose-6-phosphate dehydrogenase/mL.

Both enzymatic assays were conducted in a 96-well microtiter plate (Black, Corning Costar) with a final volume of 100 μL per well. Preparation of each plate began with the addition of 74 μL of the 2× E–S solution in the first well and 50 μL to all remaining wells (2–11) in the same row. The test compounds (1 μL of 10 mM DMSO stock solution) were then dissolved in the first well to give a final volume of 75 μL. 3-Fold serial dilutions of the test compounds were subsequently conducted by removing 25 μL from the first well and adding it to the second, followed by removing 25 μL from the second well and adding it to the third, and so forth until the 10th row. This procedure provided final concentrations for the test compound ranging from 100 to 0.01 μM across the same row (50 μL total for each well). Well 11 contained no test compound (0% inhibition and 100% activity). Well 12 contained 50 μL of substrate and test compound (100 μM) absent from the enzyme (0% activity, 100% inhibition). Wells 11 and 12 were used as controls for background fluorescence as described below. Each plate was then incubated for 30 min at 37 °C. After incubation was complete, reactions were initiated by the addition of 50 μL of 2X NRS to wells 1–12 (final well volume of 100 μL). Fluorescence measurements were immediately (<1 min) taken using a microplate reader (BioTek Synergy Neo 2). CYP2D6 was monitored at Ex/Em = 410/460 nm, and CYP3A4 was monitored at Ex/Em = 410/538 nm. Next, measurements were conducted in kinetic mode with scans every 5 min for 60 min. Two time points (30 and 60 min) were then chosen in the linear phase of the kinetic graph (reaction progression curve), and the corresponding relative fluorescence units (RFUs) at these time points were used to determine the rate of change in fluorescence according to the following equations
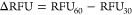
3
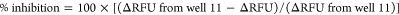
4
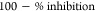
5where eq 3 defines the difference in RFUs between the 60 and 30 min time points, eq 4 normalizes the background-adjusted ΔRFU to no inhibitor control, and eq 5 converts % inhibition to % activity.

The resulting data were then exported and analyzed using Graph Pad Prism v7. The concentration of each test compound required to inhibit 50% substrate metabolism (IC_50_) was subsequently calculated from the resulting 10-point concentration–response curves (normalized fluorescence versus log[inhibitor]) using four-parameter logistic nonlinear regression (see Figure S4B for a representative example). Lastly, standard inhibitors ketoconazole (Sigma-Aldrich) and quinidine (Sigma-Aldrich) for CYP3A4 and CYP2D6, respectively, were evaluated for assay validation, and the calculated IC_50_ values for both ketoconazole (IC_50_ 67.9 nM) and quinidine (IC_50_ 17.9 nM) were compared to previously reported results.^53^

### Mouse Plasma and Brain Pharmacokinetics Experiments

IACUC-approved protocols and animal welfare regulations outlined in the “Guide for the Care and Use of Laboratory Animals” were both followed during the pharmacokinetic evaluation of all compounds. *In vivo* analysis was performed by Pharmaron (IP administration, Irvine, CA) and Sai Life (IV administration, Hinjewadi Pune, Pune, India).

#### IP Administration

A group (*n* = 15) of fed, male C57BL/6 mice ∼6 to 8 weeks of age was injected IP with 10 mg/kg (1 mg/mL) of drug using 50% PEG400 in water as a vehicle. Samples were collected from the blood and brain at 0.25, 0.5, 1, 2, and 4 h after administration (3 mice per time point) following CO_2_ anesthesia. Collection from the brain was performed as follows: the mouse is terminally anaesthetized via rising concentration of CO_2_ and as much blood is removed as possible via cardiac puncture. The cardiac puncture is done by opening the chest cavity to expose the heart, cutting an incision in the right auricle using surgical scissors, and finally injecting a saline solution (∼10 mL) slowly into the left ventricle via a syringe. The mouse is placed head down at a 45° angle to facilitate blood removal. After perfusion, the skull is opened and the brain is removed. The whole brain is washed with saline, dried with surgical gauze, placed in tared tubes, and stored at −75 °C before analysis. For plasma sample preparation at the 0.25–2 h time points, 15 μL of blank solution, 30 μL of plasma sample, and 150 μL were added sequentially for protein precipitation. After centrifugation, 20 μL of the supernatant and 80 μL of collection buffer were combined, and thus, the final compound concentration in plasma (ng/mL) was corrected by multiplying by 5. For plasma sample preparation at the 4 h time point, 15 μL of blank solution, 30 μL of plasma sample, and 150 μL of acetonitrile were added sequentially for protein precipitation. For all brain sample preparation, 15 μL of blank solution, 30 μL of brain samples, and 150 μL of acetonitrile were added sequentially for protein precipitation. Brain samples were prepared by adding brain (g) to deionized water (mL) in a 1:4 ratio for homogenization. The mixtures were then vortexed for 30 s and subsequently centrifuged (∼4000 rpm) for 15 min. The supernatant was diluted 3-fold with water, and a 2 μL aliquot of the diluted supernatant was injected into a Shimadzu LC-30A LC-MS/MS system with a Phenomenex 2.6 μ PFP 100A column (30 mm × 2.1 mm) using verapamil as an internal standard. A gradient from 95% water (0.1% formic acid) to 95% ACN (0.1% formic acid) was run over 2 min at a flow rate of 0.6 mL/min. Brain and blood samples were collected at 15, 30, 60, 120, and 240 min from three C57Bl/6 mice at each time point. See Tables S6–S10 for **2i** concentrations.

#### IV Administration

Nine male C57BL/6 mice were administered intravenously (IV) with solution formulation of prodrug at 5 mg/kg dose (5 mL/kg) in 5% *N*-methyl-2-pyrrolidine, 5% Solutol HS-15, and 90% saline. Blood samples (∼60 μL) were collected under light isoflurane anesthesia from a set of three mice at 0.25, 1, and 3 h. Plasma was harvested by centrifugation of blood and stored at −70 ± 10 °C until analysis. Immediately after the collection of blood, brain samples were collected from each mouse at respective time points. Brain samples were homogenized using ice-cold phosphate-buffered saline (pH 7.4), and homogenates were stored below −70 ± 10 °C until analysis. The total homogenate volume was 3 times that of the brain weight. The plasma and brain concentration–time data of compounds **2i** were used for the pharmacokinetic analysis. Plasma and brain samples were quantified by the fit-for-purpose LC-MS/MS method (LLOQ: 2.06 ng/mL for plasma and 5.16 ng/mL for brain). Tables S11 and S12 show the **2i** concentrations.

### Acute Epileptic Mouse Model

All experiments were conducted in agreement with the requirements of the European Directive 2010/63/EU. Mice were housed in ventilated, light-tight, sound-isolated chambers under a standard 12:12 light/dark cycle (light on at 07.00 PM and light off at 07.00 AM) with food and water available ad libitum. C57BL/6J wild-type (*Tsc1*^*+/+*^) females from Janvier Laboratories (France) were crossed with heterozygote *Tsc1*^*±*^ male mice with the genetic background B6;129S4-*Tsc1*^*tm1.1Djk*^/Nci. The genotyping of pups issuing from this cross-breeding was performed on tail tissue samples at postnatal day P9. The study was conducted in *Tsc1*^*±*^ male mice at P14–16. Pups from at least three deliveries for each condition were studied to minimize the potential sampling bias.

The DQP derivative **2i** was diluted in DMSO at a concentration of 100 mM before being added to 100 to 150 μL of saline solution and intraperitoneally injected in mice. Intraperitoneal injection was administered during the EEG recordings at three different concentrations: 7, 14, and 28 mg/kg to estimate the dose–response relationship.

Experiments were performed on postnatal days of P14 and P16 male *Tsc1*^*±*^ mice. Surgery was performed under isoflurane anesthesia and lidocaine analgesia. During recordings, the head was fixed to the frame of a stereotaxic apparatus by attached bars; animals were surrounded by a cotton nest and heated via a thermal pad (36.6–37.7 °C). A silver chloride reference electrode was placed in the cerebellum. EEG recordings were performed with nonanaesthetized head-restrained *Tsc1*^*±*^ mice. A 16 site linear silicon probe (100 μm separation distance between recording sites, Neuronexus Technologies, MI) was placed into the somatosensory cortex using the Paxinos and Franklin atlas (2001)^54^ at coordinates anterior–posterior = 2–2.5 mm, mediolateral = 2–3 mm from Bregma, 1.2–1.5 mm from the dural surface, in order to trace the columnar activity at all layers. Signals were amplified (×100) and filtered at 3 kHz using a 16-channel amplifier (A-M systems, Inc.), digitized at 10 kHz, and then saved to the hard disk of a PC using Axoscope software (Molecular Devices). Recordings were analyzed offline using Clampfit (Molecular Devices, San Jose) and Origin (MicroCal, Northampton, MA) software. After recording, the position of the silicone probe was verified visually by DiI staining of the electrode in 100 μm coronal sections from the fixed brain. We considered that multiunit activity occurred in epileptic discharges if they appeared in a group of multiple spikes whose amplitude exceeded the background activity within a period lasting for at least 20 s. During EEG recordings, animals were monitored visually to determine the behavioral correlates of each electrographic epileptic discharge. During the experiment, EEG recordings were performed for 2 h prior to drug administration, after which drug was intraperitoneally injected with dosages of 7, 14, or 28 mg/kg to investigate its acute effects on *in vivo* epileptic seizures. EEG recordings then were performed for 2–2.5 h post injection.

## Supporting Information Available

Glutamate dependence of DQP-1105 and (*S*)-**2a**; agonist dependence of **DQP-1105** (Figure S1); agonist dependence of **2i** (**DQP-997**-**74**) on GluN1/GluN2C receptors (Figure S2); agonist dependence of (S)-**2a** (Figure S3); analysis of off-target actions; methods for metabolic stability assays; methods for kinetic solubility experiments; methods for X-ray crystal structure determination; supplemental synthetic methods; solubility and actions on CYP2D6 and CYP3A4 (Figure S4); microsomal stability for compound **2i** (**DQP-997**-**74**) (Figure S5); plasma stability for compound **2i** (**DQP-997**-**74**) (Figure S6); chiral separation of **(*****rac*****)-2i** (**DQP-997**-**74**) (Figure S7); separation of enantiomers of compound **2i** (**DQP-997**-**74**) (Figure S8); crystal structure of (*S*)**-2i or** ((*S*)-**DQP-997**-**74**) (Figure S9); one of two independent “target” molecules in the asymmetric unit hydrogen bonded to 50% water and 50% methanol occupying the same site of the structure (Figure S10); stereoselective actions of **2i** (**DQP-997**-**74**) (Figure S11); analysis of compound **2i** (**DQP-997**-**74**) purity (Figure S12); ^1^H NMR for **2i** (**DQP-997**-**74**) (Figure S13); ^13^C NMR for **2i** (**DQP-997**-**74**) (Figure S14); *in vitro* NMDAR activity and selectivity profiles for DQP analogues (Table S1); summary of mouse brain tissue binding and stability for test compounds and positive control (Table S2); structure and predicted ADME parameters for DQP analogues and prodrugs (Table S3); scan parameters and corresponding transitions in MRM mode for tested compounds (Table S4); summary of **2i** (**DQP-997**-**74**) formation in mouse brain homogenate after 4 h incubation for ester and amide prodrugs (Table S5); off-target actions of (*S*)-(−)-**2i** or (*S*)-(−)- (**DQP-997**-**74**) (Table S6); summary of **2i** (**DQP-997**-**74**) plasma exposure after a single IP injection (10 mg/kg) (Table S7); summary of **2i** (**DQP-997**-**74**) brain exposure after a single IP injection (10 mg/kg) (Table S8); individual plasma concentration–time data of **2i** (**DQP-997**-**74**) following IP injection (10 mg/kg) (Table S9); individual brain concentration–time data of **2i** (**DQP-997**-**74**) following IP injection (10 mg/kg) (Table S10); individual brain/plasma ratio–time data of **2i** (**DQP-997**-**74**) following IP injection (10 mg/kg) (Table S11); individual plasma concentration–time data of **2i** (**DQP-997**-**74**) following IV injection (5 mg/kg) (Table S12); and individual brain concentration–time data of **2i** (**DQP-997**-**74**) following IV injection (5 mg/kg) (Table S13) (PDF)
