# Supplementary material for: Development of a Dihydroquinoline–Pyrazoline GluN2C/2D-Selective Negative Allosteric Modulator of the N-Methyl-d-aspartate Receptor
Source: ACS Chem Neurosci. 2023 Aug 11;14(17):3059–76. doi: 10.1021/acschemneuro.3c00181 (PMC10485906; doi:10.1021/acschemneuro.3c00181)
Supplement: Supplementary file 1 — cn3c00181_si_001.pdf [file cn3c00181_si_001.pdf]

Supporting Information

Development of a Dihydroquinoline-Pyrazoline GluN2C/2D-Selective Negative Allosteric Modulator of the N-Methyl-D-Aspartate Receptor

Michael P. D'Erasmo[a+], Nicholas S. Akins[a+], Peipei Ma[a], Yao Jing[a], Sharon A. Swanger[b], Savita K. Sharma[a], Perry W. Bartsch[a], David S. Menaldino[a], Paul J. Arcoria[a], Thi-Thien Bui[c], Alexandre Pons-Bennaceur[c], Phuong Le[b], James P. Allen[b], Elijah Z. Ullman[b], Kelsey A. Nocilla[b], Jing Zhang[b], Riley E Perszyk[b], Sukhan Kim[b], Timothy M. Acker[a], Azmain Taz[a], Samantha L. Burton[a], Kevin Coe[d], Russell G. Fritzemeier[a], Nail Burnashev[c], Hongjie Yuan[b], Dennis C. Liotta[a]*, and Stephen F. Traynelis[b]*,

[a] *Department of Chemistry, Emory University, Atlanta, GA, United States*
[b] *Department of Pharmacology and Chemical Biology, Emory University, Atlanta, GA, United States*
[c] *INMED, INSERM, Aix Marseille University, France.*
[d] *Janssen Research & Development, LLC, San Diego, CA 92121*

*Co-corresponding authors

[+]Co-first Authors

Table of Contents

Glutamate dependence of DQP-1105 and (*S*)-**2a**

Figure S1. Glutamate-dependence of **DQP-1105**

Figure S2. Agonist-dependence of **2i** (**DQP-997-74**) on GluN1/GluN2C receptors

Figure S3. Glutamate-dependence of (S)-**2a**

Analysis of Off-target Actions

Methods for Metabolic Stability Assays

Methods for Kinetic Solubility Experiments

Methods for X-ray Crystal Structure Determination

Supplemental Synthetic Methods

Figure S4: Solubility and actions on CYP2D6 and CYP3A4

Figure S5: Microsomal stability for compound **2i** (**DQP-997-74**)

Figure S6: Plasma stability for compound **2i** (**DQP-997-74**)

Figure S7. Chiral separation of **(*rac*)-2i** (**DQP-997-74**)

Figure S8. Separation of enantiomers of compound **2i** (**DQP-997-74**)

Figure S9. The crystal structure of (*S*)**-2i or** ((*S*)-**DQP-997-74**)

Figure S10. One of two independent 'target' molecules in the asymmetric unit hydrogen bonded to 50% water and 50% methanol occupying the same site of the structure

Figure S11. Stereoselective actions of **2i** (**DQP-997-74**)

Figure S12. Analysis of compound **2i** (**DQP-997-74**) purity

Figure S13. ^{1}H NMR for **2i** (**DQP-997-74**)

Figure S14. ^{13}C NMR for **2i** (**DQP-997-74**)

Table S1. *In Vitro* NMDAR Activity and Selectivity Profiles for DQP Analogs

Table S2. Summary of mouse brain tissue binding and stability for test compounds and positive control

Table S3. Structure and Predicted ADME Parameters for DQP Analogs and Prodrugs

Table S4. Scan parameters and corresponding transitions in MRM mode for tested compounds

Table S5. Summary of **2i** (**DQP-997-74**) formation in mouse brain homogenate after 4 hours incubation for ester and amide prodrugs

Table S6. Off-target actions of (*S*)-(-)-**2i** or (*S*)-(-)- (**DQP-997-74**)

Table S7. Summary of **2i** (**DQP-997-74**) plasma exposure after a single IP injection (10 mg/kg)

Table S8. Summary of **2i** (**DQP-997-74**) brain exposure after a single IP injection (10 mg/kg)

Table S9. Individual plasma concentration-time data of **2i** (**DQP-997-74**) following IP injection (10 mg/kg)

Table S10. Individual brain concentration-time data of **2i** (**DQP-997-74**) following IP injection (10 mg/kg)

Table S11. Individual brain/plasma ratio-time data of **2i** (**DQP-997-74**) following IP injection (10 mg/kg)

Table S12. Individual plasma concentration-time data of **2i** (**DQP-997-74**) following IV injection (5 mg/kg)

Table S13. Individual brain concentration-time data of **2i** (**DQP-997-74**) following IV injection (5 mg/kg)

Glutamate dependence of DQP-1105, (*S*)-2a, and 2i

Supplemental **Figure S1A** shows the structure of racemic **DQP-1105** (Acker et al., 2011) and **Figure S1B** shows whole cell current recordings from HEK cells of GluN1/GluN2D responses to glutamate with pre-applied **DQP-1105** or vehicle (0.1% DMSO). The mean IC_{50} for DQP-1105 was 1.9-fold higher during the peak response (IC_{50} 1.4 μM) compared to the steady-state current response (IC_{50} 0.72 μM; **Figure S1C; Table 2**) suggesting higher potency during steady-state current responses, consistent with increased affinity following glutamate binding. To investigate the time course of **DQP-1105** inhibition, the relaxation of the current response was fitted with a single exponential function. If the current response time course reflects **DQP-1105** binding, then the reciprocal of $\tau_{inhibition}$ should be linearly related to its concentration with the slope equal to k_{ON} and intercept equal to k_{OFF}. Figure **S1C** shows concentration-response curves for peak and steady-state responses normalized to control, expanded in **Figure S1D** to show the dependence of tau on the concentration of pre-applied drug. **Figure S1E** presents the deactivation period following glutamate removal; $\tau_{deactivation}$ is given for individual cells as well as the mean ± SEM. Data were compared by mixed effects model, F (1.879, 13.78) = 11.67, p = 0.001 with asterisks indicating statistically significant differences. $1/\tau_{inhibition}$ was correlated with **DQP-1105** concentration (R^2 = 0.82), and linear regression analysis yielded values of 4.8 x 10^5 M^{-1} s^{-1} for k_{ON} and 0.19 s^{-1} for k_{OFF} suggesting a K_D value of 0.40 μM. Interestingly, the time course of GluN1/GluN2D receptor deactivation following glutamate removal ($\tau_{deactivation}$ 5.5 ± 0.3 s) was prolonged in the presence of **DQP-1105** at concentrations of 0.3 μM (6.6 ± 0.3 s; p = 0.002), 1.0 μM (6.9 ± 0.4; p = 0.02), and 3 μM (8.0 ± 0.4 s; p = 0.003; **Figure S1E**). **Figure S1F** shows whole cell current recording of responses to five brief pressure-applied NMDA (500 μM) and glycine (250 μM) pulses at 1 s intervals with the responses in the absence or increasing concentrations of **DQP-1105** superimposed. **Figure S1G** shows the concentration-response curves of current responses for the first and last pulse normalized to control.

DQP-1105 IC_{50} was 3-fold higher for the first pulse (IC_{50} 4.9 μM) compared to the 5th pulse (IC_{50} 1.6 μM), which is consistent with increased inhibitor potency late in the stimulus train after agonist is bound.

Supplemental **Figure S2** shows an evaluation of the agonist-dependence of **2i** (**DQP-997-74**) inhibition of recombinant GluN1/GluN2C receptors expressed in HEK cells. GluN1/GluN2C current responses were recorded to glutamate with pre-applied glycine and compound **2i** or control (pre-applied glycine and vehicle, 0.1% DMSO; see **Figure S2A**). The concentration-response curves show peak and steady-state responses normalized to control (**Figure S2B**). A linear relationship exists between $1/\tau_{inhibition}$ and compound **2i** concentration from which k_{ON} and k_{OFF} can be calculated (see Results, **Figure S2C**). The deactivation time course following rapid glutamate removal was expanded to show the slowing of receptor deactivation ($\tau_{deactivation}$) by compound **2i** (**Figure S2D**). $\tau_{deactivation}$ for individual cells as well as the mean ± SEM are plotted and data were compared by mixed effects model ($F (4, 17) = 22.60$, $p<0.0001$; asterisks indicate statistically significant differences, see Results for p-values).

Supplemental **Figure S3A-B** shows the structure of **(*S*)-2a** (compound **DQP-69** in Acker et al., 2013) and the whole cell current recordings from HEK cells of GluN1/GluN2D responses to glutamate with pre-applied **(*S*)-2a** or vehicle (0.1% DMSO). The IC_{50} for **(*S*)-2a** [33] (**Figure S3**) was 4-fold higher during peak current responses (IC_{50} 2.1 μM) compared to steady-state responses (IC_{50} 0.51 μM; **Table 2**). **Figure S3C** shows the concentration-response curves for peak and steady-state responses normalized to control. Figure **S3D** presents the expanded current responses from panel **S1B** to show the concentration-dependent time course of relaxation following receptor activation during **(*S*)-2a** pre-application. A linear relationship exists between a $1/\tau_{inhibition}$ and **(*S*)-2a** concentration ($R^2 = 0.80$), and linear regression analysis yielded values of 5.4×10^5 M^{-1} s^{-1} for k_{ON} and 0.11 s^{-1} for k_{OFF}, suggesting a K_D of 0.20 μM. **Figure S3E** shows the slowing of receptor deactivation ($\tau_{deactivation}$) by **(*S*)-2a**. $\tau_{deactivation}$ for individual cells as well as the mean ± SEM. The data were compared by mixed effects model, $F (1.863, 9.35) = 30.90$, $p < 0.001$. Asterisks indicate statistically significant differences. The deactivation

of GluN1/GluN2D receptors following glutamate removal (5.2 ± 0.3 s) was slowed by the presence of **(*S*)-2a** at concentrations of 1 μM (9.2 ± 0.5 s; $p = 0.004$) and 3 μM (9.2 ± 0.5 s; $p = 0.011$), but not 0.03 μM (7.3 ± 0.6 s; $p = 0.061$) or 0.1 μM (5.6 ± 0.8 s; $p = 0.909$). **Figure S3F** shows whole cell current recording of responses to 5 brief pressure-applied NMDA (500 μM) and glycine (250 μM) pulses at 1 s intervals. The responses in the absence or increasing concentrations of **(*S*)-2a** are superimposed. Concentration-response curves in **Figure S3G** show current responses for the first and last pulse normalized to control. During trains of brief agonist applications, the IC_{50} of **(*S*)-2a** was 2.4-fold higher at the 1st pulse (IC_{50} 1.8 μM) compared to the 5th pulse (IC_{50} 0.49 μM; **Figure 5F,G; Table 2**). Altogether, these data suggest that **(S)-2a** exhibits a glutamate-dependent mechanism of action.

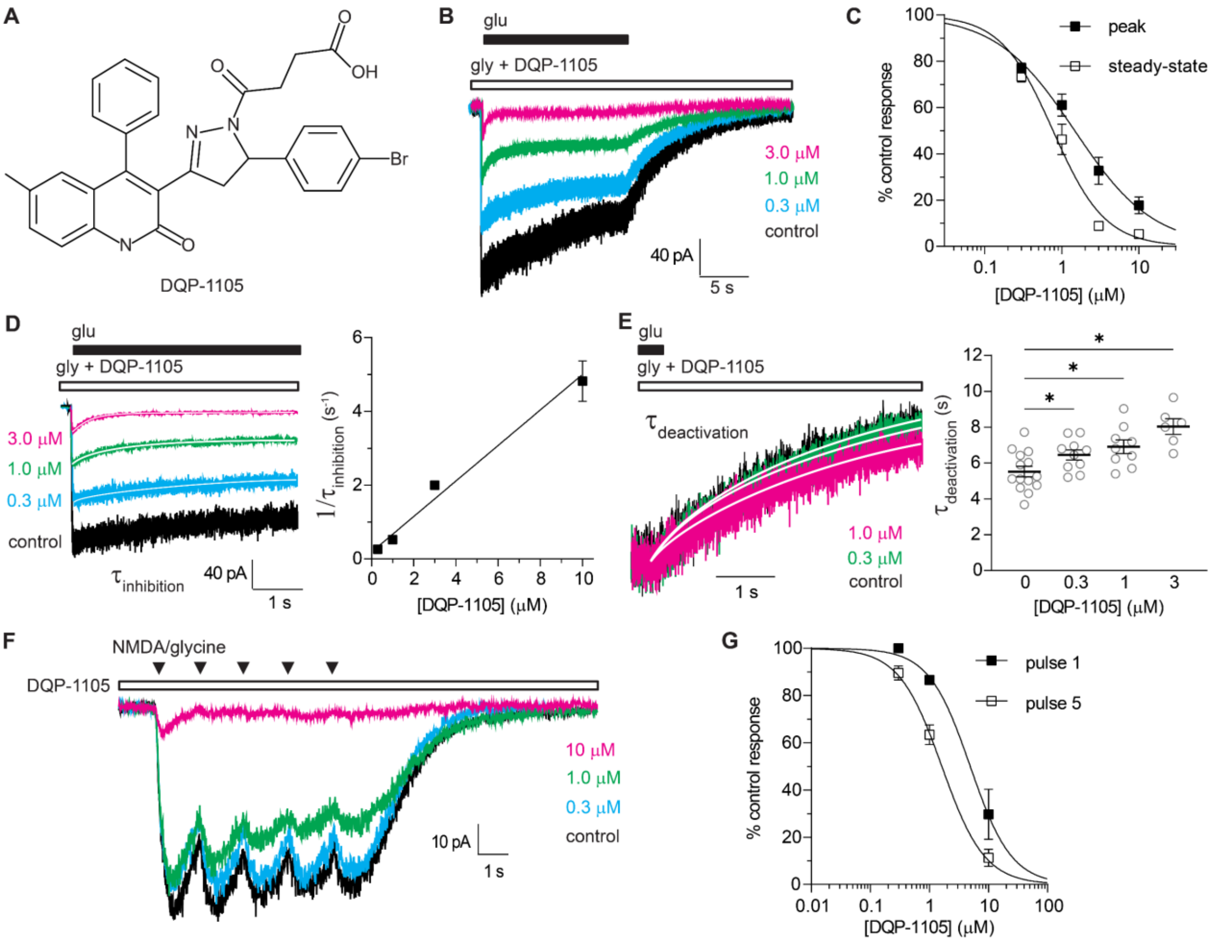

Supplemental Figure S1: Agonist-dependence of **DQP-1105** (see text above for description).

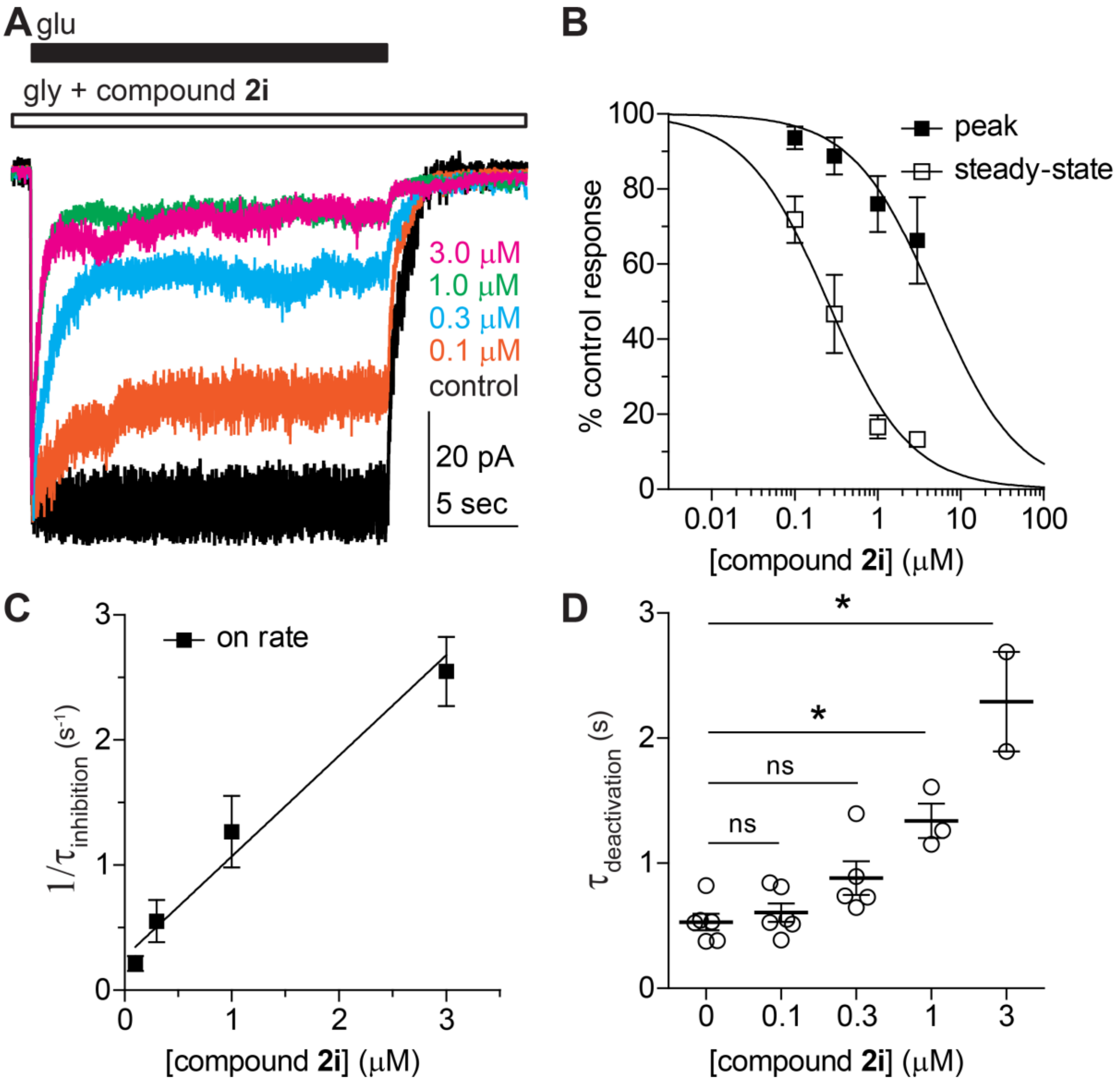

Supplemental Figure S2: Agonist-dependence of **2i** (**DQP-997-74**) on GluN1/GluN2C receptors (see text above for description).

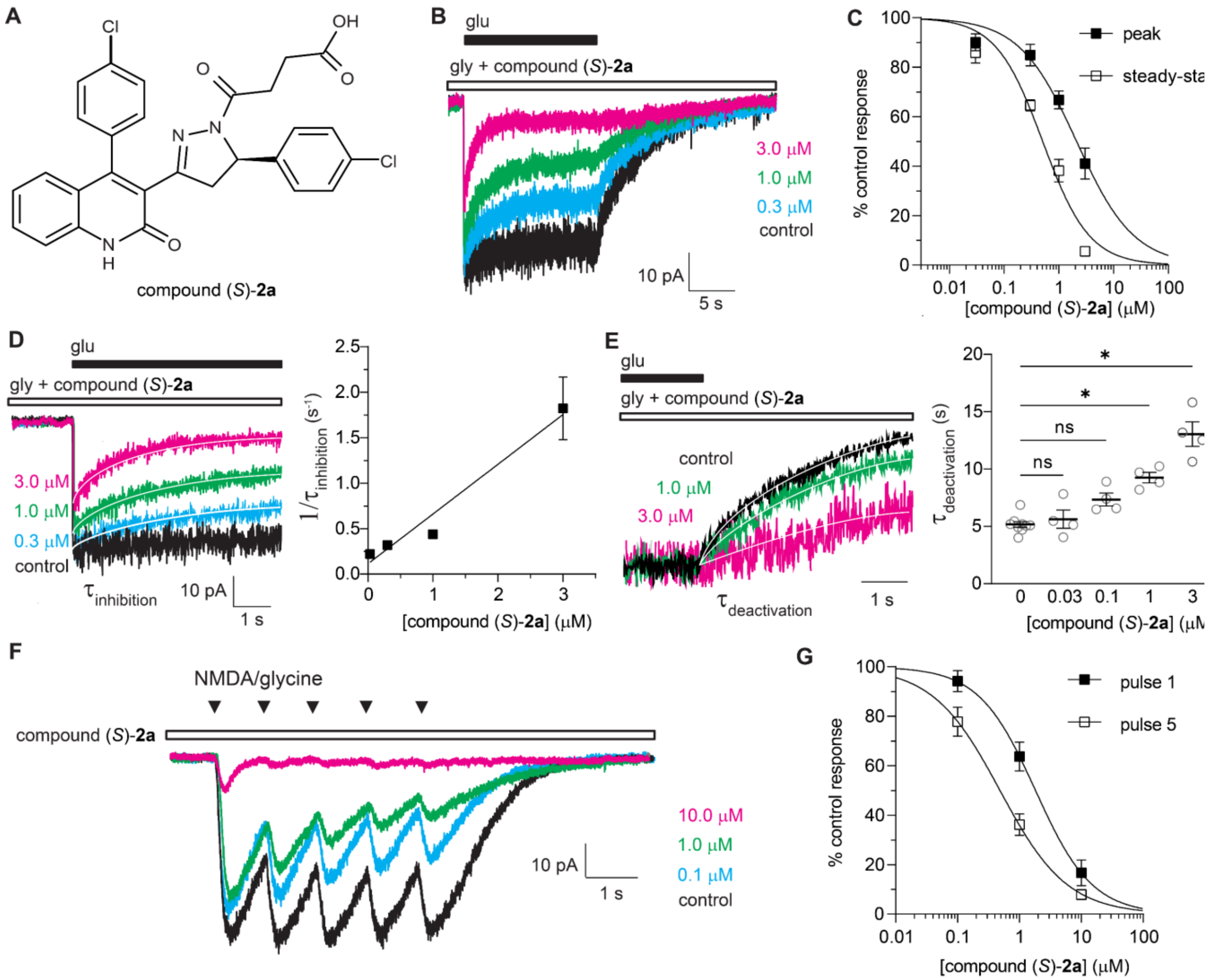

Supplemental Figure S3: Agonist-dependence of (*S*)-**2a** (see text above for description).

Analysis of Off-target Actions

Compound (*S*)-(-)-**2i** was tested at 1 μM for actions at glycine-activated GluN1/GluN3A (human GluN1-F484A,T518L/GluN3A referred to a GluN1-FA,TL/GluN3A) and rat GluN1-4a/GluN3B in the presence of 0.5 μM CGP-78608. cRNA injection ratios for GluN1/GluN3 were 1:2. Compound (*S*)-(-)-**2i** was also tested for actions at AMPA (rat GluA1, rat GluA2, human GluA3-L531Y), kainate (rat GluK2), nicotinic acetylcholine (rat α7 and α4β2, cRNA ratio 1:1), $GABA_A$ (rat $\alpha_1\beta_2\gamma_{2S}$, cRNA ratio 1:1:1), $GABA_C$ (rat ρ1), glycine (rat α1), and purinergic (human P_{2X}) receptors expressed in *Xenopus* oocytes. cRNA encoding the receptor subunits was synthesized from linear template cDNA using the mMessage mMachine kit (Ambion). Oocytes were injected with 1-5 ng cRNA in a 50 nL volume and incubated in Barth's solution at 15°C for 2-5 days prior to recording. GluK2-expressing oocyte were incubated in 1 mg/ml (10 μM) concanavalin-A for 10 min prior to recording. The cDNAs encoding $GABA_C$ and glycine subunits were provided by Dr. D. Weiss (University of Texas Health Science Center at San Antonio). cDNAs encoding nicotinic acetylcholine receptor subunits were provided by Drs. R. Papke (University of Florida) and S. Heinemann (Salk Institute). cDNAs encoding the purinergic receptors were provided by Dr. R. Hume (University of Michigan). $GABA_A$ and $GABA_C$ receptors were activated by 100 μM GABA. Acetylcholine was used at the indicated concentrations (in μM) to activate the nicotinic acetylcholine receptors: α4β2 (10), α7 (300). 100 μM glycine was used to activate the glycine receptor, 100 μM glycine was used to activate GluN1/GluN3 receptors, 100 μM glutamate was used to activate AMPA and kainate receptors, and 9 μM ATP was used to activate the purinergic receptors. Responses in presence of test compound were expressed as a percent of control.

Methods for Metabolic Stability Assays

LC-MS/MS analyses were conducted on an Agilent 1260 Infinity II HPLC equipped with an Agilent G6460 triple quadrupole mass spectrometer (Agilent Technologies, USA). Reverse-phase HPLC

separation for each compound was achieved with an Agilent InfinityLab Poroshell 120 EC-C18 (2.1 x 50 mm, 2.7 μm) or EC-C8 (2.1 x 50 mm, 2.7 μm) column maintained at 40 ^{o}C. The mobile phase used during analyses consisted of either MeOH-H_2O (0.1% FA) or MeCN-H_2O (0.1% FA) at a flow rate of 0.5 mL/min. Each method was developed in the presence of the internal standard (ISTD), d_5-7-ethoxy coumarin. Precursor and product ion detection was performed with Agilent Jet Stream electrospray positive ionization (ESI+) in multiple reaction monitoring (MRM) mode. All MRM transitions, fragmentor voltages, and collision energies for individual compounds are provided in **Supplemental Table S4**. Other standard MS conditions were as follows: dwell time of 100 ms; gas flow of 10 L/min; nebulizer pressure of 45 psi; delta EMV of 200 V. Lastly, all acquired data was processed using Agilent 6460 Quantitative Analysis software.

Methods for Kinetic Solubility Experiments

The test compounds were first dissolved in DMSO to provide stock solutions of either 30 mM or 60 mM. Kinetic solubility experiments were then performed in a 96-well microtiter plate (Clear-Bottom, Black, Corning® Costar) with a final volume of 250 µL per well in rows B-H. Each plate was initially prepared with the addition of 16 μL of DMSO to well A3, 20 μL to well A4, 27 μL to well A5, and 40 μL to all remaining wells in row A (A1 and A6-12). Next, the test compound (30 µL of DMSO stock solution) was directly added to well A2 without further modification. Moreover, the test compound (64 μL of DMSO stock solution) was diluted in well A3 to give a total volume of 80 μL. Further dilutions were then conducted by removing 60 µL from well A3 and adding it to A4, followed by removing 53 µL from well A4 and adding it to A5, followed by removing 40 µL from well A5 and adding it to A6, and sequentially removing 40 µL from the remaining wells up to A12. Well A1 served as a DMSO blank. The sample solutions prepared in row A (2.5 µL from each well) were transferred to the corresponding wells in rows B-H. Dulbecco's phosphate-buffered saline (DPBS, Gibco®, 1X, no Ca^{2+} and Mg^{2+}, pH

7.1-7.3) was then added to all wells in rows B-H (30 µL each), and the 96-well plate was incubated at 25 °C for 1 min. Next, DPBS (217.5 µL) was added to all wells in rows B-H, and the 96-well plate was once more incubated at 25 °C for 2 h. This procedure provided seven replicate experiments with final concentrations of the test compound (1% DMSO in DPBS) ranging from either 300 to 0.9375 µM or 600 to 1.875 µM in rows B-H, depending on the whether a 30 mM or 60 mM DMSO stock solution was used, respectively (i.e. a 100-fold dilution from sample preparation in row A). After incubation, the plates were analyzed using a NEPHELOstar® microplate reader, and the segmental linear regression data was processed with MARS analysis software (BMG LabTech, see **Supplemental Figure S4A** for a representative example).

Methods for X-ray Crystal Structure Determination

Single crystals of each peak isolated as described in the Methods were grown by slow evaporation of a solution of the compound in a mixture of methanol and water. Single colorless prism-shaped crystals of **(*S*)-2i** were chosen from the sample as supplied. A suitable crystal with dimensions 0.36 × 0.15 × 0.09 mm^3 was selected and mounted on a loop with paratone on a Rigaku Synergy-S diffractometer. The crystal was kept at a steady T = 100.0(3) K during data collection. The structure was solved with the ShelXT 2018/2 (Sheldrick, 2015, Bauer and Gotz, 2021) solution program using dual methods and by using Olex2 1.5-alpha (Dolomanov et al., 2009) as the graphical interface. The model was refined with olex2.refine 1.5-alpha (Bourhis et al., 2015) using full matrix least squares minimization on F2. Crystal data for $C_{57}H_{44}Cl_4F_4N_6O_{10}$, Mr = 1190.823, monoclinic, P21 (No. 4), a = 9.6576(1) Å, b = 15.6247(2) Å, c = 17.3239(2) Å, b = 90.827(1)°, a = g = 90°, V = 2613.85(5) Å3, T = 100.0(3) K, Z = 2, Z' = 1, m(Cu Ka) = 2.769, 27417 reflections measured, 8929 unique (Rint = 0.0541) which were used in all calculations. The final wR2 was 0.0983 (all data) and R1 was 0.0412 (I≥2 s(I)).

Supplemental Synthetic Methods

*4-(5-(4-Chlorophenyl)-3-(4-(4-chlorophenyl)-2-oxo-1,2-dihydroquinolin-3-yl)-4,5-dihydro-1H-pyrazol-1-yl)-4-oxobutanoic acid (**2a**).* Compound **6** (1.00 g, 2.30 mmol, 1.00 eq) and anhydrous THF (23.0 mL, 0.1 M) were added to a Schlenk flask equipped with a magnetic stir bar under an atmosphere of Ar. Succinic anhydride (276 mg, 2.76 mmol, 1.20 eq) dissolved in THF was then added dropwise, and the reaction mixture was heated to reflux for 5 h. After confirming complete conversion of starting material by LC-MS, the reaction mixture was cooled to room temperature and diluted with EtOAc. Next, the collected organics were washed with brine (2x), dried with $MgSO_4$, and concentrated under reduced pressure until a yellow solid was observed. The resulting crude solid was then suspended in DCM and spun down for 10 min at 5000 g. The organic layer was subsequently removed, and the precipitate was suspended and spun down several more times (4x). Finally, the washed solid was dried under high vacuum to yield a pale white powder (1.05 g, 1.96 mmol, 85% yield). ^{1}H and ^{13}C NMR data fully matched those previously reported in literature.[33] **LC-MS** (ESI, C8, 0.5 mL/min) 45-95% MeCN in H_2O, 10 min, RT = 2.067 min, *m/z* = 534.0 $[M + H]^+$; 35-95% MeCN in H_2O, 10 min, RT = 4.590 min, *m/z* = 534.0 $[M + H]^+$; 10-95% MeCN in H_2O, 10 min, RT = 7.153 min, *m/z* = 534.0 $[M + H]^+$.

*5-(5-(4-Chlorophenyl)-3-(4-(4-chlorophenyl)-2-oxo-1,2-dihydroquinolin-3-yl)-4,5-dihydro-1H-pyrazol-1-yl)-5-oxopentanoic acid (**2b**).* Compound **6** (300 mg, 0.691 mmol, 1.00 eq), 4 Å molecular sieves, and anhydrous THF (13.8 mL, 0.05 M) were added to a round-bottom flask equipped with a magnetic stir bar under an atmosphere of Ar. Glutaric anhydride (95 mg, 0.829 mmol, 1.20 eq) dissolved in THF was then added dropwise, and the reaction mixture was heated to reflux for 5 h. After confirming complete conversion of starting material by LC-MS, the reaction mixture was concentrated under reduced pressure and immediately purified by reversed-phase column chromatography eluting along a gradient of 0-80% MeOH in H_2O. Finally, fractions containing the desired product were collected, concentrated under reduced pressure, and dried under high vacuum to yield a bright yellow solid (241

mg, 0.439 mmol, 63% yield). **TLC** (10% MeOH in DCM) R_f = 0.23. **^{1}H NMR** (400 MHz, $CDCl_3$) δ 12.65 (brs, 1H), 7.58 – 7.48 (m, 2H), 7.46 – 7.42 (m, 1H), 7.39 – 7.29 (m, 2H), 7.24 – 7.19 (m, 2H), 7.18 – 7.11 (m, 3H), 6.86 – 6.80 (m, 2H), 5.35 (dd, *J* = 11.9, 4.3 Hz, 1H), 3.70 (dd, *J* = 18.3, 12.0 Hz, 1H), 2.84 (dd, *J* = 18.3, 4.3 Hz, 1H), 2.62 – 2.48 (m, 2H), 2.47 – 2.30 (m, 2H), 2.03 – 1.83 (m, 2H). **^{13}C NMR** (100 MHz, $CDCl_3$) δ 178.7, 170.7, 162.9, 152.11, 152.09, 140.2, 137.9, 135.0, 133.7, 133.5, 132.1, 131.2, 130.3, 129.0 (2C), 128.8, 128.7, 127.9, 127.3 (2C), 123.6, 122.9, 120.3, 116.5, 59.2, 45.5, 33.7, 33.1, 20.0 (*27 out of 29 carbon signals observed due to chemical shift equivalence*). **HRMS** (NSI) *m/z* calculated for $C_{29}H_{24}Cl_2N_3O_4^+$ [M + H]$^+$: 548.11384, found 548.11369. **LC-MS** (ESI, C8, 0.5 mL/min) 70-95% MeCN in H_2O, 10 min, RT = 2.062 min, *m/z* = 548.0 [M + H]$^+$; 60-95% MeCN in H_2O, 10 min, RT = 7.926 min, *m/z* = 548.0 [M + H]$^+$.

*(2S)-2-Amino-5-(5-(4-chlorophenyl)-3-(4-(4-chlorophenyl)-2-oxo-1,2-dihydroquinolin-3-yl)-4,5-dihydro-1H-pyrazol-1-yl)-5-oxopentanoic acid (**2d**).* Compound **6** (250 mg, 0.576 mmol, 1.00 eq), Boc-L-glutamic acid 1-*t*-butyl ester (192 mg, 0.633 mmol, 1.10 eq), and anhydrous DMF (5.8 mL, 0.1 M) were added to a round-bottom flask equipped with a magnetic stir bar under an atmosphere of Ar. HBTU (327 mg, 0.863 mmol, 1.50 eq) and triethylamine (241 µL, 1.73 mmol, 3.00 eq) were then added to the solution, and the reaction mixture was stirred at room temperature overnight. After confirming complete conversion of starting material by TLC and LC-MS, the reaction mixture was diluted with EtOAc and quenched with saturated aqueous NH_4Cl. The aqueous layer was then separated, and the collected organics were washed with saturated aqueous NH_4Cl (3x), dried with Na_2SO_4, and concentrated under reduced pressure to yield a golden brown oil that was carried on without further purification. Next, the resulting crude residue was taken up in 50% (v/v) TFA/DCM (3 mL, 0.1 M) and added to a Biotage® microwave reaction vial (2-5 mL) equipped with a magnetic stir bar under an atmosphere of Ar. The reaction mixture was then heated to 70 ^{o}C under microwave irradiation for 2 min. After confirming complete conversion of starting material by TLC and LC-MS, the reaction mixture was

concentrated under reduced pressure and immediately purified by reversed-phase column chromatography eluting along a gradient of 0-80% MeOH in H_2O. Finally, fractions containing the desired product were collected, concentrated under reduced pressure, and dried under high vacuum to yield a pale yellow solid (93 mg, 0.165 mmol, 29% yield over two steps). **TLC** (20% MeOH in DCM) R_f = 0.27. **^{1}H NMR** (600 MHz, CD_3OD) δ 7.66 – 7.57 (m, 4H), 7.52 – 7.42 (m, 6H), 7.27 – 7.17 (m, 8H), 7.18 – 7.13 (m, 2H), 6.81 – 6.73 (m, 4H), 5.39 (dd, *J* = 4.4, 2.6 Hz, 1H), 5.37 (dd, *J* = 4.1, 2.7 Hz, 1H), 3.79 – 3.69 (m, 2H), 3.61 – 3.54 (m, 2H), 2.87 – 2.75 (m, 4H), 2.72 (dd, *J* = 18.6, 4.3 Hz, 1H), 2.67 (dd, *J* = 18.6, 4.3 Hz, 1H), 2.16 – 1.96 (m, 4H). **^{13}C NMR** (151 MHz, CD_3OD) δ 172.2 (2C), 172.1 (2C), 162.6 (2C), 155.5, 155.4, 153.12, 153.11, 141.67, 141.65, 139.82, 139.81, 135.97, 135.95, 135.04, 135.01, 134.2 (2C), 133.2, 133.1, 133.0 (2C), 131.7, 131.6, 129.94, 129.90, 129.84, 129.80, 129.8 (4C), 128.8 (2C), 128.4 (2C), 128.3 (2C), 124.5, 124.4, 124.26, 124.25, 121.3 (2C), 116.9 (2C), 60.47, 60.45, 55.9, 55.6, 46.83, 46.80, 31.9, 31.4, 27.4, 27.2 (*45 out of 58 carbon signals observed due to chemical shift equivalence*). **HRMS** (NSI) *m/z* calculated for $C_{29}H_{25}Cl_2N_4O_4^+$ [M + H]$^+$: 563.12474, found 563.12545. **LC-MS** (ESI, C8, 0.5 mL/min) 40-95% MeCN in H_2O, 10 min, RT = 1.053 min, *m/z* = 563.0 [M + H]$^+$; 5-95% MeCN in H_2O, 10 min, RT = 6.447 min, *m/z* = 563.0 [M + H]$^+$.

*4-(5-(4-Chlorophenyl)-3-(4-(4-chlorophenyl)-2-oxo-1,2-dihydroquinolin-3-yl)-4,5-dihydro-1H-pyrazol-1-yl)-4-oxobutanenitrile (**2e**).* Compound **6** (500 mg, 1.15 mmol, 1.00 eq), 3-cyanopropionic acid (137 mg, 1.38 mmol, 1.20 eq), and anhydrous DMF (12.0 mL, 0.1 M) were added to a Biotage® microwave reaction vial (10-20 mL) equipped with a magnetic stir bar under an atmosphere of Ar. While stirring the reaction mixture vigorously at room temperature, triethylamine (642 μL, 4.60 mmol, 4.00 eq) and HBTU (873 mg, 2.30 mmol, 2.00 eq) in DMF were added dropwise. The reaction vessel was then sealed and subjected to microwave radiation at 85 °C for 45 min. After confirming complete conversion of starting material by LC-MS, the reaction mixture was diluted with EtOAc. The collected organics were subsequently washed with saturated aqueous NH_4Cl (3x), dried with Na_2SO_4, and

concentrated under reduced pressure. The resulting crude residue was then purified by normal-phase column chromatography eluting along a gradient of 0-100% EtOAc in hexanes followed by a gradient of 0-1% MeOH in DCM. Finally, fractions containing the desired product were collected, concentrated under reduced pressure, and dried under high vacuum to yield a bright yellow solid (350 mg, 0.680 mmol, 59% yield). **TLC** (10% MeOH in DCM) R_f = 0.43. **^{1}H NMR** (400 MHz, $CDCl_3$) δ 12.58 (s, 1H), 7.63 – 7.55 (m, 1H), 7.55 – 7.49 (m, 2H), 7.38 – 7.34 (m, 1H), 7.32 – 7.27 (m, 3H), 7.26 – 7.22 (m, 1H), 7.22 – 7.19 (m, 2H), 7.04 – 6.99 (m, 2H), 5.40 (dd, *J* = 11.8, 4.1 Hz, 1H), 3.72 (dd, *J* = 18.4, 11.8 Hz, 1H), 3.18 (dd, *J* = 18.4, 4.2 Hz, 1H), 2.85 – 2.67 (m, 2H), 2.64 – 2.48 (m, 2H). **^{13}C NMR** (100 MHz, $CDCl_3$) δ 167.1, 162.5, 153.7, 152.0, 139.7, 138.3, 135.2, 133.87, 133.85, 132.2, 130.7, 130.5, 129.2 (2C), 129.0, 128.8, 128.2, 127.6 (2C), 123.6, 122.8, 120.2, 119.3, 116.3, 59.5, 45.5, 30.1, 12.5 (*26 out of 28 carbon signals observed due to chemical shift equivalence*). **HRMS** (APCI) *m/z* calculated for $C_{28}H_{21}Cl_2N_4O_2^+$ [M + H]$^+$: 515.10361, found 515.10398. **LC-MS** (ESI, C8, 0.5 mL/min) 75-95% MeCN in H_2O, 10 min, RT = 1.558 min, *m/z* = 515.0 [M + H]$^+$; 65-95% MeCN in H_2O, 10 min, RT = 7.390 min, *m/z* = 515.0 [M + H]$^+$.

*3-(1-(3-(1H-Tetrazol-5-yl)propanoyl)-5-(4-chlorophenyl)-4,5-dihydro-1H-pyrazol-3-yl)-4-(4-chlorophenyl)quinolin-2(1H)-one (**2h**).* A solution of 1N NaOH (1.3 mL, 1.3 mmol) was added to methyl 3-(1*H*-tetrazol-5-yl)propanoate **7** (50 mg, 0.32 mmol) in MeOH (2.8 mL) and stirred at rt for 16 h. The mixture was concentrated, and the resulting solid was dissolved in water (1 mL) and acidified to pH = 3 with 1 N HCl. The aqueous phase was washed with DCM (2 × 1 mL) and the aqueous phase was concentrated to give 3-(1*H*-tetrazol-5-yl)propanoic acid **8** as a white solid that was used without further purification (35 mg crude mass). T3P (0.14 mL, 0.23 mmol) was added slowly to a mixture of **8** (35 mg, 0.25 mmol), 4-(4-chlorophenyl)-3-(5-(4-chlorophenyl)-4,5-dihydro-1*H*-pyrazol-3-yl)quinolin-2(1H)-one (50 mg, 0.12 mmol), and Et_3N (35 µL, 0.25 mmol) in DMF (1.5 mL) at 0 ºC, then stirred at rt for 16 h. The mixture was diluted with EtOAc (2 mL) and washed with water (2 × 1 mL). Flash chromatography

(0-10% MeOH/DCM), followed by RP HPLC (40-60% MeCN/H_2O) afforded the title compound as a white solid (27 mg, 64% yield). **^{1}H NMR** (400 MHz, CD_3OD) δ 7.64 – 7.53 (m, 2H), 7.48 – 7.41 (m, 3H), 7.29 – 7.08 (m, 5H), 6.86 – 6.70 (m, 2H), 5.37 (dd, *J* = 11.8, 4.4 Hz, 1H), 3.74 (dd, *J* = 18.3, 11.7 Hz, 1H), 3.21 – 2.90 (m, 4H), 2.78 (dd, *J* = 18.5, 4.4 Hz, 1H). **^{13}C NMR** (150 MHz, CD_3OD) δ 170.8, 167.6, 167.1, 162.6, 155.4, 153.1, 141.5, 139.9, 135.9, 135.2, 134.2, 133.0, 132.8, 131.7, 129.9, 129.8, 129.7, 129.6, 128.8, 128.3, 124.4, 124.2, 121.3, 116.9, 60.5, 46.8, 32.6, 19.8. **HRMS** (ESI) *m/z* calculated for $C_{28}H_{22}Cl_2N_7O_2^+$ $[M + H]^+$: 558.1207, found 558.1211.

*4-(5-(4-Chlorophenyl)-3-(4-(4-chlorophenyl)-2-oxo-1,2-dihydroquinolin-3-yl)-4,5-dihydro-1H-pyrazol-1-yl)-3,3-difluoro-4-oxobutanoic acid (**2i,** **DQP-997-74**).* 2,2-Difluorosuccinic acid (600 mg, 3.89 mmol, 1.30 eq) and anhydrous *i*-PrOAc (7.79 mL, 0.5 M) were added to a round-bottom flask equipped with a magnetic stir bar under an atmosphere of Ar. Trifluoroacetic anhydride (660 µL, 4.67 mmol, 1.56 eq) was then added to the solution dropwise at room temperature, and the reaction mixture was heated to 50 °C for 1.5 h. After confirming complete conversion of starting material by LC-MS tracing of 3,3-difluoro-4-methoxy-4-oxobutanoic acid ($[M + H]^+$ *m/z* = 169), the reaction mixture was concentrated using a rotary evaporator at 100 mbar of pressure and water bath temperature of 42 °C for 1.5 h and carried on without further purification. Next, the crude oil was taken up in anhydrous THF (10 mL) and cooled to 0 °C. Compound **6** (1.30 g, 3.00 mmol, 1.00 eq) diluted in THF was then added dropwise, and the reaction mixture was stirred at 0 °C for 1 h. After confirming complete consumption of compound **6** by LC-MS, the reaction mixture was diluted with EtOAc and carefully quenched with saturated aqueous NH_4Cl. After separating the aqueous layer, the collected organics were wash with brine, dried with $MgSO_4$, and concentrated under reduced pressure. Subsequently, the resulting crude oil was purified by normal-phase column chromatography eluting along a gradient of 0-5% MeOH in DCM. Fractions containing the desired product were then collected and concentrated under reduced pressure until a yellow solid was observed. The obtained solid was suspended in DCM and spun down for 10 min

at 5000 g. The organic layer was removed, and the precipitate was suspended and spun down several more times (4x). Finally, the washed solid was dried under high vacuum to yield a white powder (1.51 g, 2.65 mmol, 88% yield). **TLC** (20% MeOH in DCM) R_f = 0.34. **^{1}H NMR** (500 MHz, DMSO-d_6) δ 13.07 (brs, 1H), 12.33 (s, 1H), 7.61 – 7.56 (m, 1H), 7.56 – 7.52 (m, 1H), 7.52 – 7.47 (m, 1H), 7.45 – 7.40 (m, 2H), 7.32 – 7.28 (m, 2H), 7.27 – 7.24 (m, 1H), 7.18 – 7.12 (m, 1H), 7.04 – 6.99 (m, 1H), 6.88 – 6.83 (m, 2H), 5.50 (dd, *J* = 11.9, 4.5 Hz, 1H), 3.81 (dd, *J* = 18.7, 11.9 Hz, 1H), 3.47 – 3.13 (m, 2H), 2.73 (dd, *J* = 18.7, 4.6 Hz, 1H). **^{13}C NMR** (151 MHz, DMSO-d_6) δ 167.5 (dd, *J* = 9.4, 6.1 Hz), 159.9, 158.1 (t, *J* = 30.7 Hz), 156.1, 150.1, 139.8, 138.6, 133.7, 133.5, 131.9, 131.7, 131.1, 130.1, 128.7, 128.5, 128.3 (2C), 127.4 (2C), 127.3, 123.0, 122.5, 119.0, 115.6, 114.7 (t, *J* = 248.2 Hz), 59.5, 44.7, 40.9 (t, *J* = 25.4 Hz) (*26 out of 28 carbon signals observed due to chemical shift equivalence*). See **Supplemental Figures S12,S13** for spectra. **^{19}F NMR** (376 MHz, DMSO-d_6) δ -98.45 (dt, *J* = 265.3, 15.0 Hz), -99.37 (ddd, *J* = 264.9, 20.3, 13.2 Hz). **HRMS** (APCI) *m/z* calculated for $C_{28}H_{20}Cl_2F_2N_3O_4^+$ $[M + H]^+$: 570.07934, found 570.07850. **LC-MS** (ESI, C8, 0.5 mL/min) 65-95% MeCN in H_2O, 10 min, RT = 1.311 min, *m/z* = 570.0 $[M + H]^+$; 55-95% MeCN in H_2O, 10 min, RT = 4.591 min, *m/z* = 570.0 $[M + H]^+$; 40-95% MeCN in H_2O, 10 min, RT = 7.583 min, *m/z* = 570.0 $[M + H]^+$.

*4-(4-Chlorophenyl)-3-(5-(4-chlorophenyl)-1-(2,2,2-trifluoroacetyl)-4,5-dihydro-1H-pyrazol-3-yl)quinolin-2(1H)-one (**2j**).* Compound **6** (300 mg, 0.691 mmol, 1.00 eq), 4 Å molecular sieves, and anhydrous THF (13.8 mL, 0.05 M) were added to a round-bottom flask equipped with a magnetic stir bar under an atmosphere of Ar. After cooling the reaction mixture to 0 °C, trifluoroacetic anhydride (117 μL, 0.829 mmol, 1.20 eq) was added dropwise. The reaction mixture was then slowly warmed to room temperature and stirred for 1 h. Upon confirming complete conversion of starting material by LC-MS, the reaction mixture was diluted with EtOAc. The collected organics were subsequently washed with brine, dried with Na_2SO_4, and concentrated under reduced pressure. The resulting crude residue was then purified by normal-phase column chromatography eluting along a gradient of 0-60% EtOAc in hexanes.

Finally, fractions containing the desired product were collected, concentrated under reduced pressure, and dried under high vacuum to yield an off white solid (315 mg, 0.594 mmol, 86% yield). **TLC** (70% EtOAc in hexanes) R_f = 0.44. **^{1}H NMR** (400 MHz, $CDCl_3$) δ 12.77 (s, 1H), 7.63 – 7.55 (m, 1H), 7.52 – 7.45 (m, 2H), 7.39 – 7.36 (m, 1H), 7.35 – 7.32 (m, 1H), 7.32 – 7.28 (m, 2H), 7.25 – 7.22 (m, 1H), 7.22 – 7.19 (m, 2H), 7.02 – 6.96 (m, 2H), 5.45 (dd, *J* = 11.5, 3.8 Hz, 1H), 3.76 (dd, *J* = 18.4, 11.6 Hz, 1H), 3.12 (dd, *J* = 18.4, 3.9 Hz, 1H). **^{13}C NMR** (100 MHz, $CDCl_3$) δ 162.6, 156.8, 153.9 (q, *J* = 38.7 Hz), 152.5, 138.4, 138.1, 135.3, 134.4, 132.9, 132.3, 131.0, 130.6, 129.3 (2C), 128.81, 128.75, 128.2, 127.6 (2C), 123.6, 122.2, 120.2, 116.4, 116.0 (q, *J* = 287.1 Hz), 60.6, 45.0 (*24 out of 26 carbon signals observed due to chemical shift equivalence*). **^{19}F NMR** (376 MHz, $CDCl_3$) δ -71.52 (s). **HRMS** (NSI) *m/z* calculated for $C_{26}H_{17}Cl_2F_3N_3O_2^+$ [M + H]$^+$: 530.06444, found 530.06463. **LC-MS** (ESI, C8, 0.5 mL/min) 80-95% MeCN in H_2O, 10 min, RT = 1.744 min, *m/z* = 530.0 [M + H]$^+$; 65-95% MeCN in H_2O, 10 min, RT = 8.594 min, *m/z* = 530.0 [M + H]$^+$.

*General Procedure for **2i** Esterification and Amidation.* Compound **2i** (1.00 eq), DMF (1 μL), and anhydrous DCM (0.1-0.2 M) were added to a round-bottom flask equipped with a magnetic stir bar under an atmosphere of Ar. Oxalyl chloride (2.0 M in DCM, 2.00 eq) was then added to the suspension dropwise at room temperature. Next, the reaction mixture was heated to reflux for 5-10 min, then cooled to room temperature and stirred for 1.5-2 h or until complete conversion of starting material was confirmed by LC-MS. *Note: LC-MS sample was quenched with MeOH, and an [M+H]$^+$ adduct ion with m/z = 584 was observed.* The reaction mixture was subsequently concentrated under reduced pressure and dried under high vacuum for 2 h, producing the acyl chloride as a yellow foam which was taken on without further purification. The resulting acyl chloride intermediate was dissolved in anhydrous DCM (0.1-0.2 M), and the solution was cooled to 0 ^{o}C under an atmosphere of Ar. The corresponding alcohol or amine was then added dropwise, and the reaction mixture was stirred until complete conversion was confirmed by LC-MS. Next, the reaction mixture was diluted with EtOAc and carefully quenched with

brine, unless otherwise specified. The organic layer was then washed with brine (2x), dried with $MgSO_4$, and concentrated under reduced pressure. Lastly, the crude product was purified by column chromatography and/or washes with particular organic solvents to yield the final analog.

*4-(5-(4-Chlorophenyl)-3-(4-(4-chlorophenyl)-2-oxo-1,2-dihydroquinolin-3-yl)-4,5-dihydro-1H-pyrazol-1-yl)-3,3-difluoro-4-oxobutanamide (**2k**).* Synthesis was carried out according to general procedure for **2i** amidation using compound **2i** (250 mg, 0.438 mmol, 1.00 eq) and ammonia solution (0.4 M in THF, 3.30 mL, 1.32 mmol, 3.01 eq). After confirming complete conversion of the acyl chloride intermediate by LC-MS, the reaction mixture was quenched with ethanol. Purified via normal-phase column chromatography eluting along a gradient of 0-100% EtOAc in DCM followed by 0-5% MeOH in DCM. Fractions containing the desired product were subsequently collected and concentrated under reduced pressure until a yellow solid was observed. The obtained solid was then suspended in DCM and spun down for 10 min at 5000 g. The organic layer was removed, and the precipitate was suspended and spun down several more times (4x). Finally, the washed solid was dried under high vacuum to yield a white powder (115 mg, 0.202 mmol, 46% yield). **TLC** (10% MeOH in DCM) R_f = 0.27. **^{1}H NMR** (500 MHz, DMSO-d_6) δ 12.32 (s, 1H), 7.61 – 7.56 (m, 1H), 7.56 – 7.50 (m, 2H), 7.49 – 7.45 (m, 1H), 7.45 – 7.39 (m, 2H), 7.31 – 7.27 (m, 2H), 7.27 – 7.22 (m, 1H), 7.18 – 7.11 (m, 2H), 7.05 – 6.99 (m, 1H), 6.95 – 6.89 (m, 2H), 5.47 (dd, *J* = 11.9, 4.6 Hz, 1H), 3.79 (dd, *J* = 18.6, 12.0 Hz, 1H), 3.24 – 3.01 (m, 2H), 2.70 (dd, *J* = 18.7, 4.6 Hz, 1H). **^{13}C NMR** (151 MHz, DMSO-d_6) δ 166.7 (t, *J* = 6.7 Hz), 160.0, 158.5 (t, *J* = 30.5 Hz), 155.4, 150.1, 139.9, 138.6, 133.6, 133.4, 131.8, 131.6, 131.1, 130.1, 128.6, 128.5, 128.3 (2C), 127.5 (2C), 127.3, 123.1, 122.5, 119.0, 115.6, 115.4 (t, *J* = 247.3 Hz), 59.4, 44.7, 41.5 (t, *J* = 24.8 Hz) (*26 out of 28 carbon signals observed due to chemical shift equivalence*). **^{19}F NMR** (565 MHz, DMSO-d_6) δ -98.53 (dt, *J* = 259.9, 15.8 Hz), -99.11 (ddd, *J* = 260.6, 20.4, 13.8 Hz). **HRMS** (APCI) *m/z* calculated for $C_{28}H_{21}Cl_2F_2N_4O_3^+$ $[M + H]^+$: 569.09533, found 569.09545. **LC-MS** (ESI, C8, 0.5 mL/min) 50-95% MeCN in H_2O, 10 min, RT = 1.496 min, *m/z* = 569.0 $[M + H]^+$; 35-95% MeCN in H_2O, 10 min,

RT = 4.881 min, *m/z* = 569.1 $[M + H]^+$; 10-95% MeCN in H_2O, 10 min, RT = 7.325 min, *m/z* = 569.1 $[M + H]^+$.

*Ethyl 4-(5-(4-chlorophenyl)-3-(4-(4-chlorophenyl)-2-oxo-1,2-dihydroquinolin-3-yl)-4,5-dihydro-1H-pyrazol-1-yl)-3,3-difluoro-4-oxobutanoate (**2l**).* Synthesis was carried out according to general procedure for **2i** esterification using compound **2i** (300 mg, 0.526 mmol, 1.00 eq) and ethanol (200 proof, 2.00 mL, 15.7 mmol, 29.8 eq). Purified via normal-phase column chromatography eluting along a gradient of 0-60% EtOAc in DCM. Fractions containing the desired product were collected and concentrated under reduced pressure until yellow solid was observed. The obtained solid was then suspended in CH_3CN and spun down for 10 min at 5000 g. The organic layer was removed, and the precipitate was suspended and spun down several more times (4x). Finally, the washed solid was dried under high vacuum to yield a white powder (205 mg, 0.343 mmol, 65% yield). **TLC** (30% EtOAc in DCM) R_f = 0.28. **^{1}H NMR** (500 MHz, Chloroform-*d*) δ 12.67 (s, 1H), 7.62 – 7.55 (m, 1H), 7.50 – 7.44 (m, 2H), 7.39 – 7.34 (m, 1H), 7.33 – 7.27 (m, 3H), 7.23 – 7.16 (m, 3H), 7.05 – 6.99 (m, 2H), 5.49 (dd, *J* = 11.7, 3.9 Hz, 1H), 4.13 (dq, *J* = 10.8, 7.1 Hz, 1H), 4.06 (dq, *J* = 10.8, 7.1 Hz, 1H), 3.74 (dd, *J* = 18.3, 11.7 Hz, 1H), 3.36 – 3.12 (m, 2H), 3.05 (dd, *J* = 18.3, 4.0 Hz, 1H), 1.15 (t, *J* = 7.1 Hz, 3H). **^{13}C NMR** (151 MHz, $CDCl_3$) δ 166.2 (t, *J* = 7.2 Hz), 162.4, 159.3 (t, *J* = 30.4 Hz), 155.3, 152.1, 139.0, 138.4, 135.2, 134.0, 133.3, 132.2, 130.9, 130.4, 129.1 (2C), 129.03, 128.96, 128.1, 127.6 (2C), 123.6, 122.7, 120.2, 116.4, 114.6 (t, *J* = 252.7 Hz), 61.5, 60.6, 44.8, 41.1 (t, *J* = 25.7 Hz), 14.0 (*28 out of 30 carbon signals observed due to chemical shift equivalence*). **^{19}F NMR** (376 MHz, Chloroform-*d*) δ -99.38 (ddd, *J* = 273.3, 16.6, 12.5 Hz), -100.52 (ddd, *J* = 273.6, 18.2, 12.8 Hz). **HRMS** (APCI) *m/z* calculated for $C_{30}H_{24}Cl_2F_2N_3O_4^+$ $[M + H]^+$: 598.11064, found 598.11074. **LC-MS** (ESI, C8, 0.5 mL/min) 65-95% MeCN in H_2O, 6 min, RT = 1.148 min, *m/z* = 598.1 $[M + H]^+$; 45-95% MeCN in H_2O, 6 min, RT = 4.295 min, *m/z* = 598.1 $[M + H]^+$.

*Isopropyl 4-(5-(4-chlorophenyl)-3-(4-(4-chlorophenyl)-2-oxo-1,2-dihydroquinolin-3-yl)-4,5-dihydro-1H-pyrazol-1-yl)-3,3-difluoro-4-oxobutanoate (**2m**).* Synthesis was carried out according to general procedure for **2i** esterification using compound **2i** (500 mg, 0.877 mmol, 1.00 eq) and isopropanol (2.00 mL, 26.2 mmol, 29.8 eq). Purified via normal-phase column chromatography eluting along a gradient of 0-75% EtOAc in hexanes. Fractions containing desired product were collected, concentrated under reduced pressure, and then subjected a second time to normal-phase column chromatography eluting along a gradient of 0-1% MeOH in DCM. Finally, fractions containing the desired product were once more collected, concentrated under reduced pressure, and dried under high vacuum to yield a yellow foamy solid (315 mg, 0.515 mmol, 59% yield). **TLC** (30% EtOAc in DCM) R_f = 0.36. **^{1}H NMR** (500 MHz, $CDCl_3$) δ 12.48 (s, 1H), 7.62 – 7.56 (m, 1H), 7.51 – 7.43 (m, 2H), 7.38 – 7.31 (m, 2H), 7.31 – 7.27 (m, 2H), 7.24 – 7.17 (m, 3H), 7.04 – 6.98 (m, 2H), 5.48 (dd, *J* = 11.7, 4.0 Hz, 1H), 4.98 (hept, *J* = 6.4 Hz, 1H), 3.74 (dd, *J* = 18.3, 11.7 Hz, 1H), 3.31 – 3.11 (m, 2H), 3.02 (dd, *J* = 18.3, 4.0 Hz, 1H), 1.15 (d, *J* = 6.7 Hz, 3H), 1.14 (d, *J* = 6.5 Hz, 3H). **^{13}C NMR** (151 MHz, $CDCl_3$) δ 165.8 (t, *J* = 7.0 Hz), 162.4, 159.4 (t, *J* = 30.6 Hz), 155.2, 152.1, 139.0, 138.4, 135.2, 134.0, 133.3, 132.2, 131.0, 130.4, 129.2 (2C), 129.0, 128.9, 128.1, 127.6 (2C), 123.5, 122.7, 120.2, 116.3, 114.7 (t, *J* = 252.7 Hz), 69.3, 60.6, 44.8, 41.4 (t, *J* = 25.7 Hz), 21.7, 21.6 (*29 out of 31 carbon signals observed due to chemical shift equivalence*). **^{19}F NMR** (376 MHz, $CDCl_3$) δ -99.43 (ddd, *J* = 274.0, 17.0, 13.0 Hz), -100.54 (ddd, *J* = 274.0, 17.0, 13.2 Hz). **HRMS** (APCI) *m/z* calculated for $C_{31}H_{26}Cl_2F_2N_3O_4^+$ $[M + H]^+$: 612.12629, found 612.12566. **LC-MS** (ESI, C8, 0.5 mL/min) 65-95% MeCN in H_2O, 6 min, RT = 1.265 min, *m/z* = 612.1 $[M + H]^+$; 45-95% MeCN in H_2O, 6 min, RT = 4.561 min, *m/z* = 612.1 $[M + H]^+$.

*Cyclopropylmethyl 4-(5-(4-chlorophenyl)-3-(4-(4-chlorophenyl)-2-oxo-1,2-dihydroquinolin-3-yl)-4,5-dihydro-1H-pyrazol-1-yl)-3,3-difluoro-4-oxobutanoate (**2n**).* Synthesis was carried out according to general procedure for **2i** esterification using compound **2i** (150 mg, 0.263 mmol, 1.00 eq) and cyclopropylmethanol (213 μL, 2.63 mmol, 10.0 eq). Purified via normal-phase column chromatography

eluting along a gradient of 0-75% EtOAc in hexanes. Finally, fractions containing the desired product were then collected, concentrated under reduced pressure, and dried under high vacuum to yield a pale yellow solid (90.2 mg, 0.144 mmol, 55% yield). **TLC** (30% EtOAc in DCM) R_f = 0.39. **^{1}H NMR** (400 MHz, $CDCl_3$) δ 13.10 (s, 1H), 7.63 – 7.55 (m, 1H), 7.52 – 7.44 (m, 2H), 7.40 – 7.36 (m, 1H), 7.34 – 7.27 (m, 3H), 7.24 – 7.17 (m, 3H), 7.06 – 7.01 (m, 2H), 5.50 (dd, *J* = 11.7, 3.9 Hz, 1H), 3.92 (dd, *J* = 11.4, 7.3 Hz, 1H), 3.84 (dd, *J* = 11.4, 7.3 Hz, 1H), 3.74 (dd, *J* = 18.2, 11.7 Hz, 1H), 3.37 – 3.15 (m, 2H), 3.06 (dd, *J* = 18.4, 3.9 Hz, 1H), 1.08 – 0.96 (m, 1H), 0.57 – 0.46 (m, 2H), 0.28 – 0.14 (m, 2H). **^{13}C NMR** (100 MHz, $CDCl_3$) δ 166.4 (t, *J* = 7.3 Hz), 162.7, 159.3 (t, *J* = 30.5 Hz), 155.4, 152.1, 139.0, 138.5, 135.2, 133.9, 133.3, 132.2, 130.9, 130.4, 129.1 (2C), 129.0, 128.9, 128.0, 127.6 (2C), 123.5, 122.6, 120.2, 116.5, 114.6 (t, *J* = 252.9 Hz), 70.2, 60.6, 44.8, 41.1 (t, *J* = 25.9 Hz), 9.6, 3.44, 3.36 (*30 out of 32 carbon signals observed due to chemical shift equivalence*). **^{19}F NMR** (376 MHz, $CDCl_3$) δ -99.49 (ddd, *J* = 273.3, 16.6, 13.2 Hz), -100.56 (ddd, *J* = 273.3, 17.2, 13.3 Hz). **HRMS** (APCI) *m/z* calculated for $C_{32}H_{26}Cl_2F_2N_3O_4^+$ [M + H]$^+$: 624.12629, found 624.12560. **LC-MS** (ESI, C8, 0.5 mL/min) 65-95% MeCN in H_2O, 6 min, RT = 1.369 min, *m/z* = 624.1 [M + H]$^+$; 50-95% MeCN in H_2O, 6 min, RT = 4.067 min, *m/z* = 624.1 [M + H]$^+$.

*Neopentyl 4-(5-(4-chlorophenyl)-3-(4-(4-chlorophenyl)-2-oxo-1,2-dihydroquinolin-3-yl)-4,5-dihydro-1H-pyrazol-1-yl)-3,3-difluoro-4-oxobutanoate (**2o**).* Synthesis was carried out according to general procedure for **2i** esterification using compound **2i** (100 mg, 0.175 mmol, 1.00 eq) and neopentyl alcohol (155 mg, 1.75 mmol, 10.0 eq). Purified via normal-phase column chromatography eluting along a gradient of 0-75% EtOAc in hexanes. Finally, fractions containing the desired product were then collected, concentrated under reduced pressure, and dried under high vacuum to yield a pale yellow solid (59.7 mg, 0.0932 mmol, 53% yield). **TLC** (30% EtOAc in DCM) R_f = 0.43. **^{1}H NMR** (400 MHz, $CDCl_3$) δ 12.85 (brs, 1H), 7.62 – 7.55 (m, 1H), 7.51 – 7.43 (m, 2H), 7.39 – 7.34 (m, 1H), 7.34 – 7.27 (m, 3H), 7.23 – 7.18 (m, 3H), 7.04 – 6.98 (m, 2H), 5.48 (dd, *J* = 11.7, 3.8 Hz, 1H), 3.84 (d, *J* = 10.5 Hz, 1H), 3.73

(dd, *J* = 18.5, 11.9 Hz, 1H), 3.70 (d, *J* = 10.5 Hz, 1H), 3.38 – 3.18 (m, 2H), 3.04 (dd, *J* = 18.3, 3.8 Hz, 1H), 0.86 (s, 9H). **^{13}C NMR** (100 MHz, $CDCl_3$) δ 166.4 (t, *J* = 7.0 Hz), 162.6, 159.3 (t, *J* = 30.7 Hz), 155.4, 152.1, 139.0, 138.4, 135.2, 133.9, 133.2, 132.2, 130.9, 130.4, 129.2 (2C), 129.0, 128.9, 128.1, 127.6 (2C), 123.5, 122.6, 120.2, 116.4, 114.6 (t, *J* = 252.7 Hz), 74.8, 60.6, 44.8, 41.1 (t, *J* = 25.8 Hz), 31.4, 26.4 (3C) (*29 out of 33 carbon signals observed due to chemical shift equivalence*). **^{19}F NMR** (376 MHz, $CDCl_3$) δ -99.45 (dt, *J* = 275.3, 15.3 Hz), -100.28 (dt, *J* = 275.1, 15.3 Hz). **HRMS** (NSI) *m/z* calculated for $C_{33}H_{30}Cl_2F_2N_3O_4^+$ $[M + H]^+$: 640.15759, found 640.15688. **LC-MS** (ESI, C8, 0.5 mL/min) 70-95% MeCN in H_2O, 6 min, RT = 1.414 min, *m/z* = 640.1 $[M + H]^+$; 55-95% MeCN in H_2O, 6 min, RT = 4.151 min, *m/z* = 640.1 $[M + H]^+$.

*Benzyl 4-(5-(4-chlorophenyl)-3-(4-(4-chlorophenyl)-2-oxo-1,2-dihydroquinolin-3-yl)-4,5-dihydro-1H-pyrazol-1-yl)-3,3-difluoro-4-oxobutanoate (**2p**).* Synthesis was carried out according to general procedure for **2i** esterification using **2i** (620 mg, 1.09 mmol, 1.00 eq) and benzyl alcohol (226 µL, 2.17 mmol, 2.00 eq). Purified via normal-phase column chromatography eluting along a gradient of 0-80% EtOAc in hexanes. Fractions containing the desired product were subsequently collected, concentrated under reduced pressure, and purified a second time by normal-phase column chromatography eluting along a gradient of 0-50% EtOAc in DCM. Finally, fractions containing the desired product were once more collected, concentrated under reduced pressure, and dried under vacuum to yield a yellow foamy solid (297 mg, 0.450 mmol, 41% yield). **TLC** (30% EtOAc in DCM) R_f = 0.44. **^{1}H NMR** (400 MHz, $CDCl_3$) δ 12.70 (s, 1H), 7.61 – 7.53 (m, 1H), 7.47 – 7.40 (m, 2H), 7.38 – 7.26 (m, 8H), 7.25 – 7.23 (m, 1H), 7.22 – 7.15 (m, 3H), 7.02 – 6.95 (m, 2H), 5.39 (dd, *J* = 11.7, 4.0 Hz, 1H), 5.13 (d, *J* = 12.3 Hz, 1H), 5.07 (d, *J* = 12.3 Hz, 1H), 3.68 (dd, *J* = 18.2, 11.7 Hz, 1H), 3.39 – 3.19 (m, 2H), 3.01 (dd, *J* = 18.3, 4.0 Hz, 1H). **^{13}C NMR** (151 MHz, $CDCl_3$) δ 166.1 (t, *J* = 7.2 Hz), 162.4, 159.2 (t, *J* = 30.2 Hz), 155.4, 152.1, 138.9, 138.4, 135.3, 135.2, 133.9, 133.3, 132.2, 130.9, 130.3, 129.14 (2C), 129.05, 128.9, 128.7 (2C), 128.5, 128.4 (2C), 128.1, 127.5 (2C), 123.6, 122.6, 120.2, 116.3, 114.5 (t, *J* = 253.2 Hz), 67.2, 60.6,

44.8, 41.1 (t, J = 25.9 Hz) (*31 out of 35 carbon signals observed due to chemical shift equivalence*). **^{19}F NMR** (376 MHz, Chloroform-*d*) δ -99.28 (ddd, J = 273.6, 16.5, 13.7 Hz), -100.34 (ddd, J = 274.0, 16.3, 13.9 Hz). **HRMS** (APCI) *m/z* calculated for $C_{35}H_{26}Cl_2F_2N_3O_4^+$ [M + H]$^+$: 660.12629, found 660.12522. **LC-MS** (ESI, C8, 0.5 mL/min) 65-95% MeCN in H_2O, 6 min, RT = 1.479 min, *m/z* = 660.1 [M + H]$^+$; 50-95% MeCN in H_2O, 6 min, RT = 4.276 min, *m/z* = 660.1 [M + H]$^+$.

*4-(5-(4-Chlorophenyl)-3-(4-(4-chlorophenyl)-2-oxo-1,2-dihydroquinolin-3-yl)-4,5-dihydro-1H-pyrazol-1-yl)-3,3-difluoro-N-methyl-4-oxobutanamide (**2q**)*. Synthesis was carried out according to general procedure for **2i** amidation using compound **2i** (300 mg, 0.526 mmol, 1.00 eq) and methylamine solution (2.0 M in THF, 789 μL, 1.58 mmol, 3.00 eq). Purified via normal-phase column chromatography eluting along a gradient of 0-100% EtOAc in DCM followed by 0-1% MeOH in DCM. Finally, fractions containing the desired product were collected, concentrated under reduced pressure, and dried under vacuum to yield a yellow foamy solid (225 mg, 0.386 mmol, 73% yield). **TLC** (10% MeOH in DCM) R_f = 0.29. **^{1}H NMR** (500 MHz, $CDCl_3$) δ 12.43 (s, 1H), 7.61 – 7.54 (m, 1H), 7.49 – 7.42 (m, 2H), 7.37 – 7.27 (m, 4H), 7.24 – 7.15 (m, 3H), 7.00 – 6.94 (m, 2H), 6.07 (q, J = 4.1, 3.7 Hz, 1H), 5.47 (dd, J = 11.6, 3.9 Hz, 1H), 3.66 (dd, J = 18.3, 11.7 Hz, 1H), 3.11 (t, J = 16.2 Hz, 2H), 2.96 (dd, J = 18.3, 4.0 Hz, 1H), 2.76 (d, J = 4.8 Hz, 3H). **^{13}C NMR** (151 MHz, $CDCl_3$) δ 165.3 (t, J = 5.4 Hz), 162.2, 159.7 (t, J = 30.7 Hz), 155.6, 152.2, 138.8, 138.4, 135.3, 134.0, 133.2, 132.3, 131.0, 130.4, 129.21 (2C), 129.17, 128.9, 128.1, 127.4 (2C), 123.6, 122.5, 120.1, 116.4, 115.2 (t, J = 253.5 Hz), 60.6, 44.8, 42.8 (t, J = 24.6 Hz), 26.7 (*27 out of 29 carbon signals observed due to chemical shift equivalence*). **^{19}F NMR** (376 MHz, $CDCl_3$) δ -98.63 (dt, J = 270.8, 16.6 Hz), -99.89 (dt, J = 270.5, 15.8 Hz). **HRMS** (APCI) *m/z* calculated for $C_{29}H_{23}Cl_2F_2N_4O_3^+$ [M + H]$^+$: 583.11098, found 583.11064. **LC-MS** (ESI, C8, 0.5 mL/min) 55-95% MeCN in H_2O, 6 min, RT = 1.130 min, *m/z* = 583.0 [M + H]$^+$; 35-95% MeCN in H_2O, 6 min, RT = 4.307 min, *m/z* = 583.0 [M + H]$^+$.

*4-(5-(4-Chlorophenyl)-3-(4-(4-chlorophenyl)-2-oxo-1,2-dihydroquinolin-3-yl)-4,5-dihydro-1H-pyrazol-1-yl)-3,3-difluoro-N,N-dimethyl-4-oxobutanamide (**2r**)*. Synthesis was carried out according to general procedure for **2i** amidation using **2i** (500 mg, 0.877 mmol, 1.00 eq) and dimethylamine solution (2.0 M in THF, 877 μL, 1.75 mmol, 2.00 eq). Purified via normal-phase column chromatography eluting along a gradient of 0-75% EtOAc in DCM. Next, fractions containing the desired product were subsequently collected, concentrated under reduced pressure, and purified by reversed-phase column chromatography eluting along a gradient of 0-75% MeOH in H_2O. Finally, fractions containing the desired product were once more collected, concentrated under reduced pressure, and dried under vacuum to yield a yellow foamy solid (275 mg, 0.461 mmol, 53% yield). **TLC** (10% MeOH in DCM) R_f = 0.31. ^{1}H NMR (400 MHz, $CDCl_3$) δ 12.93 (s, 1H), 7.62 – 7.54 (m, 1H), 7.50 – 7.40 (m, 2H), 7.40 – 7.35 (m, 1H), 7.35 – 7.30 (m, 1H), 7.30 – 7.24 (m, 2H), 7.22 – 7.14 (m, 3H), 7.05 – 6.97 (m, 2H), 5.52 (dd, *J* = 11.7, 3.9 Hz, 1H), 3.73 (dd, *J* = 18.2, 11.8 Hz, 1H), 3.41 – 3.26 (m, 1H), 3.26 – 3.10 (m, 1H), 3.01 (s, 3H), 2.98 (dd, *J* = 14.3, 4.0 Hz, 1H), 2.94 (s, 3H). **^{13}C NMR** (151 MHz, $CDCl_3$) δ 165.7 (t, *J* = 6.1 Hz), 162.5, 159.8 (t, *J* = 30.4 Hz), 154.7, 151.9, 139.2, 138.5, 135.1, 133.7, 133.3, 132.2, 131.2, 130.3, 129.12, 129.08 (2C), 128.8, 128.0, 127.5 (2C), 123.5, 122.8, 120.2, 116.5, 115.8 (dd, *J* = 253.1, 249.8 Hz), 60.6, 44.9, 39.6 (t, *J* = 25.0 Hz), 37.8, 35.5 (*28 out of 30 carbon signals observed due to chemical shift equivalence*). **^{19}F NMR** (376 MHz, $CDCl_3$) δ -99.19 (ddd, *J* = 269.1, 19.1, 13.1 Hz), -100.27 (ddd, *J* = 269.1, 15.3, 12.8 Hz). **HRMS** (APCI) *m/z* calculated for $C_{30}H_{25}Cl_2F_2N_4O_3^+$ [M + H]$^+$: 597.12663, found 597.12578. **LC-MS** (ESI, C8, 0.5 mL/min) 55-95% MeCN in H_2O, 6 min, RT = 1.312 min, *m/z* = 597.1 [M + H]$^+$; 35-95% MeCN in H_2O, 6 min, RT = 4.514 min, *m/z* = 597.1 [M + H]$^+$.

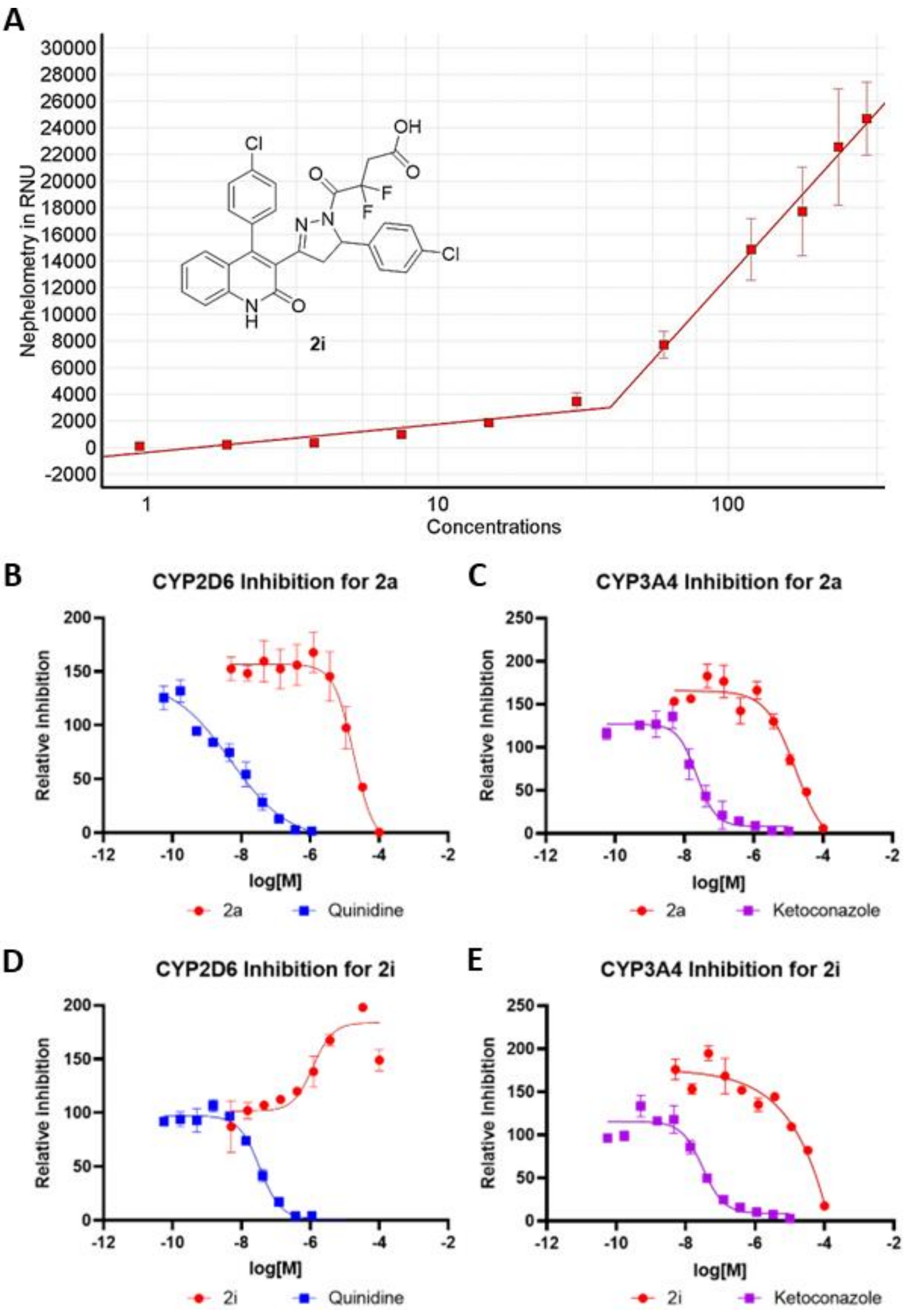

Figure S4: The kinetic solubility of **2i** (**DQP-997-74**) in PBS (1% DMSO) at room temperature was 38 ± 7.9 µM for **2i** (**A**) and *in vitro* CYP450 inhibition for **2a** (**B,C**) and **2i** (**D,E**) with quinidine (2D6) and ketoconazole (3A4).

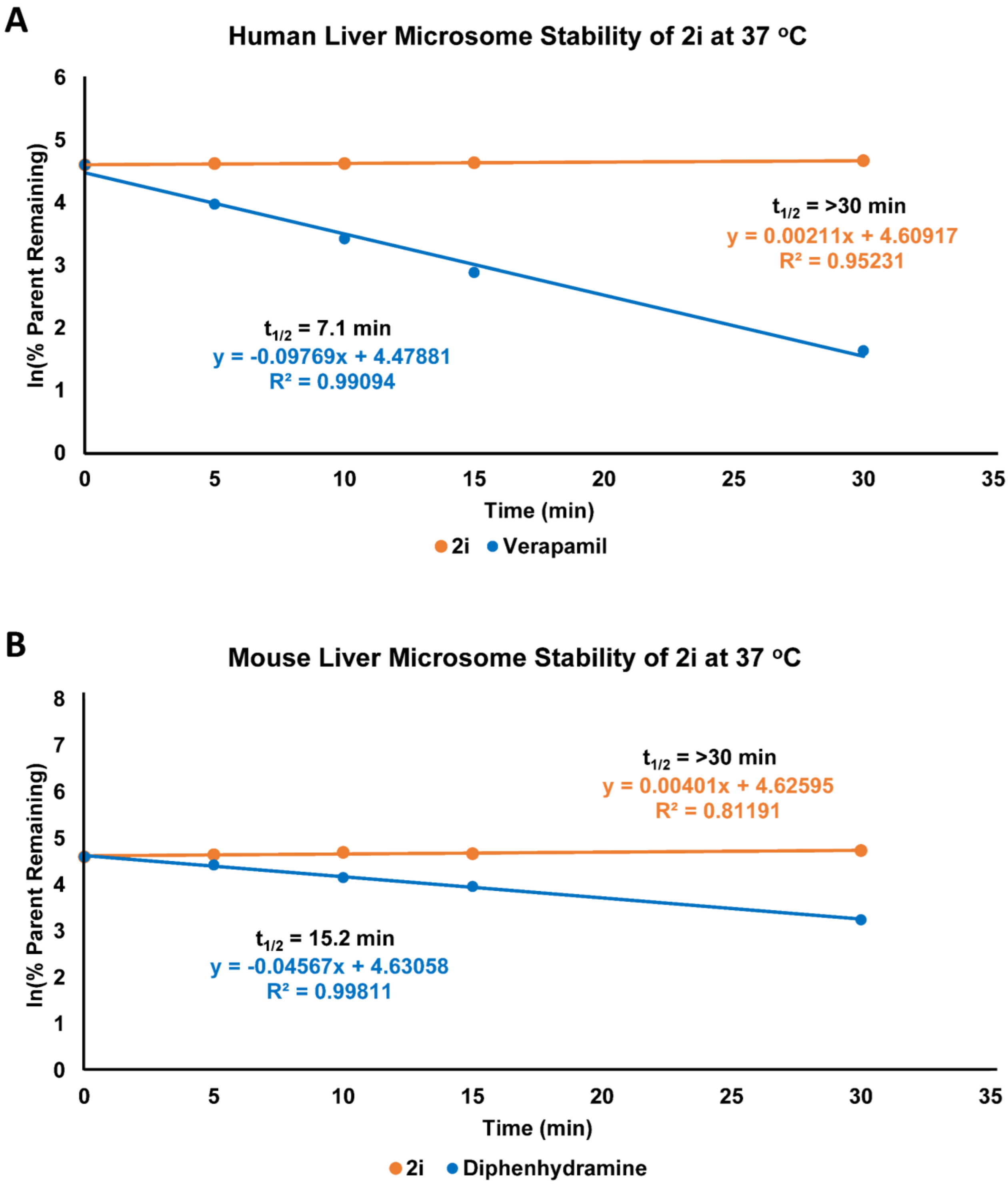

Figure S5: *In vitro* HLM stability of **2i** (**DQP-997-74**) (5 points over 30 min; verapamil positive control; **A**) and *in vitro* MLM stability of **2i** (5 points over 30 min; diphenhydramine positive control, **B**).

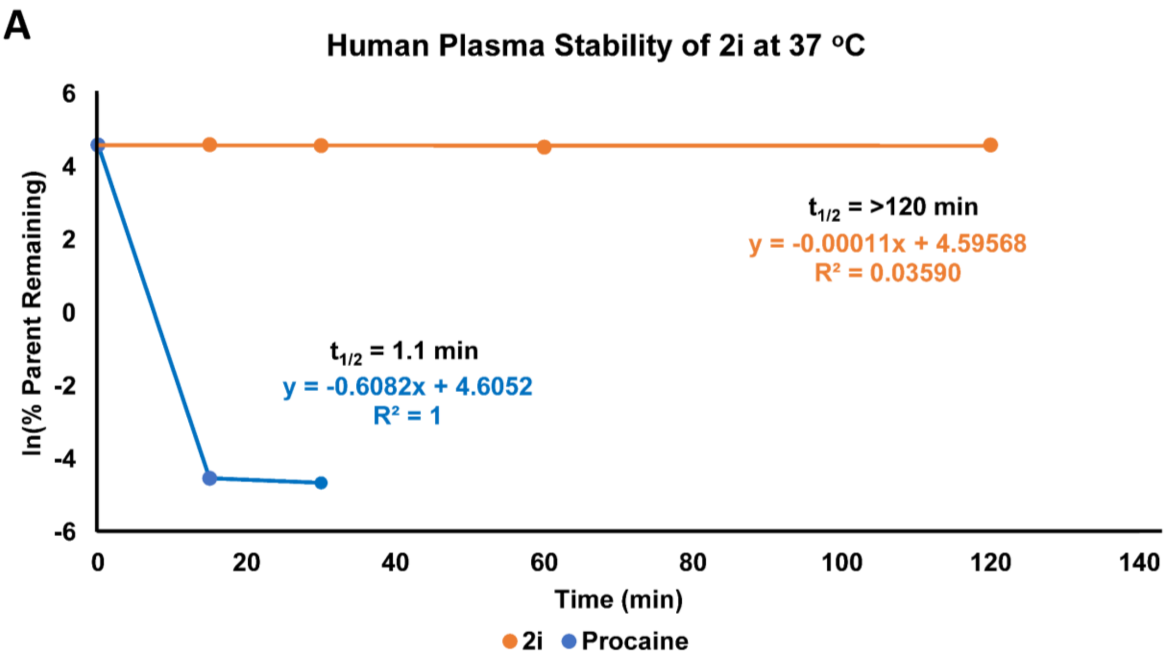

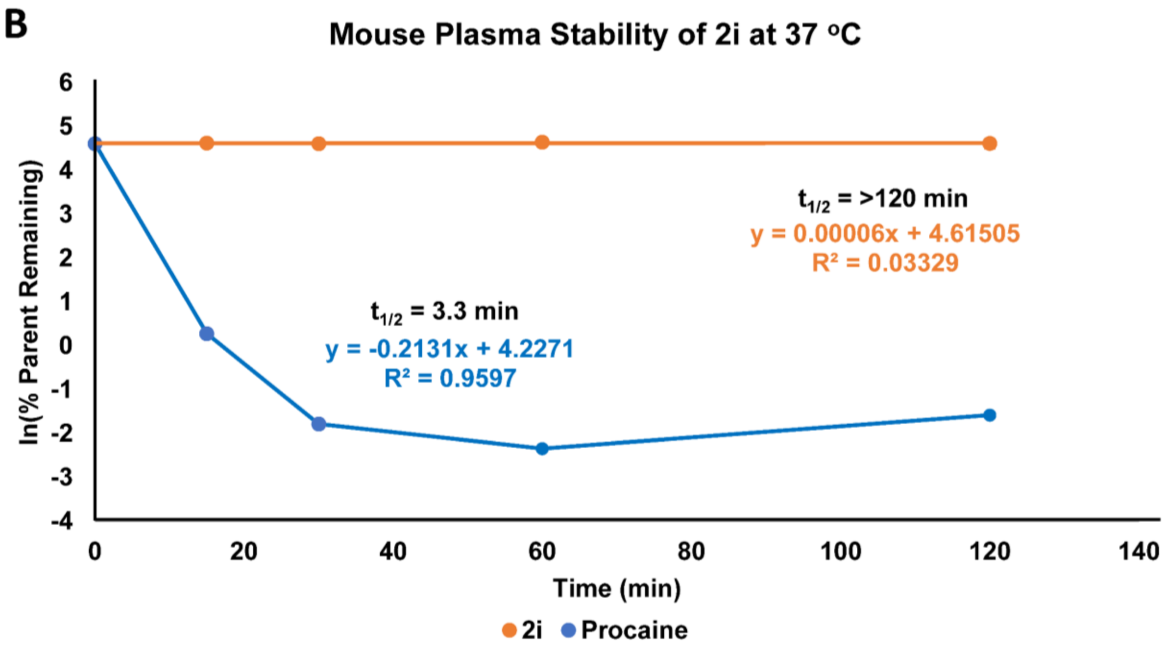

Figure S6: *In vitro* human plasma stability of **2i** (**DQP-997-74**) (5 points over 120 min; procaine positive control, **A**) and *in vitro* mouse plasma stability of **2a** (5 points over 120 min; procaine positive control **B**).

Column: Prep HPLC
Flow Rate: 10.4 ml/min
Equilibration Volume: 281.4 ml
Initial Waste: 0.0 ml
Solvent: A2 Water/0.1% FA
Solvent: B3 ACN/0.1%FA

Peak Tube Volume: Max.
Non-Peak Tube Volume: Max.
Loading Type:
Wavelength 1 (red): 214nm
Peak Width: 15 sec
Threshold: 0.10 AU
Wavelength 2 (purple): 254nm

Run Notes: Sample: 3.25 ml from tube 1
Prep HPLC Column: Chiralcel OD-RH Dimensions: 21 mm x 250 mm 5 µm

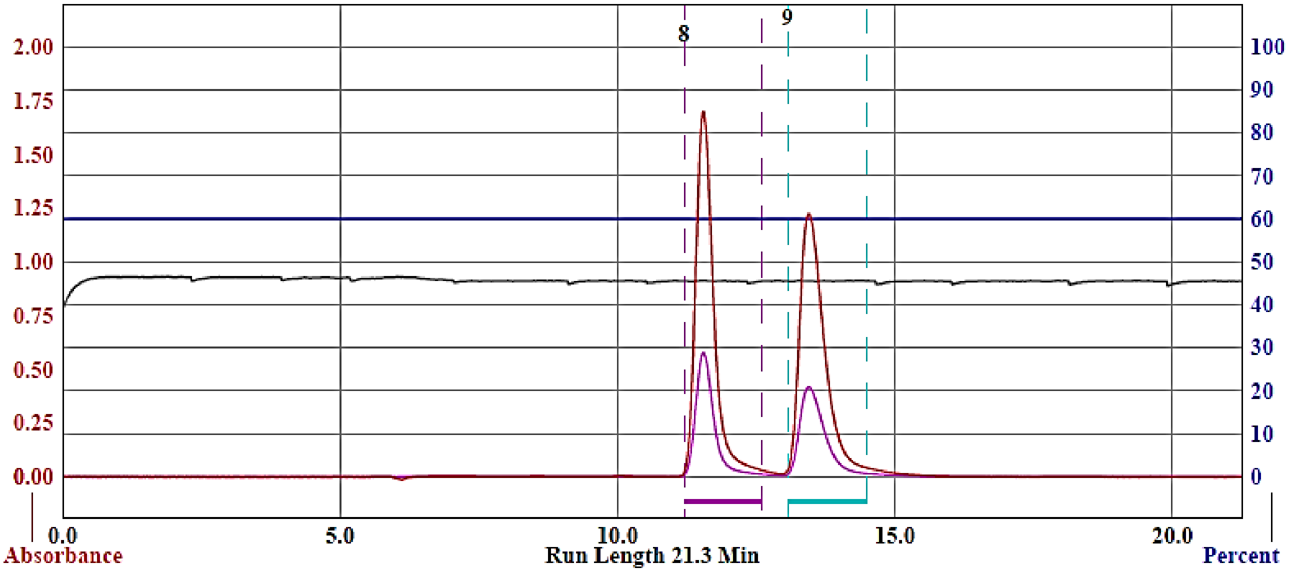

Figure S7. Chiral separation of **(*rac*)-2i** (**DQP-997-74**). Purification on a Daicel Chiralcel® OD-RH (21 mm x 250 mm, 5 µm) and a 5 mL sample loop with isocratic 60% CH_3CN in H_2O (0.1% FA) at 10.4 mL/min over 21.3 min (254 nm).

A

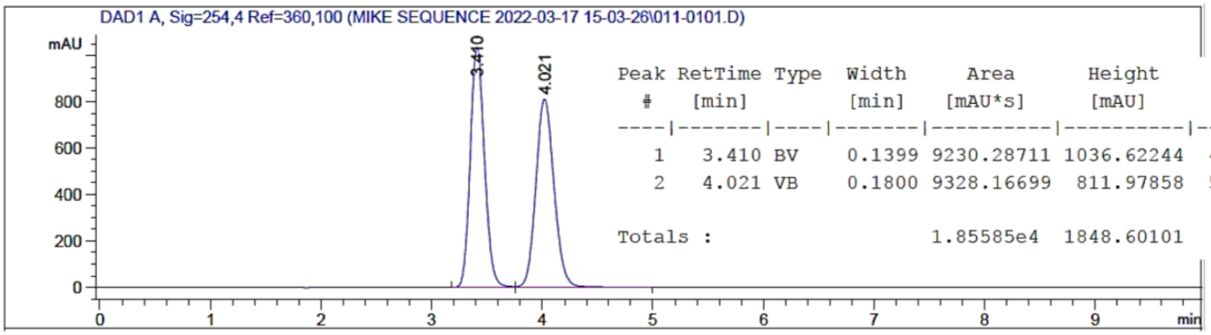

Peak #	RetTime [min]	Type	Width [min]	Area [mAU*s]	Height [mAU]	Area %
1	3.410	BV	0.1399	9230.28711	1036.62244	49.7363
2	4.021	VB	0.1800	9328.16699	811.97858	50.2637
Totals :				1.85585e4	1848.60101	

B

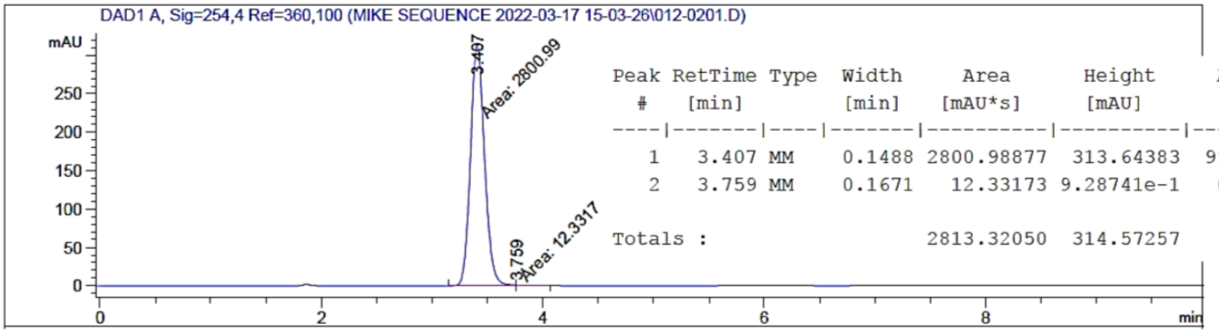

Peak #	RetTime [min]	Type	Width [min]	Area [mAU*s]	Height [mAU]	Area %
1	3.407	MM	0.1488	2800.98877	313.64383	99.5617
2	3.759	MM	0.1671	12.33173	9.28741e-1	0.4383
Totals :				2813.32050	314.57257	

C

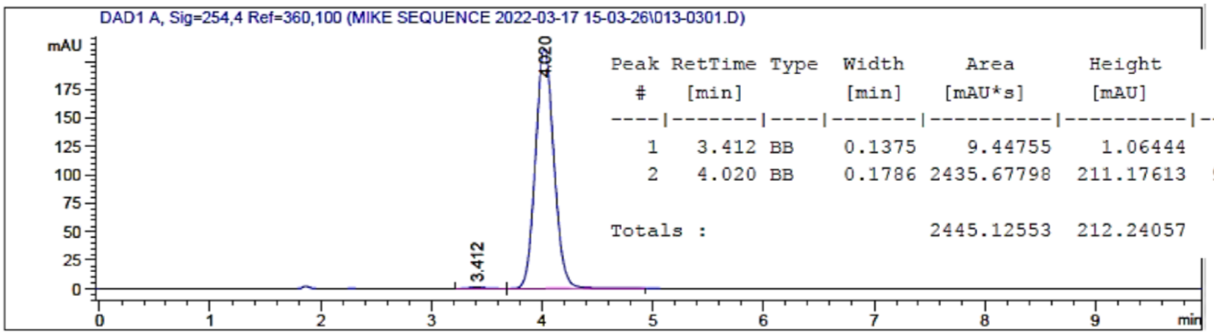

Peak #	RetTime [min]	Type	Width [min]	Area [mAU*s]	Height [mAU]	Area %
1	3.412	BB	0.1375	9.44755	1.06444	0.3864
2	4.020	BB	0.1786	2435.67798	211.17613	99.6136
Totals :				2445.12553	212.24057	

Figure S8. A. HPLC trace of **2i** (**DQP-997-74**) from a ChiralPAK® OD-RH (4.6 mm x 150 mm, 5 µm) column at 40 °C with isocratic 60% CH_3CN in H_2O (0.1% FA) at 1 mL/min over 10 min (254 nm) for racemic **2i** (**A**), **(*S*)-2i** (e.e. of 99% **B**), and **(*R*)-2i**. (e.e. of 99% **C**). Peak1 **(*S*)-2i**: c=1 in $CHCl_3$, $\propto_{589}^{20}$= -6.200; Peak 2, (*R*)-**2i**: c=1 in $CHCl_3$, $\propto_{589}^{20}$= +6.200

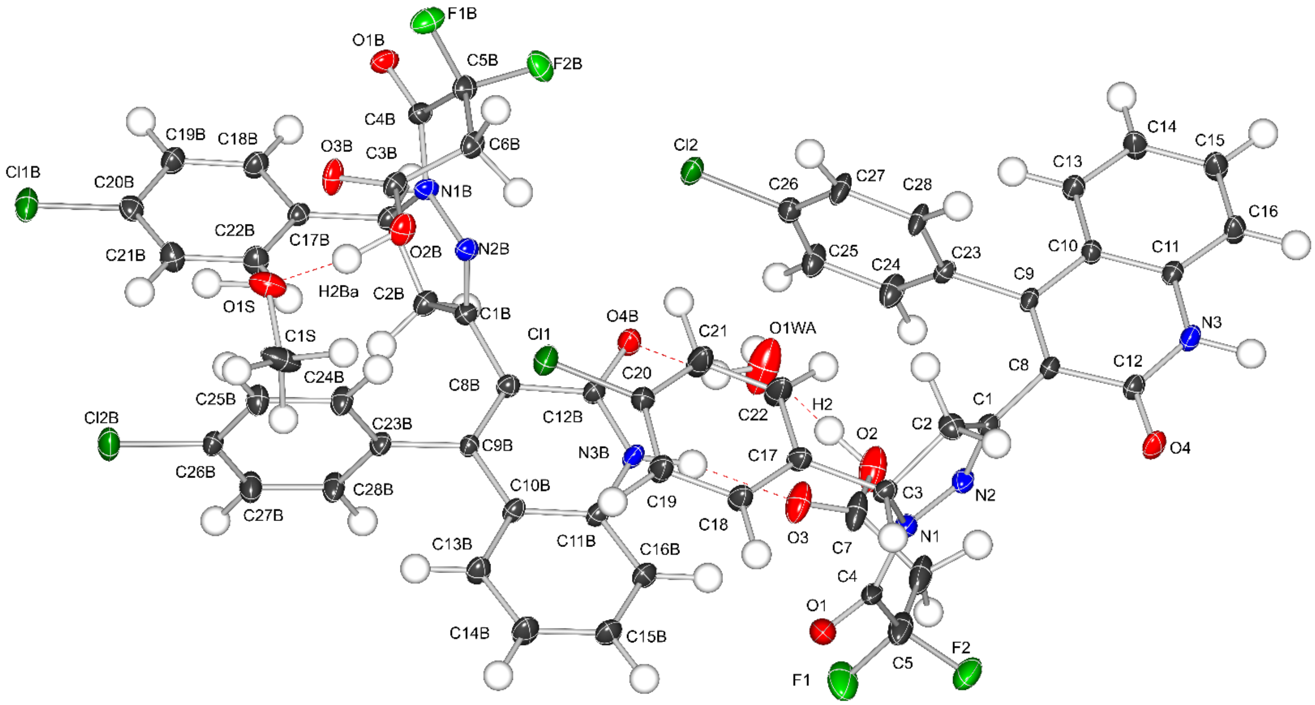

Figure S9: The crystal structure of (*S*)-**2i or** ((*S*)-**DQP-997-74**) with a value of Z' is 1 although there are two independent 'target' molecules in the asymmetric unit (formula $C_{28}H_{19}Cl_2F_2N_3O_4$, 1($CH_4O$), 0.5($H_2O$), 0.5($CH_3OH$)), as one molecule of methanol occupies one site, and the second site is mixed water and methanol (1:1 ratio).

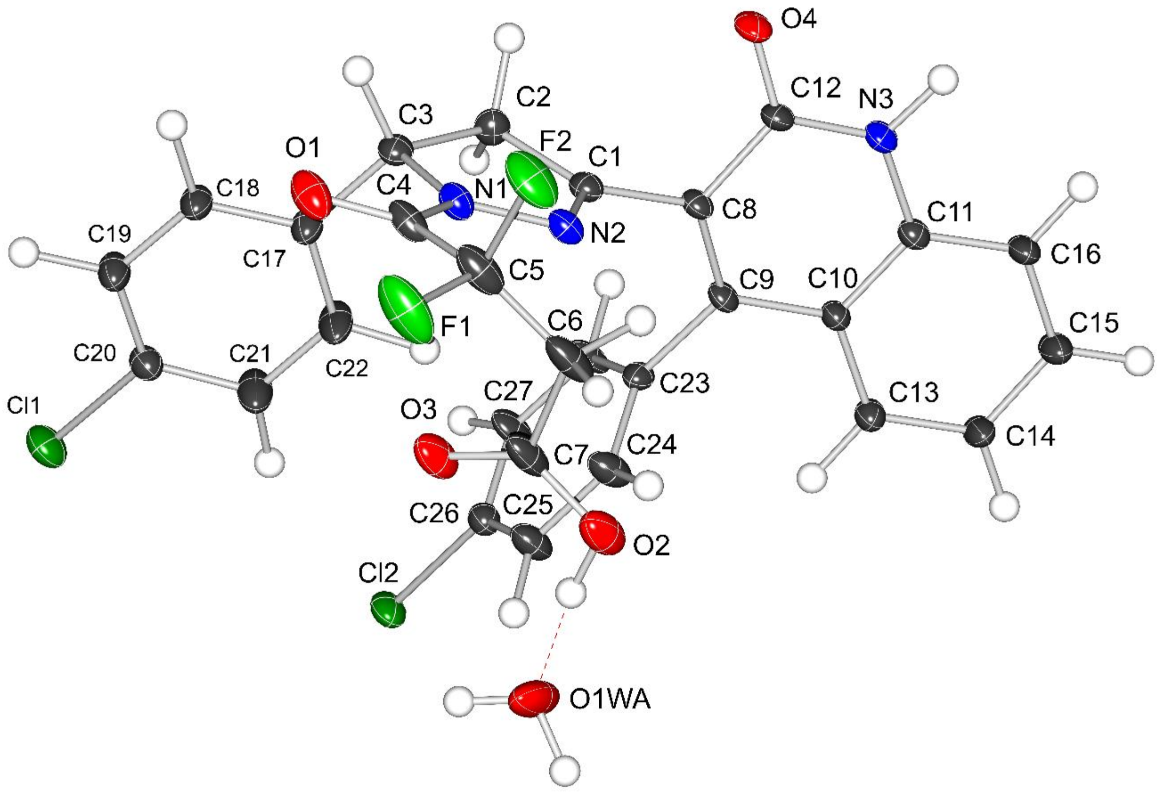

Figure S10: One of two independent 'target' molecules in the asymmetric unit hydrogen bonded to 50% water and 50% methanol occupying the same site (only the water molecule could be refined).

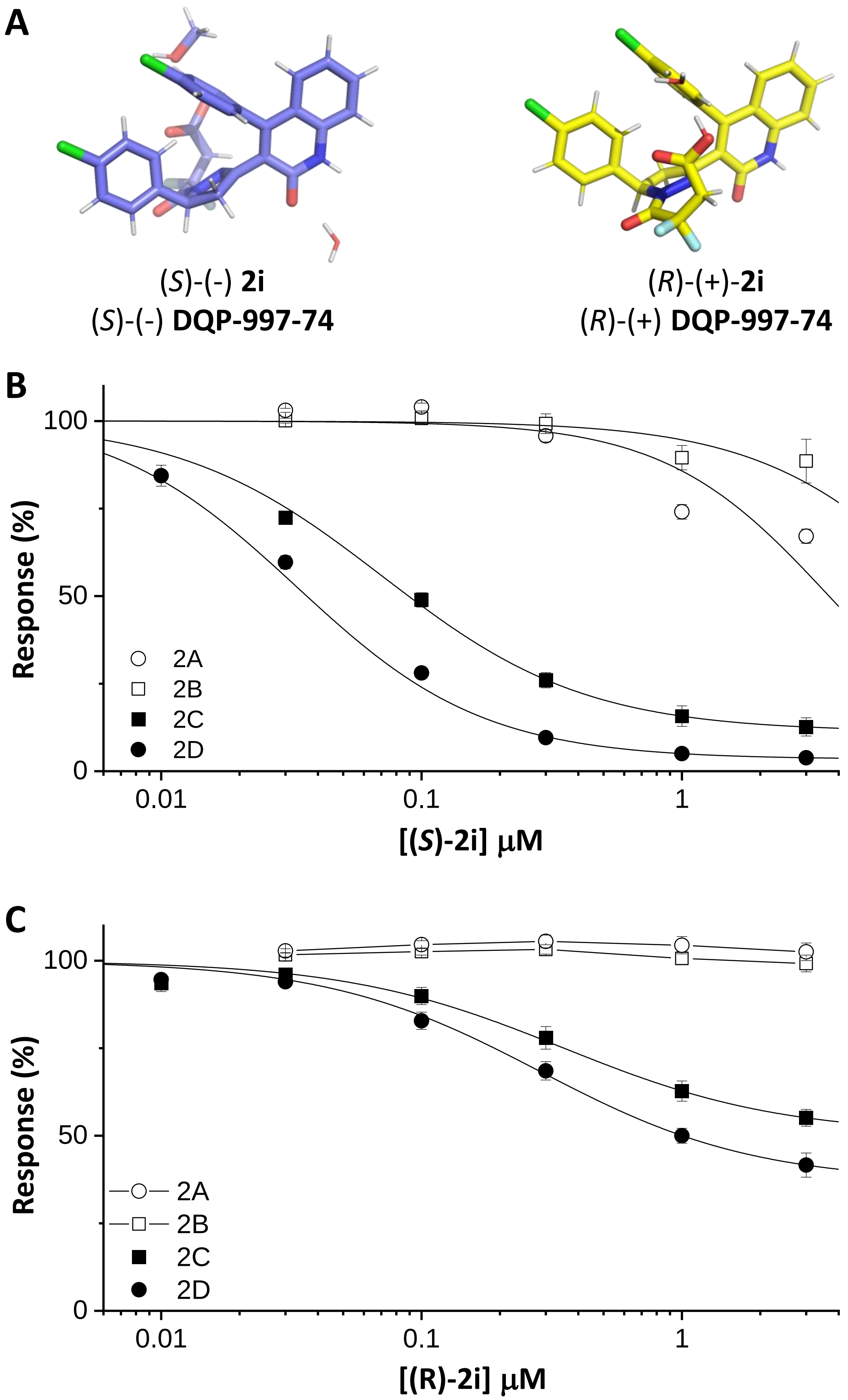

Figure S11. Structures (**A**) and the composite average concentration-response relationship for ***(S)*-2i** (**B**) and for ***(R)*-2i** (**C**) determined for GluN1/GluN2A-D NMDARs expressed in *Xenopus* oocytes (error bars SEM and shown when larger than symbol).

A

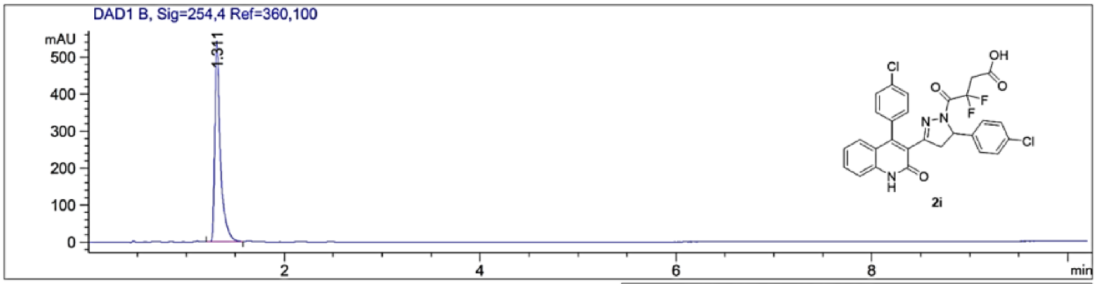

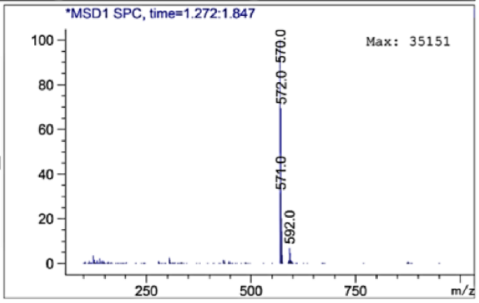

Signal 2: DAD1 B, Sig=254,4 Ref=360,100

Peak #	RetTime [min]	Type	Width [min]	Area [mAU*s]	Height [mAU]	Area %
1	1.311	BB	0.0571	2142.83594	541.79919	100.0000
Totals :				2142.83594	541.79919	

B

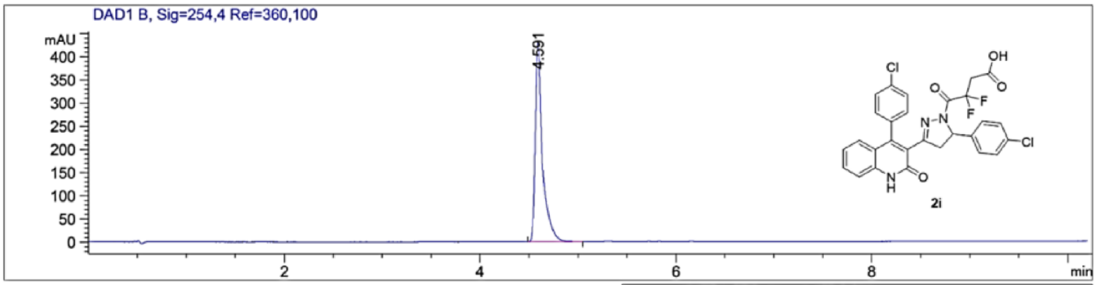

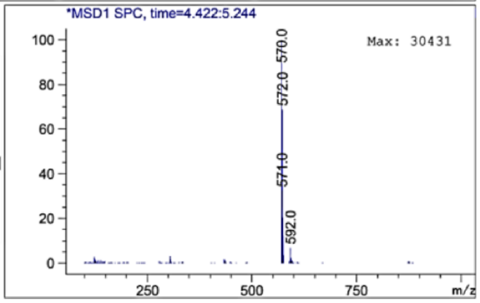

Signal 2: DAD1 B, Sig=254,4 Ref=360,100

Peak #	RetTime [min]	Type	Width [min]	Area [mAU*s]	Height [mAU]	Area %
1	4.591	BB	0.0724	2184.65723	432.44336	100.0000
Totals :				2184.65723	432.44336	

C

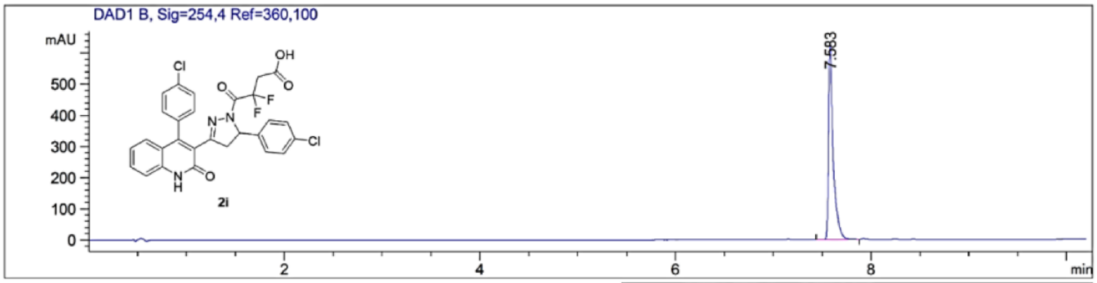

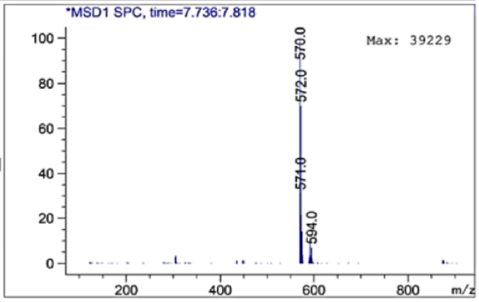

Signal 2: DAD1 B, Sig=254,4 Ref=360,100

Peak #	RetTime [min]	Type	Width [min]	Area [mAU*s]	Height [mAU]	Area %
1	7.583	BB	0.0471	2080.09229	636.58502	100.0000
Totals :				2080.09229	636.58502	

Figure S12. LC-MS traces for **2i** (**DQP-997-74**) from an Agilent InfinityLab Poroshell 120 EC-C8 (2.1 mm x 50 mm, 2.7 µm) column at 40 °C using (**A**) 65-95% CH_3CN in H_2O (0.1% FA) (**B**) 55-95% CH_3CN in H_2O (0.1% FA), (**C**) 40-95% CH_3CN in H_2O (0.1% FA) all over 10 min (254 nm).

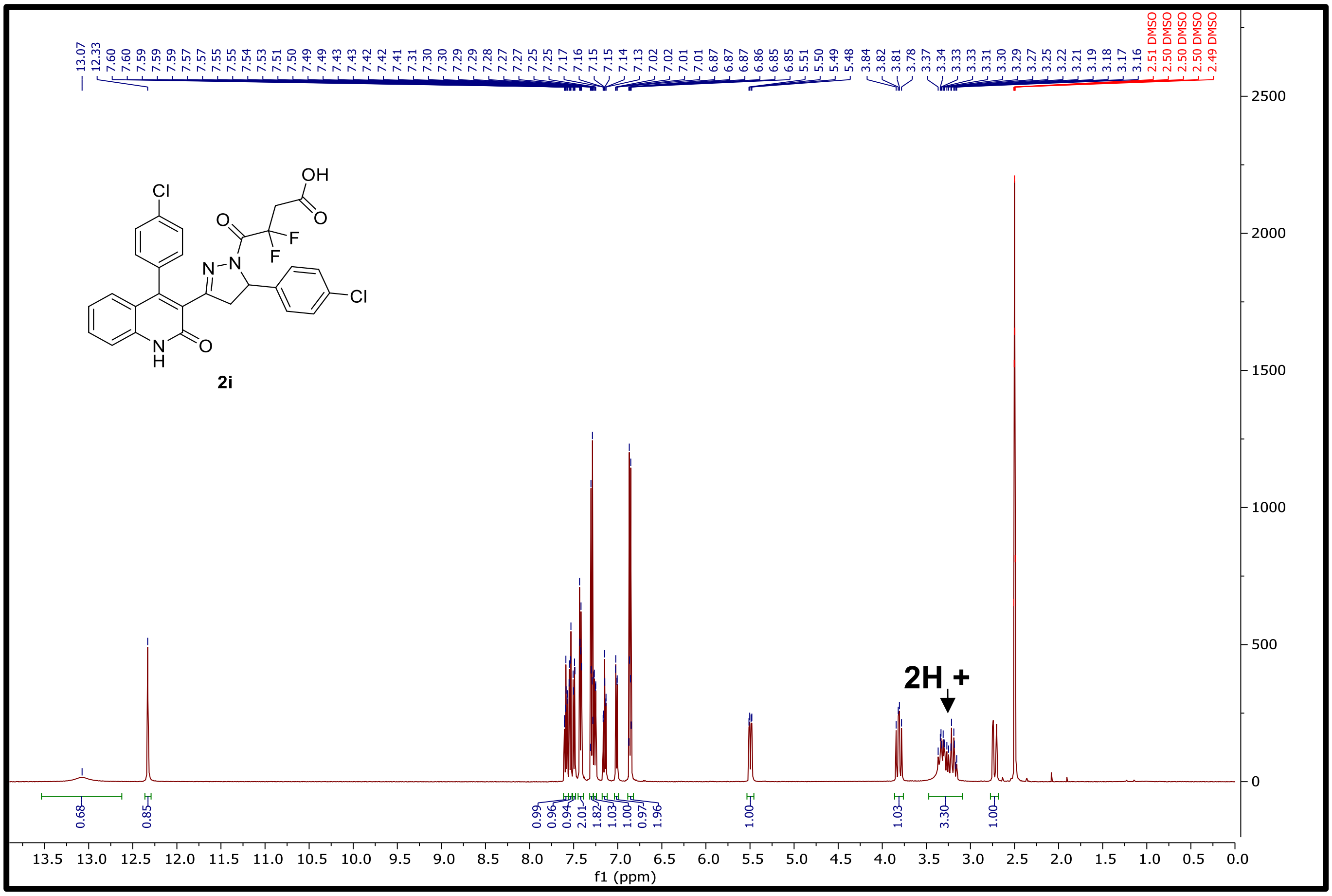

Figure S13. ^{1}H NMR for **2i** (**DQP-997-74**)

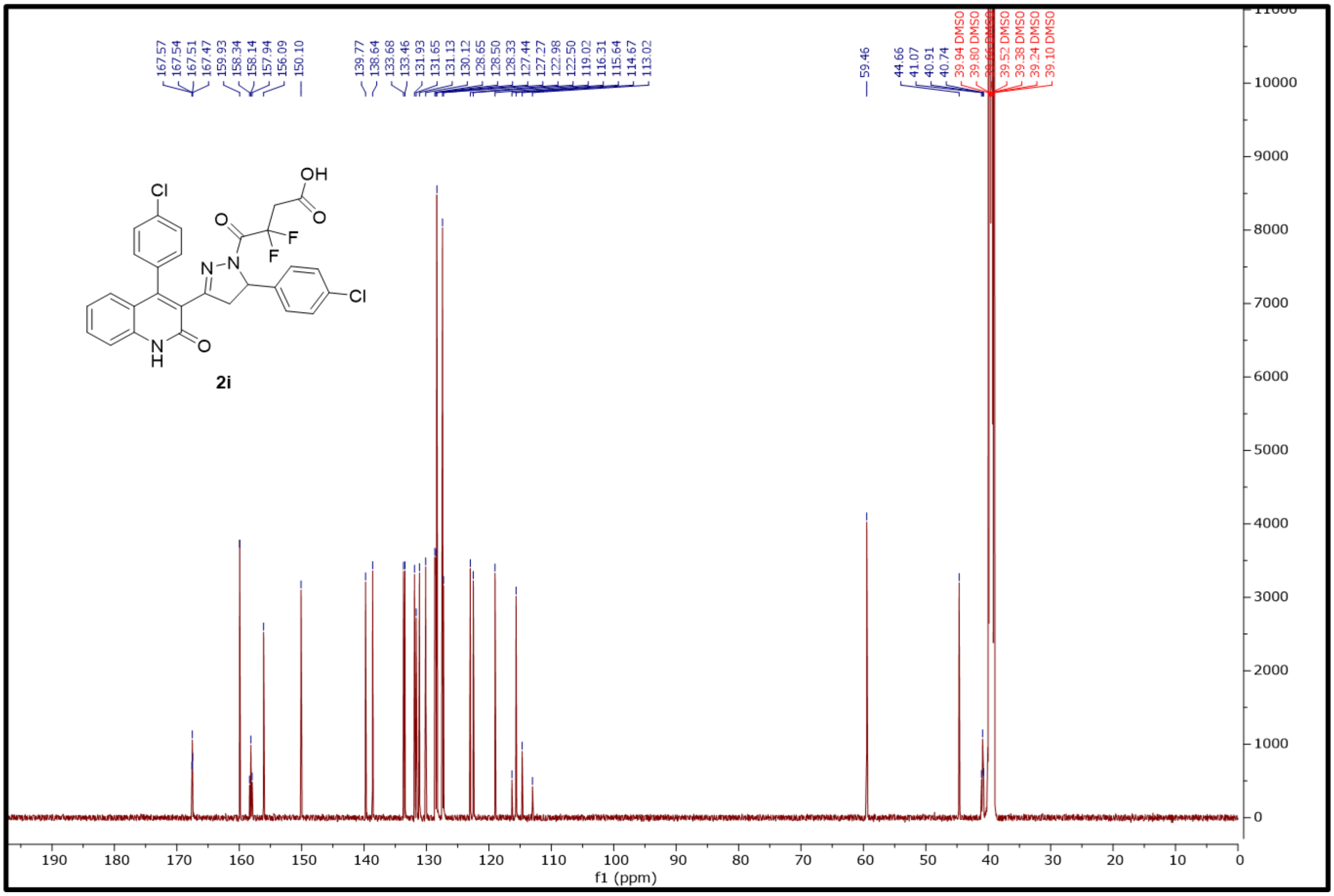

Figure S14. ^{13}C NMR for **2i** (**DQP-997-74**)

Table S1. *In Vitro* NMDAR Activity and Selectivity Profiles for DQP Analogs.

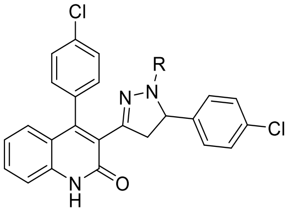

		Selectivity		$I_{30\,\mu M}$ / $I_{control}$ (mean ± SEM, %) (Number of experiments, oocytes)				IC50 (μM) [95% CI]			
	R	2A IC_{50} / 2D IC_{50}	2B IC_{50} / 2D IC_{50}	GluN2A	GluN2B	GluN2C	GluN2D	GluN2A	GluN2B	GluN2C	GluN2D
**2i<br>DQP-997-74**	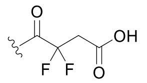	43	123	61 ± 2.4 [b]<br>(2,10)	78 ± 2.9 [b]<br>(2,10)	13 ± 2.5 [b]<br>(2,12)	12 ± 1.8 [b]<br>(2,16)	5.6<br>[4.7, 5.9]	16<br>[8.2,19]	0.35<br>[0.29, 0.40]	0.13<br>[0.087, 0.15]
**2j**	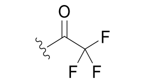	--	--	96 ± 2.1<br>(1.4)	86 ± 2.3<br>(1,4)	100 ± 2.2<br>(1,4)	86 ± 2.8<br>(1,4)	--	--	--	--
**2l**	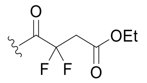	--	--	100 ± 3.2 [b]<br>(2,8)	97 ± 2.0 [b]<br>(2,8)	93 ± 0.7 [b]<br>(2,8)	87 ± 0.8 [b]<br>(2,8)	--	--	--	--
**2m**	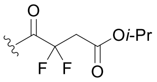	--	--	97 ± 2.3<br>(1,4)	88 ± 1.3<br>(1,4)	90 ± 4.3<br>(1,3)	92 ± 3.1<br>(1,4)	--	--	--	--
**2n**	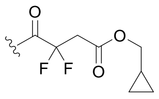	--	--	100 ± 1.3<br>(1,4)	92 ± 2.1<br>(1,4)	93 ± 4.2<br>(1,4)	92 ± 2.7<br>(1,4)	--	--	--	--
**2o**	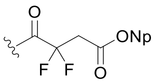	--	--	97 ± 2.2 [b]<br>(2,8)	94 ± 1.0 [b]<br>(2,8)	87 ± 0.8 [b]<br>(2,8)	82 ± 0.7 [b]<br>(2,8)	--	--	--	--
**2p**	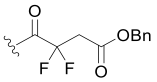	--	--	105 ± 0.72<br>(3,12)	103 ± 2.1<br>(2,10)	98 ± 0.95<br>(1,6)	96 ± 0.53<br>(1,6)	--	--	--	--

The mean ratio (±SEM) of the current response to a maximally effective concentration for glutamate (100 μM) and glycine (30 μM) in the presence and absence of 30 μM test compound is given (n=4-11 oocytes from 1-3 independent experiments). Fitted IC_{50} values for inhibition of responses to glutamate (100 μM) and glycine (30 μM) are shown to two significant figures when inhibition at 30 μM resulted in a response less than 70% of control. Values in brackets are 95% confidence intervals determined from the Log(IC_{50}). -- indicates not determined.

[a] Data for compound **2i** and **2l** were included from Table 1 for comparison.

[b] Compound **2i** was tested at 3 μM due to solubility limits; compound **2o** was tested at 10 μM due to solubility limits.

Table S2. Summary of mouse brain tissue binding and stability for test compounds and positive control.

Entry	Species	% Bound[a]			% Unbound	fu	% Recovery	% Compound Remaining at 4 hrs
		R1	R2	Mean ± SD				
Carbamazepine	Mice Brain (C57BL/6)	86.9	86.0	86.5 ± 0.6	13.5	0.135	91	105
**2a**		99.9	99.8	99.8 ± 0.0	0.2	0.002	100	94
**2i, DQP-997-74**		>99.9	>99.9	>99.9	<0.1	NA	57	105
**2k**		NR	NR	NR	NR	NA	41	44
**2l**		NR	NR	NR	NR	NA	41	43
**2m**		NR	NR	NR	NR	NA	51	56
**2n**		NR	NR	NR	NR	NA	29	25
**2o**		NR	NR	NR	NR	NA	44	53
**2p**		NR	NR	NR	NR	NA	33	31
**2q**		NR	NR	NR	NR	NA	30	33
**2r**		>99.9	>99.9	>99.9	<0.1	NA	90	93

[a] Undetectable instrument response in buffer chamber doesn't allow calculation of % bound due to high binding to tissue proteins. Hence, binding was reported as > 99.9% bound and < 0.01% unbound. NA: Not Applicable. NR: Not reported for those compounds which were unstable within the incubation duration (4 h).

Table S3. Structure and Predicted ADME Parameters for DQP Analogs and Prodrugs.[a]

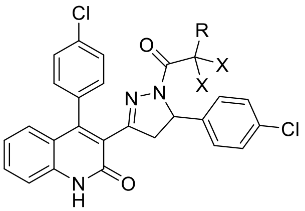

	R	X	MW	Rotatable Bonds	HBA	HBD	Consensus logP	tPSA (Å^2)
**2a**	CH_2CO_2H	H	534.39 (OH)<br>533.38 (O$^-$)	7	5	2 (OH)<br>1 (O$^-$)	4.69 (OH)<br>4.46 (O$^-$)	102.83 (OH)<br>105.66 (O$^-$)
**S1**	C_2H_4OH	H	520.41	7	4	2	4.87	85.76
**S2**	C_2H_4F	H	522.40	7	4	1	5.73	65.53
**2b**	$C_2H_4CO_2H$	H	548.42 (OH)<br>547.41 (O$^-$)	8	5	2 (OH)<br>1 (O$^-$)	4.91 (OH)<br>4.66 (O$^-$)	102.83 (OH)<br>105.66 (O$^-$)
**2d**	S-$CH_2CH(NH_2)CO_2H$	H	563.43	8	6 (U)<br>5 (Z)	3 (U)<br>2 (Z)	3.26 (U)<br>2.56 (Z)	128.85 (U)<br>133.30 (Z)
**2e**	CH_2CN	H	515.39	6	4	1	4.99	89.32
**2h**	$CH_2CH_2N_4$	H	558.42 (NH)<br>557.41 (N$^-$)	7	6 (NH)<br>7 (N$^-$)	2 (NH)<br>1 (N$^-$)	4.55 (NH)<br>4.42 (N$^-$)	119.99 (NH)<br>117.09 (N$^-$)
**2i**	CH_2CO_2H	F	570.37 (OH)<br>569.36 (O$^-$)	7	7	2 (OH)<br>1 (O$^-$)	5.09 (OH)<br>4.84 (O$^-$)	102.83 (OH)<br>105.66 (O$^-$)
**2j**	F	F	530.33	5	6	1	5.79	65.53
**2k**	CH_2CONH_2	F	569.39	7	6	2	4.75	108.62
**2l**	CH_2CO_2Et	F	598.43	9	7	1	5.85	91.83
**2m**	CH_2CO_2*i*-Pr	F	612.45	9	7	1	6.15	91.83
**2n**	$CH_2CO_2CH_2C_3H_5$	F	624.46	10	7	1	6.21	91.83
**2o**	CH_2CO_2Np	F	640.50	10	7	1	6.79	91.83
**2p**	CH_2CO_2Bn	F	660.49	10	7	1	6.67	91.83
**2q**	$CH_2CONHMe$	F	583.41	8	6	2	5.15	94.63
**2r**	CH_2CONMe_2	F	597.44	8	6	1	5.33	85.84

[a]ADME parameters were predicted using SwissADME web service from the Swiss Institute of Bioinformatics. Details and corresponding literature on methodologies and parameter validation can be found at http://www.swissadme.ch. U = Uncharged. Z = Zwitterion.

Table S4. Scan parameters and corresponding transitions in MRM mode for tested compounds.

	Precursor Ion MS1	Product Ion MS2	Fragmentor Voltage (V)	Collision Energy (V)	Cell Accelerator (V)	Polarity
**2a**	534.1	516.1<br>434.1<br>417.0	130	17<br>17<br>29	4	Positive
**2i<br>DQP-997-74**	570.1	417.0<br>402.0	170	29<br>25	4	Positive
**2k**	569.1	434.0<br>417.0	150	17<br>33	4	Positive
**2l**	598.1	532.1<br>417.0	160	21<br>33	4	Positive
**2m**	612.1	570.0<br>417.0	110	17<br>37	4	Positive
**2n**	624.1	569.7<br>416.8	160	17<br>33	4	Positive
**2o**	640.2	570.0<br>417.0	80	21<br>37	4	Positive
**2p**	660.1	91.1	150	50	4	Positive
**2q**	583.1	552.1<br>434.0<br>417.0	134	21<br>21<br>37	4	Positive
**2r**	597.1	434.0<br>164.0	148	17<br>25	4	Positive
**Diphenhydramine**	256.2	167.1<br>152.0	78	8<br>44	4	Positive
**Verapamil**	455.1	303.2<br>165.1	126	24<br>28	4	Positive
**Procaine**	237.2	120.0<br>100.2	98	25<br>13	4	Positive
**d_5-7-Ethoxycoumarin (ISTD)**	196.1	164.0	116	17	4	Positive

Table S5. Summary of **2i (DQP-997-74)** formation in mouse brain homogenate after 4 hours incubation for ester and amide prodrugs.

Entry	Sample Name	2i Peak Area	IS Peak Area	Calculated 2i Concentration (μM)	Average 2i Concentration (μM)
**2l**	Buffer_Mice_R1	0	659660	BLQ	BLQ
	Buffer_Mice_R2	0	627441	BLQ	
	Brain_Mice_R1	16657	649375	2.41	2.02
	Brain_Mice_R2	10991	641599	1.63	
	Brain_St_0Hr_Mice_R1	1291	614517	0.24	0.22
	Brain_St_0Hr_Mice_R2	1053	607774	0.2	
	Brain_St_4Hr_Mice_R1	16619	604507	2.58	2.66
	Brain_St_4Hr_Mice_R2	18625	638796	2.74	
**2m**	Buffer_Mice_R1	0	664118	BLQ	BLQ
	Buffer_Mice_R2	0	608989	BLQ	
	Brain_Mice_R1	5557	613535	0.88	0.84
	Brain_Mice_R2	5128	630636	0.8	
	Brain_St_0Hr_Mice_R1	147	630157	0.07	0.07
	Brain_St_0Hr_Mice_R2	145	599380	0.07	
	Brain_St_4Hr_Mice_R1	6901	619309	1.07	1.195
	Brain_St_4Hr_Mice_R2	8734	634097	1.32	
**2n**	Buffer_Mice_R1	0	527512	BLQ	BLQ
	Buffer_Mice_R2	0	475794	BLQ	
	Brain _Mice_R1	16150	450558	2.09	2.07
	Brain_Mice_R2	16785	478739	2.05	
	Brain_St_0Hr_Mice_R1	0	461533	BLQ	BLQ
	Brain_St_0Hr_Mice_R2	0	461582	BLQ	
	Brain_St_4Hr_Mice_R1	20939	452538	2.7	2.84
	Brain_St_4Hr_Mice_R2	23818	466146	2.98	
**2o**	Buffer_Mice_R1	0	510060	BLQ	BLQ
	Buffer_Mice_R2	0	514723	BLQ	
	Brain_Mice_R1	12636	511783	1.44	1.395
	Brain_Mice_R2	11179	485299	1.35	
	Brain_St_0Hr_Mice_R1	0	499645	BLQ	BLQ
	Brain_St_0Hr_Mice_R2	0	472205	BLQ	
	Brain_St_4Hr_Mice_R1	13908	470159	1.73	1.76
	Brain_St_4Hr_Mice_R2	14288	466230	1.79	

Entry	Sample Name	2i Peak Area	IS Peak Area	Calculated 2i Concentration (μM)	Average 2i Concentration (μM)
**2p**	Buffer_Mice_R1	0	486685	BLQ	BLQ
	Buffer_Mice_R2	0	477327	BLQ	
	Brain _Mice_R1	22911	465773	2.87	2.415
	Brain _Mice_R2	15915	473881	1.96	
	Brain_St_0Hr_Mice_R1	160	472668	0.03	0.0300
	Brain_St_0Hr_Mice_R2	0	493685	BLQ	
	Brain_St_4Hr_Mice_R1	22839	436698	3.05	2.99
	Brain_St_4Hr_Mice_R2	22437	447168	2.93	
**2q**	Buffer_Mice_R1	0	463660	BLQ	BLQ
	Buffer_Mice_R2	0	453769	BLQ	
	Brain _Mice_R1	0	455988	BLQ	BLQ
	Brain _Mice_R2	0	479030	BLQ	
	Brain_St_0Hr_Mice_R1	0	472944	BLQ	BLQ
	Brain_St_0Hr_Mice_R2	0	488611	BLQ	
	Brain_St_4Hr_Mice_R1	0	468546	BLQ	BLQ
	Brain_St_4Hr_Mice_R2	0	441692	BLQ	
**2r**	Buffer_Mice_R1	0	470852	BLQ	BLQ
	Buffer_Mice_R2	0	483973	BLQ	
	Brain _Mice_R1	0	450175	BLQ	BLQ
	Brain _Mice_R2	0	457327	BLQ	
	Brain_St_0Hr_Mice_R1	0	446150	BLQ	BLQ
	Brain_St_0Hr_Mice_R2	0	437237	BLQ	
	Brain_St_4Hr_Mice_R1	0	455969	BLQ	BLQ
	Brain_St_4Hr_Mice_R2	0	456918	BLQ	

BLQ: Below limit of quantification.

Table S6. Off-target actions of (*S*)-(-)-**2i**, or (*S*)-(-)-**DQP-997-74**

Receptor	Agonist	% control<br>1 μM compound (*S*)-(-)-2i [a]
**GluN1/GluN2A**	100 μM glutamate, 30 μM glycine	74 ± 2.1 (14)
**GluN1/GluN2B**	100 μM glutamate, 30 μM glycine	90 ± 3.5 (18)
**GluN1/GluN2C**	100 μM glutamate, 30 μM glycine	16 ± 2.9 (16)
**GluN1/GluN2D**	100 μM glutamate, 30 μM glycine	4.3 ± 0.73 (15)
**hGluN1-FA,TL/hGluN3A**	100 μM glycine	95 ± 2.3 (8)
**GluN1-4a/GluN3B**	100 μM glycine	97 ± 1.0 (8)
**GluA1**	100 μM glutamate	100 ± 2.0 (8)
**GluA2-R607Q**	100 μM glutamate	98 ± 0.85 (7)
**hGluA3-L531Y**	100 μM glutamate	105 ± 1.2 (8)
**GluK2**	100 μM glutamate	100 ± 1.0 (8)
**α4β2-nACh**	10 μM acetylcholine	91 ± 2.1 (5)
**α7-nACh**	300 μM acetylcholine	105 ± 13.5 (7)
**α1b2γ2σ-GABA**	100 μM GABA	85 ± 2.0 (5)
**ρ-GABA$_C$**	100 μM GABA	96 ± 1.2 (8)
**α1-Glycine**	100 μM glycine	103 ± 2.4 (8)
**hP2x**	9 μM ATP	108 ± 0.53 (8)

[a] Off target analysis was performed as described by Epplin et al. (2020; see Methods in main text). The mean ± standard error of the mean for agonist plus 1 μM (*S*)-(-)-**2i** as a percent of agonist in vehicle are given for each compound and receptor tested to two significant figures. Number of oocytes tested is given in parentheses.

Table S7. Summary of **2i** (**DQP-997-74**) plasma exposure after a single intraperitoneal injection (10 mg/kg)

**Plasma**	**IP**	10 mg/kg		Fed			
**Animal ID**	**C_{max} (ng/mL)**	**C_{max} (μM)**	**t_{max} (hr)**	**AUC_{LAST} (hr.ng/mL)**	**AUC_{INF} (hr.ng/mL)**	**$t1/2_{terminal}$ (hr)**	**AUC % Extrapolation**
Mean	1873	3.3	0.50	3854	4553	1.5	15

Table S8. Summary of **2i** (**DQP-997-74**) brain exposure after a single intraperitoneal injection (10 mg/kg)

**Brain**	**IP**	10 mg/kg		Fed				
**Animal ID**	**C_{max} (ng/g)**	**C_{max} (μM)**	**t_{max} (hr)**	**AUC_{LAST} (h*ng/g)**	**AUC_{INF} (h*ng/g)**	**$t1/2_{terminal}$ (hr)**	**AUC % Extrapolation**	**Ratio_AUC_{LAST} (Brain/plasma)**
Mean	23	0.040	0.50	64	151	NA	58	0.02

NA indicates not available. t1/2 was reported as NA since the Rsq < 0.75.

Table S9. Individual plasma concentration-time data of **2i** (**DQP-997-74**) following intraperitoneal injection (10 mg/kg)

**IP Plasma Exposure:**					
**Time (hr)**	**Concentration (ng/mL)**				
	**Group 1**	**Group 2**	**Group 3**	**Mean**	**SD**
0.25	1250.0	1500.0	1180.0	1310.0	168.2
0.5	2620.0	1260.0	1740.0	1873.3	689.7
1	1020.0	1270.0	1170.0	1153.3	125.8
2	1060.0	1140.0	1080.0	1093.3	41.6
4	163.0	208.0	585.0	318.7	231.7

Table S10. Individual brain concentration-time data of **2i** (**DQP-997-74**) following intraperitoneal injection (10 mg/kg)

**IP Brain Exposure:**					
**Time (h)**	**Calculated Concentration (ng/g)**				
	**Group 1**	**Group 2**	**Group 3**	**Mean**	**SD**
0.25	12.0	17.9	22.6	17.5	5.3
0.5	20.6	29.1	18.7	22.8	5.6
1	14.9	12.4	16.5	14.6	2.1
2	15.9	18.0	24.3	19.4	4.4
4	9.5	10.4	12.8	10.9	1.7

Table S11. Individual brain/plasma ratio-time data of **2i** (**DQP-997-74**) following intraperitoneal injection (10 mg/kg)

**IP Brain/plasma Ratio:**					
**Time (h)**	**Brain/plasma Ratio**				
	**Group 1**	**Group 2**	**Group 3**	**Mean**	**SD**
0.25	0.010	0.012	0.019	0.014	0.005
0.5	0.008	0.023	0.011	0.014	0.008
1	0.015	0.010	0.014	0.013	0.003
2	0.015	0.016	0.022	0.018	0.004
4	0.058	0.050	0.022	0.043	0.019

Table S12. Individual plasma concentration-time data of **2i** (**DQP-997-74**) following IV injection (5 mg/kg)

**IV Plasma Exposure**					
**Time (hr)**	**Concentration (ng/mL)**				
	**Group 1**	**Group 2**	**Group 3**	**Mean**	**SD**
0.25	575	585	766	642	108
1	41.2	101	68.7	70.4	30.0
3	10.2	6.78	5.97	7.64	2.22

Table S13. Individual brain concentration-time data of **2i** (**DQP-997-74**) following IV injection (5 mg/kg)

**IV Brain Exposure:**		BLQ indicates below 2 ng/g, assumed to be 0.0			
**Time (h)**	**Calculated Concentration (ng/g)**				
	**Group 1**	**Group 2**	**Group 3**	**Mean**	**SD**
0.25	10.8	17.9	13.1	13.9	3.66
1	BLQ	6.21	6.18	4.13	3.58
3	BLQ	BLQ	BLQ	--	--

References

Acker, T. M.; Yuan, H. J.; Hansen, K. B.; Vance, K. M.; Ogden, K. K.; Jensen, H. S.; Burger, P. B.; Mullasseril, P.; Snyder, J. P.; Liotta, D. C.; et al. Mechanism for Noncompetitive Inhibition by Novel GluN2C/D N-Methyl-D-aspartate Receptor Subunit-Selective Modulators. *Molecular Pharmacology* **2011**, *80* (5), 782-795. DOI: 10.1124/mol.111.073239.

Acker, T. M.; Khatri, A.; Vance, K. M.; Slabber, C.; Bacsa, J.; Snyder, J. P.; Traynelis, S. F.; Liotta, D. C. Structure-Activity Relationships and Pharmacophore Model of a Noncompetitive Pyrazoline Containing Class of GluN2C/GluN2D Selective Antagonists. *Journal of Medicinal Chemistry* **2013**, 56 (16), 6434-6456. DOI: 10.1021/jm400652r.

Bauer, J.O. and Gotz, T. Chloropentaphenyldisiloxane—Model Study on Intermolecular Interactions in the Crystal Structure of a Monofunctionalized Disiloxane. *Chemistry* **2021**, 3(2), 444-453; https://doi.org/10.3390/chemistry3020033

Bourhis, L.J.; Dolomanov, O.V.; Gildea, R.J.; Howard, J.A. and Puschmann, H. The anatomy of a comprehensive constrained, restrained refinement program for the modern computing environment - Olex2 dissected. Acta *Crystallogr A Found Adv*. **2015**, 71(Pt 1):59-75. DOI: 10.1107/S2053273314022207. PMID: 25537389; PMCID: PMC4283469.

Dolomanov, O.V.; Bourhis, L.J.; Gildea, R.J.; Howard, J.A.K. and Puschmann, H. OLEX2: A Complete Structure Solution, Refinement and Analysis Program. *Journal of Applied Crystallography*, **2009**, 42, 339-341. http://dx.doi.org/10.1107/S0021889808042726

Epplin, M.P.; Mohan, A., Harris; L.D., Zhu, Z.; Strong, K.L.; Bacsa, J.; Le, P.; Menaldino, D.S.; Traynelis, S.F.; Liotta, D.C. Discovery of Dihydropyrrolo[1,2-a]pyrazin-3(4H)-one-Based Second-Generation GluN2C- and GluN2D-Selective Positive Allosteric Modulators (PAMs) of the N-Methyl-d-Aspartate (NMDA) Receptor. *J Med Chem*. **2020**, 63(14), 7569-7600. DOI: 10.1021/acs.jmedchem.9b01733. PMID: 32538088

Sheldrick, G.M. SHELXT - integrated space-group and crystal-structure determination. *Acta Crystallogr A Found Adv*. **2015,** 71(Pt 1), 3-8. DOI: 10.1107/S2053273314026370. PMID: 25537383; PMCID: PMC4283466.
